# Uncovering heterogeneous intercommunity disease transmission from neutral allele frequency time series

**DOI:** 10.1073/pnas.2500663122

**Published:** 2025-11-26

**Authors:** Takashi Okada, Giulio Isacchini, QinQin Yu, Oskar Hallatschek

**Affiliations:** ^a^Department of Physics, University of California, Berkeley, CA 94720; ^b^Institute for Life and Medical Sciences, Kyoto University, Kyoto 606-8507, Japan; ^c^Interdisciplinary Theoretical and Mathematical Sciences Program, RIKEN, Wako 351-0198, Japan; ^d^Peter Debye Institute for Soft Matter Physics, Leipzig University, Leipzig 04103, Germany; ^e^Department of Immunology and Infectious Diseases, Harvard T. H. Chan School of Public Health, Boston, MA 02115; ^f^Department of Integrative Biology, University of California, Berkeley, CA 94720

**Keywords:** COVID-19, spatial epidemiology, genetic variation, human mobility

## Abstract

The COVID-19 pandemic has underscored the need for accurate epidemic forecasting to predict pathogen spread, evolution, and evaluate intervention strategies. Forecast reliability hinges on detailed knowledge of disease transmission across population segments, which may be inferred from contact surveys or mobility data. However, these indirect approaches make it difficult to estimate rare transmissions between socially or geographically distant communities. We show that the steep ramp-up of genome sequencing surveillance during the pandemic can be leveraged to directly identify transmission patterns between geographically defined communities. Our approach uses a hidden Markov model to infer the fraction of infections a community imports from others based on how rapidly allele frequencies in the focal community converge to those in the donor communities. Applying this method to SARS-CoV-2 sequencing data from England and the United States, we uncover networks of intercommunity transmission that reflect geographical relationships while exposing significant long-range interactions. The scaling of importation rate with distance is consistent across both countries, yet weaker than expected based on mobility data, highlighting limitations of indirect inference. We show that transmission patterns can change between waves of variants of concern and analyze how the inferred heterogeneity in intercommunity transmission impacts evolutionary forecasts. While applied here to geographically defined communities, our approach could be applied to those defined by other traits (e.g., age, socioeconomic status), provided time-series data can be stratified accordingly. Overall, our study highlights population genomic time series data as a crucial record of epidemiological interactions, which can be deciphered using tree-free inference methods.

## Significance

Current methods for understanding disease transmission between communities rely on indirect data, such as mobility patterns and contact surveys, which introduce significant uncertainty-especially when regions differ in policies, immunity, and travel patterns. To overcome these limitations, we introduce a computational framework that directly infers disease importation rates using genomic data alone. Applied to SARS-CoV-2, our method reveals transmission pathways, highlighting the importance of long-range interactions and their shifts across variant waves. By revealing how infections move among communities, this method enhances epidemic forecasting and explains why certain regions contribute disproportionately to pathogen evolution. While further validation is needed, it may also be applicable beyond SARS-CoV-2, including to other genomic data such as microbiome or ancient DNA studies.

Despite extensive efforts to model epidemiological dynamics, particularly during the COVID-19 pandemic, accurately predicting epidemic trajectories remains challenging, especially when transmission patterns vary widely across different communities (1–5). These differences arise from many factors, including varying population densities, mobility patterns, immunity levels, behaviors, and nonpharmaceutical interventions. While the ensuing transmission rate variations are difficult to anticipate, ignoring them reduces the applicability of model-based forecasts and may result in misguided interventions (6–9).

In principle, metapopulation models can account for known heterogeneities in the host population by dividing it into suitably many subpopulations, which are distinguished by their epidemiological characteristics. A crucial input to these models is a matrix of parameters that represents the rates at which infections are transmitted between subpopulations (10–12). Metapopulation models can be used to predict the impact of heterogeneities on disease spreading and evolution. By perturbing the transmission parameters, they also allow for the exploration of group-specific nonpharmaceutical interventions, immunity, or behavioral changes.

As the number *n* of subpopulations grows, estimating the full set of *n*_2_ transmission rates becomes increasingly challenging. Valuable clues about these rates have been gleaned from measuring how often members of different groups come in contact. For example, the real-time tracking of cell phones enables estimating the mobility flux between different regions (13–15) and surveys can be used to infer the mixing between different age groups (16, 17). However, converting these fluxes into transmission rates requires additional assumptions about the infection dynamics during contact. For example, how precisely variations in mask-wearing or immunity levels lower transmission rates is difficult to measure directly (18, 19), thus, requiring estimation methods. Furthermore, differences in local interventions and individual behaviors can weaken the relationship between mobility metrics and transmission rates, thereby reducing their predictive power (20).

To transcend these limitations, a direct data-driven approach to infer heterogeneous disease transmission rates is needed. Ground truths would be valuable even retrospectively, as they could be used to falsify transmission rates obtained from indirect methods and, more broadly, to develop improved evidence-based forecasting of epidemic spread and selective sweeps of new variants. Last, knowledge of how infections cross population boundaries can also inform phylogenetic approaches to embed genealogies of past outbreaks into geographical space, which are usually based on the assumption that lineages follow unbiased random walks (21, 22).

We argue that the steep ramp up of the surveillance of virus sequence variants during the COVID-19 pandemic offers unprecedented opportunities to use population genetic tools to obtain a direct view of the underlying metapopulation transmission network. Numerous studies have already demonstrated that the related effort of molecular source attribution (23–25) substantially gains in precision by the abundance of data. For example, embedding the phylogenetic tree of the sampled viruses within the geographical landscape of England has allowed for the reconstruction of detailed spatiotemporal infection processes for different variants of concern (26, 27). Nonetheless, inferring actual infection processes necessitates making significant assumptions about the dispersal of lineages (21, 22). Furthermore, phylogenetic methods require constructing genealogical trees, which is computationally challenging for large datasets like the complete set of sequenced SARS-CoV-2 samples.

Our objective is to create a computationally efficient, tree-free approach to infer infection matrices directly from neutral allele frequency time series. We show that, with adequate data, it is possible to map entire networks of disease transmission between communities. By analyzing genomic SARS-CoV-2 data from England, obtained from the COVID-19 Genomics UK Consortium (COG-UK) (28), and data from the United States obtained from GISAID (29), we highlight differences across variants of concern, examine the statistical characteristics of the resulting transmission networks, explore the significance of long- and short-range connections, and assess their impact on the spread of new variants.

## Results

### The Basic Idea.

**Fig. 1.**
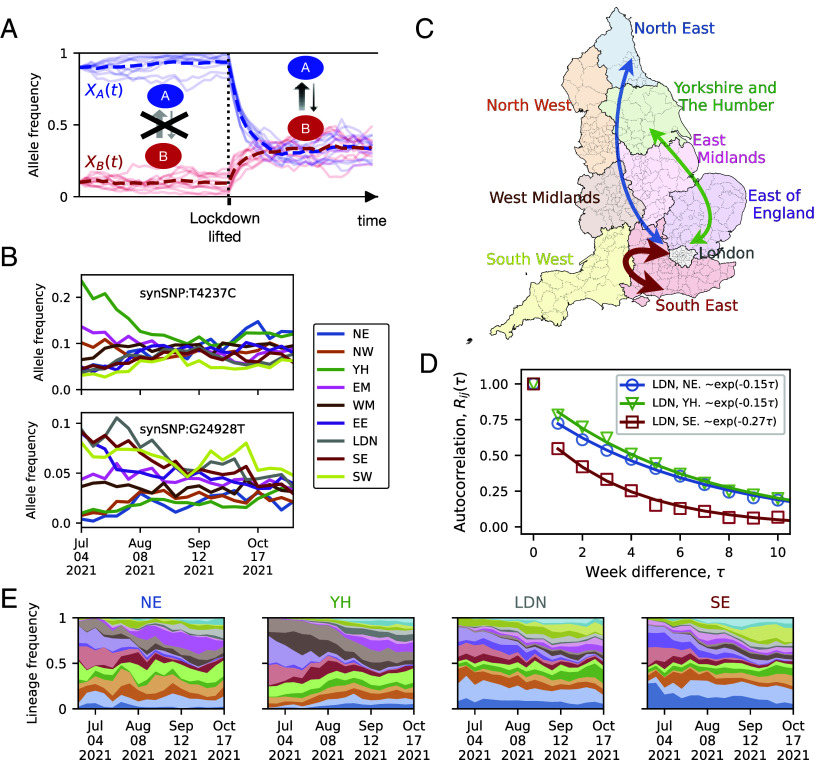
Intercommunity transmission promotes allele frequency convergence. (*A*) Simulated dynamics of a neutral variant to illustrate the effect of intercommunity disease transmission between two communities, A and B (see *SI Appendix*, section 2.4 for details of the simulation). Ten simulated trajectories are shown with their average as a dashed line. When the two communities are isolated by a traffic lockdown, their allele frequencies fluctuate independently due to genetic drift acting independently in both populations. After the lockdown is lifted (dotted vertical line), the allele frequencies tend to converge due to the exchange of infections between the communities. The rate of convergence reflects the rate of case importations. (*B*) Time series of allele frequencies for two prevalent SARS-CoV-2 alleles (*Top* and *Bottom* graphs), exemplifying allele frequency convergence during the Delta wave in England. Each color corresponds to one of the nine regions of England shown in (*C*). (*D*) Averaged over the 40 most abundant, approximately independent alleles in our dataset, the rate of convergence substantially differs for different region pairs. The markers show the autocorrelation function $R_{\mathrm{ij}}\left(\tau \right)$ of the allele frequency differences between regions *i* and *j*. We focus on the three region pairs indicated by arrows in (*C*): London and North East (blue); London and Yorkshire and the Humber (green); and London and South East (red) (see *SI Appendix*, section S.1 for the procedure for selecting the alleles over which we have averaged and for the mathematical definition of $R_{\mathrm{ij}}\left(\tau \right)$). The solid lines represent exponential fits. (*E*) The relative abundance of the top 20 Delta variant lineages over time in North East, Yorkshire and The Humber, London, and South East. Decay rates similar to those in (*D*) are obtained from lineage-frequency data (*SI Appendix*, Fig. S5).

We can illustrate the core of our method by examining how the importation of cases affects allele frequency differences between communities. Imagine two populations, A and B, that are initially epidemiologically isolated due to a complete traffic lockdown, which is later lifted. The population genetic simulations in Fig. 1*A* show that, during the isolation period, allele frequencies fluctuate independently in each population since there is no interaction between both populations. However, once the lockdown ends, both communities begin exchanging infections, causing their allele frequencies to gradually converge. The higher the transmission rate between the two populations, the faster this frequency alignment occurs. Our method leverages this phenomenon, computationally linking the convergence of allele frequencies to intercommunity transmission.

Before delving into our method, it is useful to first verify that the expected alignment of frequency trajectories is indeed observable in SARS-CoV-2 data. Fig. 1*B* demonstrates that allele frequency differences between regions in England, likely arising from strong stochastic drift during the early Delta wave, tend to converge over time (see *SI Appendix*, Fig. S24 for earlier time points). Moreover, Fig. 1*D* demonstrates that, on average, allele frequency mismatches between nearby regions, such as London and the South East, diminish faster than those between distant regions, like London and the North East, in line with an isolation-by-distance expectation (30) (see *SI Appendix*, Fig. S5 for additional comparisons between region pairs). The Muller plots in Fig. 1*E* illustrate how the sample frequencies of the top 20 Delta variant lineages in the COG-UK phylogenetic tree fluctuated across regions in England during the 2021 Delta wave (*SI Appendix*, section S.1).

### Overview of the Inference Approach.

To mathematize the above idea, we resort to the principles by which lineage frequencies evolve under neutrality.

Consider a population composed of *n* subpopulations, distinguished by location (different cities or districts), age, ethnicity, or any other features that might influence the epidemic characteristics of its members. Under neutrality, we can assume that, up to random fluctuations, the frequency $X_{i}\left(t\right)$ of a particular lineage in population *i* at time *t* depends linearly on the lineage frequencies ${\left\{X_{j}\left(\tau \right)\right\}}_{j=1\cdots n}$ at some earlier time *τ* < *t*,

[1] $$\begin{array}{c} X_{i}\left(t\right)=\sum_{j=1}^{n}A_{\mathrm{ij}}\left(t;\;\tau \right)X_{j}\left(\tau \right)+\;\text{noise}\;, \end{array}$$

where **A** is a right-stochastic *n* × *n* matrix: Its elements are nonnegative and, within each row, sum up to one, $\sum_{j}A_{\mathrm{ij}}=1$. For now, we do not need to know anything about the noise term, except that its expectation vanishes (See *Materials and Methods* for a derivation of Eq. **1**).

The coefficient $A_{\mathrm{ij}}$ represents the proportion of infections that population *i* imports from population *j*, thus capturing cross-infection rates between regions. Therefore, we refer to **A** as the importation-rate matrix. Yet, an equally important dual interpretation of the matrix **A** arises when one tries to model backward processes, where one starts from a given sampled genome and follows its lineage of ancestors backward in time. For this process, $A_{\mathrm{ij}}\left(t;\;\tau \right)$ describes the probability that the lineage jumps from population *i* to population *j* as time is run backward from *t* to *τ*. This backward-in-time interpretation is needed, for example, when one tries to embed phylogenetic trees within a metapopulation, because $A_{\mathrm{ij}}\left(t;\;\tau \right)$ provides the probabilistic weight for assigning the branch of a phylogenetic tree to a transition from population *i* to *j*. We will frequently return to the interpretation of the rows of **A** as backward transition probabilities, as it helps develop intuition and hypotheses for the structure of **A**. For example, the constraint $\sum_{j}A_{\mathrm{ij}}=1$ is obviously required for the interpretation of the rows of **A** as probability distributions.

The most straightforward way to estimate importation rates, $A_{\mathrm{ij}}$, using the model described above, is to minimize the least squares difference between predicted and observed lineage frequencies over all right stochastic matrices. This method is most effective when the true lineage frequencies are known; however, in practice, these frequencies are derived from a random sample of infected individuals. As a result, the observed frequencies are affected by sampling noise, often leading to an overestimation of importation rates, as demonstrated in *SI Appendix*, section S.2.3. This bias arises because, in the high-noise limit, the optimization tends to favor the uniform matrix, i.e., $A_{\mathrm{ij}}=\frac{1}{n}$.

**Fig. 2.**
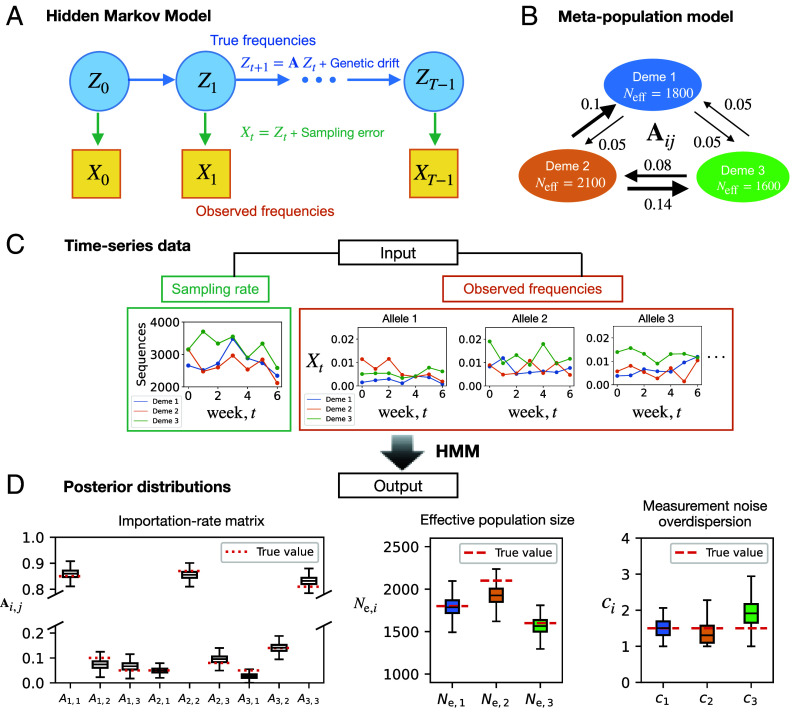
Inference method overview. We use a hidden Markov model (HMM) with continuous hidden and observed states [a Kalman filter (31)] to infer transmission networks from allele frequency time series. (*A*) A schematic of the HMM for the frequency dynamics. (*B*) To demonstrate the utility of our approach, we here use the depicted 3-deme model to generate allele frequency time series as input for the inference of the importation rates (arrows). In these simulations, the frequencies are evolved according to Eq. **4**, with their initial frequencies being the same as those observed in EE, LDN, and SE of England during the week of June 20–26, 2021. We used effective population sizes comparable to those measured for England regions during the Delta wave period (32). The measurement noise overdispersion parameter (defined in *Materials and Methods*) is set higher than the value actually inferred, for illustrative purposes. (*C*) The input for the inference comprises weekly sampled sequences (*Left*) and observed frequencies of lineages (*Right*) within a focal variant. (*D*) The output is posterior distributions of the 6 importation rates of the 3 × 3 network (*Left*), of the local inferred population size (*Middle*), and of the deviation from uniform sampling (*Right*). Each box shows the interquartile range (IQR), with the median indicated by a horizontal black line. The whiskers extend to the most extreme data points that fall within 1.5 times the IQR below the first quartile or above the third quartile. Red dotted lines indicate the true values.

To avoid sampling biases, we employ a hidden Markov model (HMM, sketched in Fig. 2*A*). The HMM analyzes the entire trajectory of the time series, treating true frequencies as hidden states and incorporating genetic drift and sampling error as Gaussian noise processes. Posterior distributions of the importation rates and the noise strengths are inferred using a Markov Chain Monte Carlo (MCMC) method. To speed up training, we have also implemented an expectation–maximization (EM) algorithm, which yields maximum likelihood estimates of the parameters. For tracking neutral lineages, mutations are used as a tree-free alternative, with clustering techniques employed to ensure neutrality and reduce statistical errors (33) (*SI Appendix*, section S.1.2.1). Further details about the inference method and the data processing are reported in *Materials and Methods* and *SI Appendix*, sections S.1 and S.2.

In Fig. 2*B*–*D*, we evaluate the effectiveness of our method to infer a known importation-rate matrix from synthetic data simulated under a metapopulation model of three demes interacting through a 3 × 3 matrix. The simulation duration (7 wk), number of lineages, and effective population size were chosen to reflect the conditions during the Delta plateau period (Aug 2021–Dec 2021) in England (32). We report unnormalized (absolute) values of $A_{\mathrm{ij}}$ because they directly reflect the magnitude of inferred importation rates between regions, making it easier to identify which links dominate the dynamics. Relative estimation errors **$\frac{|A_{\mathrm{ij}}^{\text{inferred}}-A_{\mathrm{ij}}^{\text{true}}|}{A_{\mathrm{ij}}^{\text{true}}}$** are generally larger for small importation rates; however, despite this large relative uncertainty, system-level quantities such as relaxation times can still be inferred with reasonable accuracy if the absolute errors remain small (*SI Appendix*, section S.2.5). See *SI Appendix*, section S.2.3 for a further demonstration of how the variance in the estimates depends on the amount of available data, and *SI Appendix*, section S.4 for how the spatial resolution of the data affects the inference.

### Application to SARS-CoV-2 in England.

To apply our method to real-world data, we first focus on England due to its large number of sequenced SARS-CoV-2 cases since the early stages of the pandemic (*SI Appendix*, Fig. S32). The fall of 2021 constitutes a particularly suitable test case because of its consistently high and relatively stable incidence numbers over more than four months (*SI Appendix*, Fig. S33). We initially concentrate on this plateau phase of the Delta wave because it offers long allele frequency time series data with relatively low statistical error. In later sections, we also apply our method to the Alpha and Omicron waves in England, as well as the Delta wave in the United States.

Following our observations of regional allele frequency fluctuations in England (Fig. 1*C*–*E*), we further subdivided each region to create a total of 50 subunits, which we call demes, to enhance the spatial resolution of our interaction networks. Our subdivision algorithm ensures that each deme contributes a roughly similar number of sequences (*SI Appendix*, section S.13.1). We provide evidence in *SI Appendix*, section S.4 that the inferred results are robust at this level of resolution, but become less reliable when the number of demes is further increased (*SI Appendix*, Figs. S16 and S17). This reflects a trade-off: While finer resolution improves spatial detail, it reduces the number of sequences per deme and increases the number of parameters to be inferred, which makes the parameter estimates less reliable.

Since sampling and incidence reporting typically follow a weekly cycle, we run our inference with a time step of one week (t−τ=1 week in Eq. **1**). This time step aligns well with the generation time of SARS-CoV-2, which has an average infectious period of approximately 4 to 6 d depending on the variant (34, 35).

### Transmission Networks Mirror Geography.

**Fig. 3.**
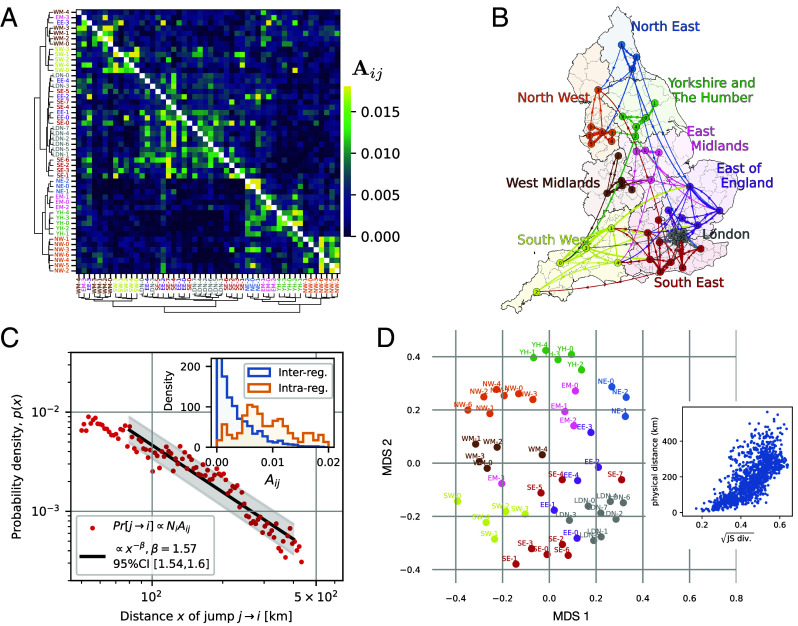
Network inference reveals heterogeneity, distance dependence, and stochasticity of disease transmission (England, Delta wave period, from Jun 20 to Sep 25, 2021). (*A*) Heat map of the matrix **A**. The matrix coefficient $A_{\mathrm{ij}}$ represents the proportion of infections that population *i* imports from population *j*, thus encoding cross-infection rates between regions. Hierarchical clustering was used to order the 50 demes according to the similarity between rows of the matrix, as quantified by the Jensen–Shannon divergence (*SI Appendix*, section S.7). (*B*) Illustration of the main infection pathways (the largest 5% of the off-diagonal matrix elements). (*C*) Inferred probability distribution of jump distances: A jump from deme *j* to deme *i* is assumed to occur with probability $\propto N_{i}A_{\mathrm{ij}}$, where *N*_*i*_ represents the population size of deme *i* (see *SI Appendix*, section S.7 for the underlying rationale). *Inset*: Histogram of matrix elements within and across regions. (*D*) Multidimensional scaling reveals an approximately geographic arrangement of populations on a two-dimensional plane. MDS is a procedure that projects high-dimensional data on a plane so as to maintain pair-wise distances as closely as possible. Here, pairwise distances are taken to be the square roots of Jensen–Shannon divergence between the rows of the matrix presented in (*A*). In the *Right*-side plot, the square root of Jensen–Shannon distance is compared with the physical distance (the Spearman correlation = 0.75).

After inferring the importation-rate matrix **A**, we performed hierarchical clustering to order the 50 demes by the similarity of their transmission networks, as quantified using the Jensen–Shannon divergence between their respective rows (*SI Appendix*, section S.8). Remarkably, the resulting matrix exhibits a pronounced block structure (Fig. 3*A*) with different blocks corresponding to geographically well-connected regions, such as the demes within London or near Liverpool (NW-2) and Manchester (NW-1). When we plot the major infection paths (the largest 5% of the off-diagonal matrix elements) on a map of England (Fig. 3*B*), a network of predominantly local connections becomes apparent. This aligns with a general decline of importation rates with distance and larger importation rates within than between regions (Fig. 3*C*).

To further explore how well epidemiological interactions mirror geographic relationships, we sought a two-dimensional representation of the entire network of importation rates. To this end, we performed a multidimensional scaling (MDS) analysis (36) to embed the locations of all the demes in a plane such that the in-plane distance between any two locations measures how different their vectors of importation rates are. The result, properly rotated, crudely resembles the map of England (Fig. 3*D*). See *SI Appendix*, section S.8 for details on the MDS and data processing analyses.

### Transmission Networks Are Asymmetric and Evolve.

**Fig. 4.**
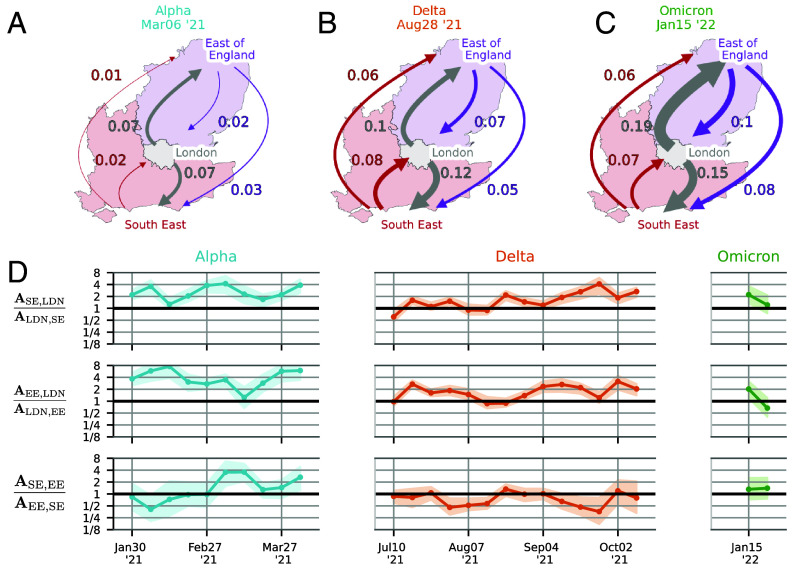
Time dependence of transmission networks. (*A*–*C*) The 3 × 3 importation-rate matrix between London and its two neighboring regions, East of England and South East of England, is inferred using the HMM-MCMC method. Arrows represent the mean importation rates for the Alpha, Delta, and Omicron variants. (*D*) Time series showing the ratio of importation rates between each pair of regions, with shaded areas indicating the *Upper* and *Lower* quartiles. At each timepoint, the importation-rate matrix is inferred using a time window of 7 wk centered around that timepoint.

The planar MDS representation only provides a time-averaged picture and also ignores any asymmetry in the epidemiological interactions among populations. To investigate the dynamics and asymmetry of infection, we focus on London (LDN) and its neighboring regions East of England (EE) and South East (SE). Applying our method to the Alpha, Delta, and Omicron waves results in the intercommunity transmission rate matrices illustrated in Fig. 4*A*–*C*. We find that, during the Alpha and Delta waves, London generally had a stronger impact on South East and East of England than vice versa. To formally assess the asymmetry during each wave, we averaged log-ratios $\ln\frac{A_{\mathrm{ij}}}{A_{\mathrm{ji}}}$ over time points (Fig. 4*D*) and identified pairs (i,j) with $\frac{A_{\mathrm{ij}}}{A_{\mathrm{ji}}}>1$ with high posterior probability while keeping the family-wise posterior error ≤0.05 (ref. 37 and *SI Appendix*, section S.11.1). The identified pairs are $\frac{A_{\text{EE},\;\text{LDN}}}{A_{\text{LDN},\;\text{EE}}}\in \left[2.11,6.89\right]$ (95% credible intervals, CrIs) and $\frac{A_{\text{SE},\;\text{LDN}}}{A_{\text{LDN},\;\text{SE}}}\in \left[1.65,3.94\right]$ for the Alpha variant, while $\frac{A_{\text{EE},\;\text{LDN}}}{A_{\text{LDN},\;\text{EE}}}\in \left[1.24,2.19\right]$ and $\frac{A_{\text{SE},\;\text{LDN}}}{A_{\text{LDN},\;\text{SE}}}\in \left[1.21,2.09\right]$ for the Delta variant. Interestingly, within waves, we detect only relatively modest shifts (Fig. 4*D*), such as the growing relative influence of London on South East from July to October during the Delta wave (*SI Appendix*, section S.11.2 for a Bayesian test of this claim).

### Inference Indicates Pronounced Genetic Drift and Uniform Sampling Noise.

**Fig. 5.**
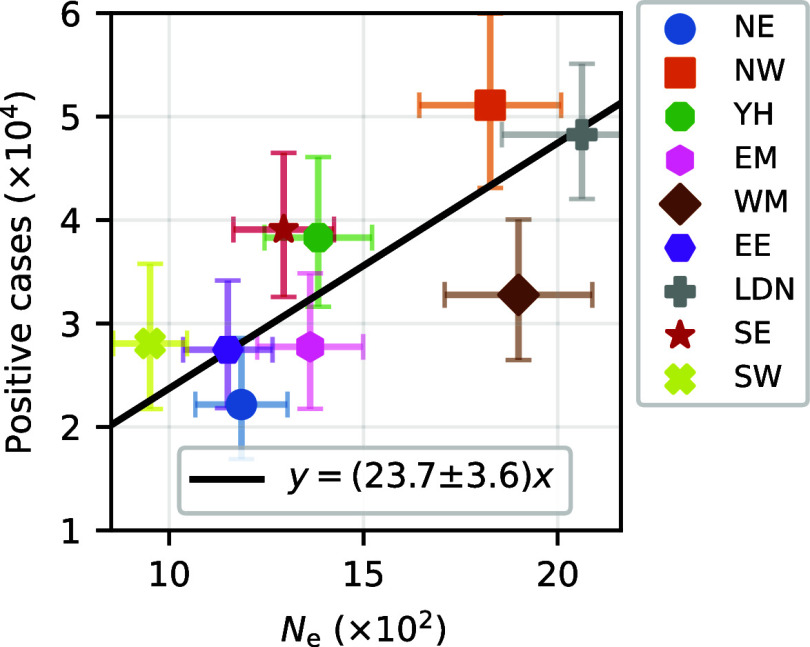
Stochasticity of disease transmission, quantified by the effective population size. For the Delta wave in England, the inferred effective population size at the region level (x-axis) is compared to the estimated number of infected individuals (y-axis). The Pearson’s correlation is 0.73 (*P*-value = 0.026). The error bars represent the 95% CI, and the solid line indicates the best linear fit.

Our HMM framework not only estimates importation rates but also quantifies the strength of genetic drift via the effective population size, $N_{e,i}$, and assesses the impact of sampling noise through the overdispersion parameter, *c*_*i*_. We compared the inferred effective population size with the number of infected individuals estimated by the COVID-19 Infection Survey (38). While there is a strong correlation between the two, the inferred effective population size was consistently lower by factors ranging from 18 to 29 across regions (Fig. 5). This discrepancy between effective and infected population sizes, which aligns with earlier studies aggregating over all England (32), may be explained by factors such as superspreading events and community structure (32, 39–44), as well as potential mutational fitness effects, although significantly nonneutral alleles were removed prior to the inference. In contrast, the overdispersion parameter *c*_*i*_ was close to one (*SI Appendix*, Fig. S25), indicating that sampling error is uniform and of expected magnitude in the COVID-19 Infection Survey. Thus, our inference reveals substantial genetic drift relative to the number of infections, while indicating that sampling noise was consistently within expected bounds across regions.

### Transmission Networks in the US Mirror Geography and Reveal Greater Importance of Long-Range Interactions than Expected from Mobility Data.

**Fig. 6.**
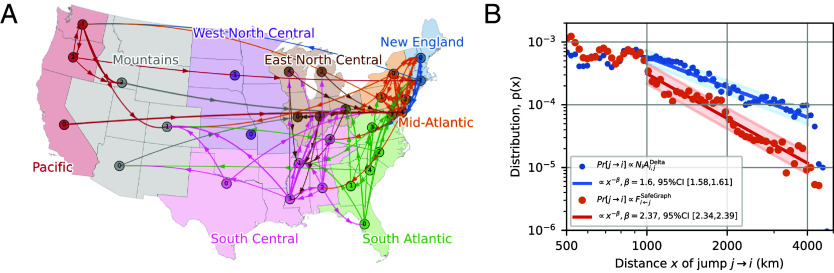
Transmission network in the United States during the Delta wave (30 demes, Jul 18, 2021–Oct 30, 2021) and comparison to mobility data. (*A*) Illustration of the main infection pathways. Arrows indicate the largest 5% of the off-diagonal matrix elements $A_{\mathrm{ij}}$. See *SI Appendix*, Fig. S30 for the inferred matrix. (*B*) Comparison between the jump-size distribution inferred from the 30 × 30 importation-rate matrix (blue) and indirectly estimated using the SafeGraph data (orange).

For comparison, we also applied our method to the United States, focusing on the Delta wave (Jul 18–Oct 30, 2021). We divided the United States into 30 subunits using the same subdivision algorithm employed in the analysis of England (*SI Appendix*, section S.13.2). Fig. 6*A* illustrates major importation pathways. Similar to England, we found that intercommunity importation rates in the US mirror geographic relationships (*SI Appendix*, Fig. S31).

The US data also allow us to compare the jump-size distribution with what would be expected based solely on mobility data collected by SafeGraph during the period of January to May 2020 (45). SafeGraph gathers data from a large panel of anonymous mobile devices in the United States, recording an average “home” location over six weeks and all locations where the device pauses for at least one minute (see *SI Appendix*, section S.1.3 for data processing). To convert these data to the lineage jump probability, we assume that a lineage jumps from deme *j* to deme *i* with a probability proportional to $F_{i\leftarrow j}$, the rate of trips from deme *j* to deme *i*.

Our analysis shows that the jump-size distribution directly inferred from the sequencing data decays substantially slower with distance than predicted by the mobility proxy data (Fig. 6*B*). This suggests that deducing epidemiological interactions from mobility data may require a different model or different information than the aggregated cell phone movements provided by SafeGraph.

We note that while the scaling exponent of the jump-size distribution for the US data is similar to that for the England data, the MDS reconstruction (*SI Appendix*, Fig. S31) for the United States is less geographically faithful; the Spearman correlation between geographic distance and the square root of Jensen–Shannon distance is 0.75 for the England result, whereas it is 0.52 for the US result. One possible explanation is the substantial variation in the geographic sizes of demes in the US analysis (e.g., California vs. Rhode Island), compared to the more uniform spatial units used in the England analysis. Treating a geographically large state as a single-point deme (located at its centroid) may obscure finer-scale mobility patterns, weakening the geographic signal in the importation rates.

### Timescale Decomposition via Eigenvalue Analysis.

Given the potentially large number of inferred importation rates—2,500 in the case of England—it is important to determine which aspects of the transmission network are truly relevant for predicting epidemiological trajectories. Intuitively, what is relevant should depend on the timescale of interest. For instance, if we are concerned with the dynamics from one viral generation to the next, we need a detailed transmission network that captures the strongest importation rates, such as those within a city. Conversely, if we are interested in the dynamics over several weeks, the fine structure is less critical. After a brief relaxation period, during which spatial differences smooth out due to mixing, the different parts of a city with strong intracity connections will fluctuate coherently, allowing cities to be effectively represented as single compartments with weaker intercity links.

Our dynamical model of neutral frequencies enables us to formalize the intuitive concept of timescale-dependent collective variations. First, note from Eq. **1** that our best prediction for the allele frequencies at future time *t* is given by the $t^{\text{th}}$ power of the matrix **A** applied to the current allele frequencies,

$$E\left[X(t)\right]=A^{t}\;X\left(0\right).$$

To compute the matrix power on the right-hand side, it is natural to decompose the matrix **A** in terms of its eigenvalues, upon which we get

$$E\left[X(t)\right]=\sum_{\mu =0}^{n-1}a_{\mu }\lambda_{\mu }^{t}v_{\mu },$$

where $v_{\mu }$ is the right eigenvector of **A** corresponding to the eigenvalue *λ*_*μ*_. Each term in this sum describes the time evolution of a spatial variation aligned with $v_{\mu }$, with its amplitude scaled by $\lambda_{\mu }^{t}$. The mode amplitudes *a*_*μ*_ are determined by the inner product $\left\langle u_{\mu }|X\left(0\right)\right\rangle$ between the $\mu^{\text{th}}$ left eigenvector $u_{\mu }$ and the initial frequency vector X(0). We assume that the eigenvalues are arranged in descending order of magnitude and that the eigenvectors are normalized such that $\left\langle u_{\mu }|v_{\mu^{\prime }}\right\rangle =\delta_{\mu ,\mu^{\prime }}$.

We expect the dynamics to mix all demes over long times, unless there are isolated pockets, leading to an eventual equalization of the expected allele frequencies across demes. This outcome is guaranteed i) if **A** has an eigenvalue $\lambda_{0}=1$ corresponding to a right eigenvector $X\propto {\left(1,\ldots ,1\right)}^{⊤}$, representing equal allele frequencies in all demes, and ii) if the magnitudes of all other eigenvalues are less than 1, $|\lambda_{\mu >0}|<1$. These conditions are mathematically ensured by the right-stochasticity of **A**, provided that it is also irreducible.

### Relaxation Dynamics and the Critical Role of Long-Distance Connections.

**Fig. 7.**
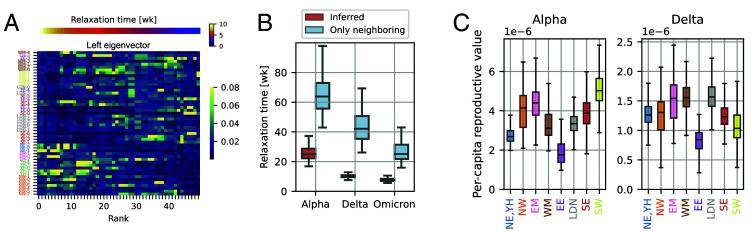
Relaxation dynamics and reproductive values. (*A*) The absolute values of the left eigenvectors $v_{n}$ of the 50×50 matrix during the Delta wave in England, presented in Fig. 3*A*. Each eigenvector $v_{n}$ is normalized such that $\sum_{i}\left|{\left(v_{n}\right)}_{i}\right|=1$. (*B*) The longest epidemiological relaxation times [week] is calculated for each major variant in England from the regional importation-rate matrix. For comparison, we also show the relaxation times obtained from a modified matrix where transmission between nonneighboring regions is excluded. (*C*) The per-capita reproductive value for the Alpha and Delta variants in England. Here, the reproductive value*π*_*i*_ is evaluated by inferring the importation-rate matrix **A** at the region level, where NE, which has fewer sequences, is combined with YH to obtain reliable results. The values of *π*_*i*_ moderately depend on the choice of time intervals used for the inference (*SI Appendix*, Fig. S26). In the boxplots, the boxes represent the interquartile range (IQR), with the central line indicating the median. The whiskers extend to the most extreme data points within 1.5 times the IQR from the first and third quartiles.

Before discussing the long-term stationary state, we first address the relaxation toward stationarity. The amplitude of mode *μ* decays e-fold after a time $\tau_{\mu }\equiv -1/\ln\left|\lambda_{\mu }\right|$, which diverges as $|\lambda_{\mu }|$ approaches unity. Generally, many eigenmodes influence the relaxation process, as shown in Fig. 7*A* for the Delta wave in England. However, the total time to reach the steady state is controlled by the longest relaxation time *τ*_1_.

In Fig. 7*B*, we display *τ*_1_ for the Alpha, Delta, and Omicron waves in England. The relaxation times differ significantly between waves; for instance, the relaxation time during the Delta wave is approximately 10 wk, which is less than half of the relaxation time during the Alpha wave.

What aspects of the transmission network influence the overall relaxation time? While the strongest interactions-typically short-range transmissions between neighboring demes-undoubtedly play an important role, the contribution of relatively weak long-distance connections is less obvious. To evaluate their effect, we artificially removed all inferred long-distance connections (those spanning nonneighboring regions) and reevaluated the relaxation spectrum. This intervention led to a substantial increase in the slowest relaxation time *τ*_1_—by more than a factor of four during the Delta wave, for example. As a complementary analysis, we also evaluated the statistical significance of nonneighboring transmissions by assessing their impact on the HMM likelihood (*SI Appendix*, section S.9). The Alpha variant provided modest, nonsignificant evidence (*P* = 0.092), possibly due to lower sequence reporting and a relaxation time (∼26 wk) exceeding the 14-wk inference window. For the Delta variant, allowing nonneighboring connections substantially improved the model fit ($P=6.0\times 10^{-4}$). These findings indicate that even rare long-distance transmission events can meaningfully shape the dynamics of disease spread and must be accounted for to accurately predict pandemic timescales.

### Equilibration and Reproductive Value.

To illuminate the long-term stationary state, let us consider a neutral allele initially fixed in deme *i* and absent in all other demes. Due to case importations, allele frequencies are expected to gradually even out across demes. Consequently, the long-term expected frequency, *π*_*i*_, is the same in all demes and depends only on the identity *i* of the initial deme. The vector $\pi ={\left(\pi_{1},\cdots ,\pi_{n}\right)}^{⊤}$, composed of these primary-deme-dependent long-term frequencies, is proportional to the leading left eigenvector previously denoted by $u_{0}$ and normalized to sum to 1, $\sum_{i}\pi_{i}=1$. *π*_*i*_ represents the expected contribution of deme *i* to the future gene pool, which is called the class reproductive value (46, 47).

When we normalize the reproductive value of a deme *i* by its number *I*_*i*_ of infected individuals, as measured by the COVID-19 Infection Survey (38), we obtain the per-capita reproductive value $\pi_{i}/I_{i}$, which is the probability that a viral genome picked at random from an infected individual in the distant future traces its ancestry back to this individual in the present generation (46, 47). If the infection dynamics were the same everywhere in England, one would expect per-capita reproductive values to be the same across all regions. Interestingly, the posterior means of per-capita reproductive values vary by about a factor of 2 across regions in both the Alpha and Delta waves (Fig. 7*C*). For example, during the Alpha wave, SW exceeds EE by a factor of 2.97, [1.44, 4.54] (median, 95% CrI), and during the Delta wave LDN exceeds EE by a factor of 2.04, [1.16, 4.32]. See *SI Appendix*, section S.11.3 for other pairwise ratios identified by a Bayesian multiple-comparison analysis.

In the same dataset, we find that ratios of reproductive values approximately predict ratios of case importation rates (*SI Appendix*, sections S.3 and S.5),

[2] $$\begin{array}{c} \frac{\pi_{j}}{\pi_{i}}\approx \frac{A_{\mathrm{ij}}}{A_{\mathrm{ji}}}. \end{array}$$

This relation, with an equal sign, is called detailed balance. Most epidemiological models impose detailed balance to avoid cyclic dynamics in the equilibrium behavior of the lineage jump process-patterns that are generally viewed as unrealistic. Thus, observing detailed balance supports the validity of fundamental assumptions commonly made in epidemiological modeling.

Additionally, detailed balance, Eq. **2**, helps relate heterogeneities in reproductive value to heterogeneities in transmission coefficients. For example, a lower per-capita reproductive value for East of England (EE) is observed both during the Alpha and Delta waves in England (Fig. 7*C*, *Right*), suggesting that the per-capita importation rate from EE to any other region is systematically lower than the other way around. In *SI Appendix*, section S.3, we further investigate these importation-rate patterns, demonstrating that the heterogeneities suggested by the reproductive values can be confirmed directly from the importation-rate matrix. We also argue that possible variations in reporting timing could affect heterogeneous patterns of *π*_*i*_, highlighting the importance of accurate reporting dates for precise inference (*SI Appendix*, Fig. S14). In summary, reproductive values capture spatial heterogeneity in long-term viral contributions, which, via detailed balance, mirrors heterogeneity in importation rates.

### Selected Variants.

We have previously emphasized that inferring $A_{\mathrm{ij}}$ sheds light on case importation patterns, their variations across time and space, their potential for control, and their role in embedding phylogenetic trees. But knowing the $A_{\mathrm{ij}}$ of a variant is useful also because it enables predicting its behavior if it is under selection. In *SI Appendix*, section S.6, we show how selective forces modify the allele frequency dynamics in an SIR or SEIR model. In particular, when a variant under positive selection is rare, its dynamics follows the neutral dynamics in Eq. **4**, with the modification $A_{\mathrm{ii}}\to A_{\mathrm{ii}}+\sigma$, where *σ* is the selected advantage of the considered variant. Thus, as long as the variant is rare, its expected frequency is computed as $E\left[X(t)|X(0)\right]=e^{\sigma t}A^{t}X\left(0\right)$.

**Fig. 8.**
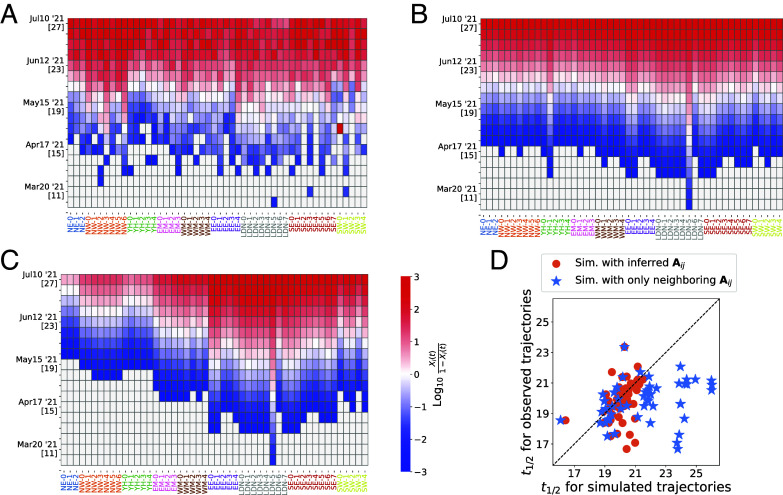
Replaying the dynamics of a selective sweep. (*A*) Observed frequencies of the Delta variant in England, ranging between $10^{-3}$ and 1 to 10^−3^, across $N_{D}=50$ locations in England. The heatmap displays the frequencies on a logit scale. On the vertical axis, epidemiological weeks (epiweeks) in 2021 are indicated below the dates by [·]. (*B*) Simulations following the nonneutral transmission model described in *SI Appendix*, section S.10, with intercommunity importation rates as inferred for the Delta wave (shown in Fig. 3). The observed frequencies at epiweek 10, when the Delta variant was reported only in deme “LDN-5-5,” are used as the initial condition (see *SI Appendix*, section S.10 for the fitting process). (*C*) Simulated frequencies, where transmission between nonneighboring regions (such as London and North West) is switched off. See Supplementary Movie for the comparison between (*A*–*C*). (*D*) Comparison of the midpoint epiweek $t_{\frac{1}{2}}$—the week when the frequency in a deme reaches 0.5—between observed data and simulated data for each deme. Orange circles represent $t_{\frac{1}{2}}$ from the simulation with the inferred $A_{\mathrm{ij}}$, while blue stars represent $t_{\frac{1}{2}}$ from the simulation without transmission between nonneighboring regions. The horizontal bars indicate the SDs obtained from the bootstrapping method.

The kymograph in Fig. 8*A* illustrates the spreading pattern of the Delta variant across England. We conducted simulations of the Delta variant’s frequency dynamics (detailed in *SI Appendix*, section S.10), starting from frequencies observed in the week of March 7–13, 2021, when Delta variant sequences were reported solely from the deme labeled “LDN-5.” Fig. 8*B* displays the simulated abundance of the Delta variant using the inferred $A_{\mathrm{ij}}$, presented in Fig. 3*A*, along with fit parameters associated with the relative infectivity of the Delta variant (*SI Appendix*, section S.10). To assess the impact of long-range transmission, we artificially removed the inferred connections between nonneighboring regions and then conducted the same simulation (Fig. 8*C*).

Fig. 8*D* shows the comparison of the midpoint time, $t_{1/2}$, at which the abundance of the Delta variant reached 50% in a deme, between the observed and simulated data. The simulation using the inferred matrix roughly reproduces observed $t_{1/2}$, clustering around epiweeks 18 to 21. In contrast, without transmission between nonneighboring regions, the simulation shows delays in $t_{1/2}$. In particular, for the NE, NW, and YH demes, which are distant from the initial seeding at “LDN-5,” the $t_{1/2}$ values are predicted to be delayed by 2 to 6 wk without the nonneighboring transmission. These analyses show that the inferred importation rates $A_{\mathrm{ij}}$ capture the Delta variant’s spatial spread, highlighting the significant role of long-range transmission in accelerating its spread.

## Discussion

The infection rates between individuals from different population segments are among the many known unknowns of a pandemic. These rates, crucial for forecasting pathogenic spread, depend on complex behavioral patterns, immunity levels, and nonpharmaceutical interventions, rendering them exceptionally challenging to measure directly.

We here proposed an approach to infer these elusive rates retrospectively by examining the dynamics of viral genetic variation among different population groups. While our method could, in principle, be applied to various forms of population structure—including age groups or social groups—this study focuses specifically on geographically defined demes. Utilizing this method, we constructed transmission networks during the COVID-19 pandemic in England and the United States. Our findings shed light on the heterogeneity and plasticity of transmission networks, which have implications for epidemiological forecasting and intervention strategies.

### Methodological Advances.

Our approach hinges on the premise that the observed frequencies of neutral variants are influenced solely by intercommunity transmission, random genetic drift, and observational noise. By averaging out these noises, we can isolate the impact of cross-transmission, which tends to reduce regional differences in allele frequencies. This convergence toward a common value is evident in the allele frequency data of SARS-CoV-2 (Fig. 1) and demonstrates a plausible distance dependence. To optimally capture this signal, we have implemented a Kalman filter, which enables us to infer importation rates, local effective population sizes, and the strength of observational noise. A key advantage of this method is that it only relies on allele frequency time series, bypassing the need to construct a phylogenetic tree, which can be computationally demanding, especially when dealing with polytomies. We validated our method using population genetics simulations of a metapopulation with parameters consistent with those of SARS-CoV-2.

Compared to existing approaches, our method is unique in i) performing tree-free inference, and ii) quantitatively estimating directional importation rates between populations. Tree-based methods such as MASCOT (48) and TransPhylo (49) require a timed phylogeny as input, which is limited by the need for reliable phylogenetic reconstruction. The enrichment-based method of Tran-Kiem et al. (50) is tree-free but focuses on short-range dynamics, using only pairs of nearly identical sequences and discarding most genetic variation. Unlike our approach, it produces relative enrichment scores rather than absolute transmission rates.

### Epidemiological Insights.

Our key finding is that, applied to highly sequenced SARS-CoV-2 populations, this method allows us to map an entire interaction network between different populations. Unlike methods based on proxies, this approach quantifies direct epidemiological interactions that can be integrated into models of the disease spread. Our findings reveal substantial heterogeneity and plasticity in disease transmission networks. By applying our method to the SARS-CoV-2 transmission data in England and the United States, we uncovered several key patterns:

#### Geographical mirroring and long-range interactions.

The inferred transmission networks largely reflect geographical proximities. Cross-importation rates are strongest between neighboring regions and gradually weaken with increasing physical distance. Interestingly, when interaction strength is converted into a distance and a map is drawn based on this metric, it roughly represents the geographic layout of England. This suggests that interactions reflect geographic relationships.

The spatial spread of new mutants is influenced by the underlying jump distance distribution of individual movements. In ref. 51, it was theoretically shown that the power-law exponent of the jump distance distribution controls the asymptotic growth behavior of the area seeded by a new mutant. Our results indicate that for both the England Delta variant and the US Delta variant, the observed jump distance distributions are heavy-tailed (corresponding to the case of *μ* < 0 in the notation of ref. 51), suggesting that the area affected by a new mutant will grow exponentially.

While the observed decay of interactions with distance likely reflects the limited traffic flow between distant regions, this decay is lower than expected based on traffic flows alone, as estimated from SafeGraph data. We see two potential reasons for the discrepancy: i) SafeGraph data treats all visited locations equally, regardless of the duration of the visit, which could underestimate the impact of long-distance visits. ii) Long-distance trips may be associated with riskier behaviors, thereby increasing the likelihood of disease transmission. Therefore, the reduction in mobility flux with distance may be partially offset by higher infection risk from long-distance travelers. To separate these contributions, differently curated mobility data will be required.

#### Dynamic changes in transmission patterns.

The cross-community importation rates and their directionalities exhibit considerable variation across different waves of variants of concern. These dynamics were evident in the varying importation rates between London, East of England, and South East during different waves, with noticeable shifts in transmission dominance between these regions. Such dynamic changes emphasize the need for adaptive modeling approaches that can accommodate evolving transmission patterns over time.

#### Asymmetry in cross-importation rates.

Our analysis showed that cross-importation rates are often heterogeneous, indicating that certain regions exert a stronger influence on others. In general, epidemiological “rock-paper-scissor” interactions could exist between different regions. However, we found that such nontransitive interactions are not observed in our inferred importation rates (*SI Appendix*, Fig. S18), which approximately satisfy a certain symmetry property (detailed balance) that ensures that the lineage dynamics backward is time reversible (no cyclic fluxes). This also allows using per-capita reproductive values (Fig. 7*C*) to compare the infectivity across regions.

### Implications for Epidemic Forecasting and Future Directions.

The ability to directly infer detailed transmission networks has the potential to improve epidemic forecasting. We found that weak long-range interactions are crucial for explaining the spreading of beneficial mutations. This highlights the importance of accounting for such interactions in epidemiological models, especially for highly transmissible pathogens like SARS-CoV-2.

However, since importation-rate matrices were also found to change considerably between waves, continuous genomic surveillance will be needed to update cross-importation rates. With accumulating time series data for different waves and different pathogens, future studies might also learn to predict the evolution of cross-importation rates through time. Such progress might come either by identifying interpretable patterns with the help of mechanistic epidemiological models or through machine learning.

The patterns we have identified show that detailed balance is a reasonable assumption for epidemic models but that different regions have different infectivities. Given detailed balance, one important open challenge is to understand mechanistically what sets the different infectivities of the different regions. Plausible candidate reasons are historical contingencies due to differential exposure to prior waves, inducing behavioral and immunological differences, or policy differences.

Understanding the detailed structure of transmission networks may also allow for the design of targeted interventions. For example, identifying regions with high cross-importation rates can help prioritize areas for vaccination, testing, and other control measures.

### Limitations and Assumptions of the Method.

While our allele frequency–based method offers substantial advancements, several limitations warrant discussion.

Our method assumes that the alleles we track are neutral. Including advantageous alleles that are spatially sweeping can lead to an overestimation of cross-importation rates from the origin to target areas. Therefore, it is crucial to exclude alleles whose selective changes are stronger than genetic drift over the considered time period, as we have done following our precursor work in ref. 32.

We inferred importation rates under the assumption that foreign introductions were negligible during the periods analyzed. This assumption is justified by our focus on the plateau phases of variant spread, during which domestic transmission overwhelmingly dominates foreign importations. While foreign introductions may have a nonnegligible impact during the early seeding of a variant, their contribution becomes minor once the variant is well established (27).

The absence of direct measurements for cross-importation rates means we lack a definitive benchmark for comparing our results. As a result, our inferred matrices should be interpreted with some caution. Nonetheless, the observed distance decay in our inferred intercommunity transmission networks is a convincingly realistic feature, aligning with expected cross-importation rates. It is reassuring that our method infers a plausible tradeoff between transmission rate and geographic distance based solely on frequency correlations, without requiring geographical information.

Although our framework robustly recovers the overall structure of the importation matrix, the degree of uncertainty varies systematically with interaction strength and spatial range. Strong interactions are consistently inferred across bootstrap replicates (*SI Appendix*, Fig. S29) and tend to be short-ranged, reflecting frequent, well-sampled transmission between nearby subpopulations. In contrast, weak interactions are inherently more uncertain, both because they contribute little to short-term equilibration and because their signal in the data is faint. Most weak short-range couplings therefore have limited dynamical relevance. The notable exceptions are weak but long-range connections: While their individual identities vary across inference runs, their collective presence is essential for reproducing the slowest relaxation modes and the overall mixing times of the system. Simulations with and without these interactions confirm their importance in facilitating large-scale epidemic spread, even though their precise identity remains uncertain. Accurately constraining such long-range links requires long observation windows that capture rare interpopulation transmission events-conditions met for the Delta variant but not for Alpha. This difference possibly explains why removing all long-range connections markedly reduces the likelihood of the data for Delta, but not for Alpha. The uncertainty in the identity of these crucial weak links complicates targeted interventions but has little impact on the predicted spreading timescales. Future work may develop better representations for weak but important connections, for instance through systematic coarse-graining or other regularization approaches.

Our method does not require constructing a genealogical tree; it only relies on standing genetic variation monitored over time. The accuracy of our inferences improves with the amount of time series data available. Therefore, our method is most effective when applied to periods where a particular variant of concern is already prevalent. However, it is less effective in measuring cross-importation rates early on when a variant is just beginning to invade and few samples are available. Phylogeographical inference methods are better suited for source attribution, as they enable tracing the emergence of a new variant (26, 27). Nonetheless, our analysis indicated that cross-importation rates did not change significantly within waves, suggesting that our inferences may serve as a baseline for cross-importation rates in the early stages of a new wave.

## Materials and Methods

### Inference Methods.

The simplest way to infer the cross-importation rates $A_{\mathrm{ij}}\left(t+\Delta t;\;t\right)$ in Eq. **1** for a fixed time difference Δt from time series data is to minimize the least square difference between the predicted and actual lineage frequencies,

[3] $$\begin{array}{c} A*\;=\;\underset{A}{\text{argmin}}\sum_{\begin{array}{c} i,\mu \\ |t^{\prime }-t|\leq T \end{array}}{\left[X_{i}^{\mu }\left(t^{\prime }+\text{Δ}t\right)-\sum_{j}A_{ij}X_{j}^{\mu }\left(t^{\prime }\right)\right]}^{2}, \end{array}$$

over all right stochastic *n* × *n* matrices, satisfying $A_{\mathrm{ij}}>0$, $\sum_{j}A_{\mathrm{ij}}=1$. The summation over time points $t^{\prime }$ on the right-hand side serves as a regularization step, which ensures that **A** does not vary on time spans smaller than 2T. The more sequencing data are available, the smaller *T* can be chosen, which leads to a better resolution of the temporal variations in **A**. Standard errors of the matrix coefficients are obtained by bootstrapping over the available lineages *μ*.

The linear regression approach in Eq. **3** is computationally efficient but requires as input the true lineage frequencies, which are never known exactly. Instead, one can only measure the frequencies within the sequenced sample, which represent the true frequencies distorted by sampling noise.

Accordingly, we have adopted an HMM, as depicted in Fig. 2, which treats the frequencies as hidden states. By modeling genetic drift and sampling noise as Gaussian distributions, the HMM effectively transforms into a computationally efficient Kalman filter (31) with a likelihood function that can be calculated analytically. The per-generation variance due to genetic drift is inversely proportional to the effective population size $N_{e}$, which is usually smaller than the actual number of infected individuals. Specifically, for SARS-CoV-2 in England, the ratio between actual and effective population size was found to range from tens to hundreds (32). Likewise, sampling noise can be larger than expected based on random sampling, if sampling is not random but entails correlations. We therefore set the sampling variance to be inversely proportional to $\frac{S_{i,t}}{c_{i}}$, where $S_{i,t}$ is the number of sequences sampled from population *i* at time *t*, and *c*_*i*_ measures the deviation from random sampling. To infer the strength of genetic drift (*N*_*e*_) and sampling noise (*c*_*i*_) from our HMM, we have implemented an MCMC algorithm, which yields posterior distributions for these parameters (*SI Appendix*, section S.2).

Our simulation results in *SI Appendix*, Fig. S9 show that, while both the least squares method and the HMM method with the MCMC algorithm retrieve importation-rate matrices close to the ground truth, the least squares estimation tends to overestimate small interactions. Such a bias appears from the fact that the solution of Eq. **3** tends toward the uniform matrix when noise levels are high.

We thus focused on the HMM method to minimize these biases. To reduce computational cost of the MCMC, we have also implemented an EM algorithm, which provides the maximum likelihood estimate of all relevant parameters. The inference error of the EM algorithm was assessed by using a bootstrapping approach, where the parameters were inferred multiple times from randomly created sets of alleles, each set maintaining the same size as the original set. An overview of our inference pipeline is presented in *SI Appendix*, Fig. S6.

### Neutral Evolution in a Metapopulation.

Here, we provide additional mathematical rationale for why Eq. **1** describes the frequency dynamics of a neutral allele in a metapopulation. First, the deterministic terms in Eq. **1** are linear in the frequencies because, under neutrality, the frequency of any union of lineages must obey the same stochastic evolution equation. Additionally, neutrality implies that frequencies do not change in expectation if the frequency is the same in all populations, necessitating that each row of **A** sums up to 1. (If $X_{i}\left(\tau \right)=x$ for all *i*, Eq. **1** implies $E\left[X_{i}\left(t\right)|X\left(\tau \right)\right]=x\sum_{j}A_{\mathrm{ij}}$, which equals *x* only if $\sum_{j}A_{\mathrm{ij}}=1$.) Finally, negative matrix elements are excluded because they can generate negative frequencies.

Note that an alternative way of writing Eq. **1** is

[4] $$\begin{array}{c} X_{i}\left(t\right)-X_{i}\left(\tau \right)=\sum_{j=1}^{n}A_{\mathrm{ij}}\left(t;\;\tau \right)\left[X_{j}\left(\tau \right)-X_{i}\left(\tau \right)\right]+\;\text{noise}, \end{array}$$

which explicitly shows i) that the frequency in *j* only influences the frequency in *i* if $X_{i}\neq X_{j}$, and ii) that a larger value of $A_{\mathrm{ij}}>0$ leads to a faster convergence of *X*_*i*_ to *X*_*j*_.

Each coefficient $A_{\mathrm{ij}}\left(t;\;\tau \right)$ for *j* ≠ *i* denotes the proportion of infections that population *i* receives from population *j* during the interval from *τ* to *t*. Meanwhile, $A_{\mathrm{ii}}\left(t;\;\tau \right)=1-\sum_{j\neq i}A_{\mathrm{ij}}\left(t;\;\tau \right)$ represents the proportion of infections that are not imported, i.e., “homegrown” infections.

From this perspective, it is straightforward to see how Eq. **4** arises. During the period from *τ* to *t*, population *i* imports a total of $I_{i}\left(t\right)A_{\mathrm{ij}}\left(t;\;\tau \right)$ infections from population *j*, with a fraction $X_{j}\left(\tau \right)$ of these infections carrying the focal allele. Thus, at time *t*, the expected total number of infections carrying the focal allele in population *i* is $I_{i}\left(t\right)\sum_{j}A_{\mathrm{ij}}\left(t;\;\tau \right)X_{j}\left(\tau \right)$. Consequently, the updated allele frequency X(t) is given by $\sum_{j}A_{\mathrm{ij}}\left(t;\;\tau \right)X_{j}\left(\tau \right)$. By substituting $A_{\mathrm{ii}}\left(t;\;\tau \right)=1-\sum_{j\neq i}A_{\mathrm{ij}}\left(t;\;\tau \right)$, we obtain Eq. **4**.

## Supplementary Material

Appendix 01 (PDF)

Movie S1. The observed frequencies of the Delta variant in England (from March 7, 2021, to July 10, 2021), along with simulated frequencies based on the inferred importation-rate matrix and a matrix excluding long-range importations (i.e., a matrix with zero entries for couplings across non-neighboring regions), are displayed on a geographical map of England. For each UTLA, the frequency of the Delta variant in the deme containing the UTLA is shown. The colors represent the logit of the frequency, log_10_ $\frac{f_{i}(t)}{1\mp f_{i}(t)}$, where *f*_*i*_(*t*) denotes the frequency of the Delta variant in deme *i* at time *t*.

## Data Availability

Some study data are available (The Python scripts for the HMM-EM method and the C++ code for the HMM-MCMC method, along with the Python scripts to reproduce the figures in this manuscript, are available at https://github.com/Hallatscheklab/NetworkInfer (52). All of the SARS-CoV-2 genomes analyzed in this article are publicly accessible through the GISAID platform and the COG-UK consortium. The sequence identifiers analyzed in this study are available at https://github.com/Hallatscheklab/NetworkInfer.
