# Supplementary material for: Uncovering heterogeneous intercommunity disease transmission from neutral allele frequency time series
Source: Proc Natl Acad Sci U S A. 2025 Nov 26;122(48):e2500663122. doi: 10.1073/pnas.2500663122 (PMC12684928; doi:10.1073/pnas.2500663122)
Supplement: Supplementary file 1 — Appendix 01 (PDF) [file pnas.2500663122.sapp.pdf]

Supplementary Information

Uncovering heterogeneous disease transmission
from frequency time series of neutral variants

Takashi Okada, Giulio Isacchini, Qinqin Yu, and Oskar Hallatschek

Table of Contents for SI

S.1 Data sources and processing . . . 2
S.1.1 Lineage frequency data . . . 2
S.1.2 From lineages to alleles . . . 2
S.1.2.1 Mutation frequency data . . . 2
S.1.2.2 Outlier detection . . . 4
S.1.3 Mobility data in the United States . . . 4
S.1.4 Autocorrelation functions, R_{ij}, in Fig. 1D . . . 5
S.2 Hidden Markov Model for the neutral frequency dynamics . . . 6
S.2.1 Hidden Markov Model . . . 6
S.2.2 Inference . . . 7
S.2.2.1 Computing the likelihood function and the filtered trajectories using the forward algorithm . . . 7
S.2.2.2 Computing the posterior distributions using an MCMC . . . 8
S.2.2.3 Computing the maximum likelihood estimation through an EM algorithm . . . 9
S.2.3 Computational tests . . . 10
S.2.4 Simulation of Figure 1A . . . 11
S.2.5 Effects of estimation errors in matrix elements $\mathbf{A}_{ij}$ on eigenvalues and eigenvectors . . . 11
S.3 Reproductive values in England . . . 14
S.3.1 Ranking regions according to ratios of bidirectional importation rates . . . 14
S.3.2 Testing the effect of the delay in sequence reporting . . . 14
S.3.3 Perturbative analysis of the effect of delay in reporting . . . 15
S.4 Spatial Resolution and Inference Reliability . . . 17
S.5 Detailed balance condition . . . 19
S.6 Epidemiological interpretation of $\mathbf{A}$ . . . 20
S.6.1 Neutral case . . . 20
S.6.2 Non-neutral variant . . . 21
S.6.3 Other epidemiological scenarios . . . 21
S.7 The jump-size distribution calculated from the importation-rate matrix $\mathbf{A}$ . . . 23
S.8 Distance between demes used for the hierarchical clustering of $\mathbf{A}_{ij}$ and multidimensional scaling analysis . . . 23
S.9 Constrained inference without long-range transmission . . . 25
S.10 Prediction of the spreading dynamics of the Delta variant shown in Fig. 8 . . . 26
S.11 Multiplicity-Adjusted Bayesian Detection of Directional Asymmetry . . . 28
S.11.1 Asymmetry in the 3x3 importation-rate matrix $\mathbf{A}_{ij}$ in Fig. 4 . . . 28
S.11.2 Temporal shift in $\mathbf{A}_{ij}$ in Fig. 4 . . . 28
S.11.3 Heterogeneity in reproductive values in Fig. 7 . . . 29
S.12 Other supplementary figures . . . 31
S.13 Construction of demes . . . 38
S.13.1 Construction of demes in England . . . 38
S.13.2 Construction of demes in the US . . . 40
S.14 Calendar Dates and Weeks Since December 29, 2019 . . . 41
S.15 Mutation sets of SARS-CoV-2 Variants in England and the US . . . 42
S.16 Legend of Supplementary Movie of Figure 8 in the Main Text . . . 43

S.1 Data sources and processing

For the analysis of England, we downloaded the sequence metadata from the COVID-19 Genomics UK Consortium (COG-UK) (1) on March 25, 2022. The metadada include the time and location of sample collection. The number of sequences over time is presented in Fig. S32. For the analysis of the United States, the sequence metadata was obtained from the GISAID database (https://www.gisaid.org/). For the Delta variant analysis, we excluded the AY.4.2 sequences, whose proportion modestly increased in England during the Delta wave (2). For the Omicron variant analysis, we focused on the B.1.1.529 and BA.1 lineages, except for the BA.1 sequences that had any of the mutations S:K417N, S:N440K, or S:G446S (3).

S.1.1 Lineage frequency data.

While the metadata include the lineage designation using the Pango nomenclature (4, 5), it classifies variants into a limited number of lineages. Therefore, we created our own lineages based on phylogenetic distance using the publicly available COG-UK phylogenetic trees (on March 25, 2022) (6); specifically, we cut the tree at a particular depth to create many subtrees, defining each subtree as a lineage (see Fig. S1A). If any subtrees occupy more than 2.5% of the total sequences, we introduce an additional cut at another position (farther from the root) and divide these subtrees further to create more subtrees (lineages). We continue this process until no subtree occupies more than 2.5% of the total sequences. For the analysis of the Delta variant, the tree was cut at the depths at $50.5l_{\mathrm{unit}}$, $56.5l_{\mathrm{unit}}$, $59.5l_{\mathrm{unit}}$ where $l_{\mathrm{unit}} = 3.34 \times 10^{-5}$. Fig. S1B) shows how the sequences are distributed along the depth of the tree over epiweeks. Note that in this figure and throughout the SI, we adopt epiweeks starting from December 29, 2019, extending their application across multiple years to enable continuous analysis despite the usual yearly reset. The correspondence between the epiweeks and calendar dates is summarized in Sec. S.14.

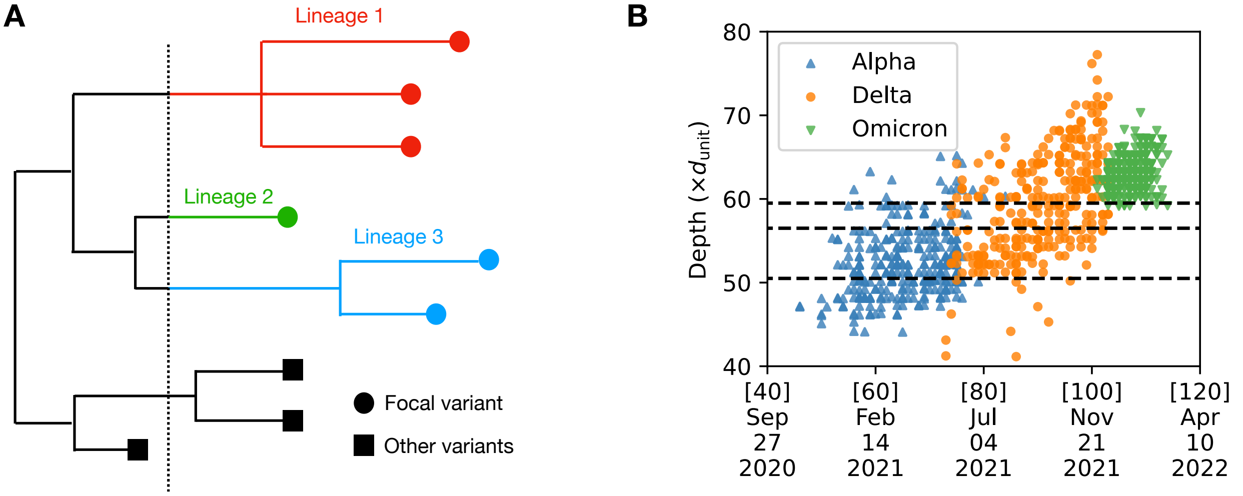

Fig. S1. (**A**) Construction of lineages within a variant using the phylogenetic tree: Leaf nodes are represented by circles for sequences of the focal variant and by squares for sequences of other variants. Lineages of the focal variant are defined by cutting the tree at a certain depth, represented by a vertical dashed line. In this illustration, three lineages are obtained through this procedure. (**B**) Collection date versus tree depth for the metadata sequences of the Alpha, Delta, and Omicron variants: For each variant, only 300 sequences are displayed for clearer visualization. The dashed horizontal lines represent the cuts used to define the lineages of the Delta variant in England. The numbers [·] on the dates denote epiweeks.

S.1.2 From lineages to alleles.

Our inference technique is fueled by data collected through counting the prevalence of *independent* lineages across locations and times. Identifying these independent lineages is straightforward when a complete and accurate phylogenetic tree is available, as it simply involves segmenting the tree into monophyletic groups. But constructing a large phylogenetic tree in the first place—in the case of SARS-CoV-2 for millions of viral genomes—is not only computationally demanding but often results in unresolved polytomies.

A tree-free alternative for creating time series of neutral lineages is provided by tracking the frequency of all pre-existing neutral *mutations* in the different sub-populations. This works in principle because the frequency of a neutral allele has to obey Eq. 1 of the main text, even in the presence of recombination. The downside of this approach is that, due to linkage, different alleles may not be independent. Treating them as independent underestimates statistical errors. Moreover, it is important to ensure that the included alleles are neutral.

Therefore, as described below, we first cluster alleles based on their pairwise genetic distances and select representative alleles for each cluster (7) (see Sec S.1.2.1). We then exclude a small fraction of outlier alleles, whose time series are not consistent with neutrality, as measured by a maximum likelihood method described in refs. (6, 8) (see Sec. S.1.2.2).

Although our analyses primarily use the allele-based approach, Figs. S3 and S27 demonstrate that, regardless of whether tree-based lineages or alleles are used (including only synonymous mutations or all mutations), the fine-scale and coarse-grained outcomes—including the largest eigenvalues and reproductive values—are consistent and do not differ significantly for the Delta wave in England.

S.1.2.1 Mutation frequency data.

Mutation frequency data for England and the USA were generated from mutations listed in the COG-UK and GISAID metadata, respectively. In our analysis, we used both synonymous and non-synonymous mutations from the COG-UK metadata, whereas only non-synonymous mutations were used from the GISAID metadata. The frequency of each mutation was calculated by counting the number of sequences carrying that mutation and normalizing this by the total number of sequences at each sampled location, with a unit time of

one week. For very rare mutations, the effect of sampling noise is significant, making the inference unreliable. Conversely, including very abundant mutations would limit the total number of independent mutations (obtained from a method described below (7)). Therefore, as a compromise, we decided to select mutations whose country-wide frequency, averaged over a focal time window, is moderately low, ranging between 0.003 and 0.05.

The presence of genetic linkage between mutations can create statistical dependencies that can distort our inference. To avoid bias due to linkage-induced correlations, we pruned the set of mutations following ref. (7). The pruning procedure first defines a distance between two mutations m_1 and m_2 as

$$d(m_1, m_2) = 1 - \frac{|S_1 \cap S_2|}{|S_1 \cup S_2|}, \qquad \text{[S.1]}$$

where S_i is the set of sequences carrying the mutation m_i in a country. Next, we constructed a graph where nodes are mutations, and an edge exists between two mutations if $d(m_1, m_2) < d_{\rm th}$, with $d_{\rm th} \in [0, 1]$ as a threshold parameter. We then identified the connected components of the graph, treating each connected component as a cluster of mutations. Finally, from each cluster, we selected the mutation m_i with the largest $|S_i|$ as its representative, producing a set of approximately independent mutations.

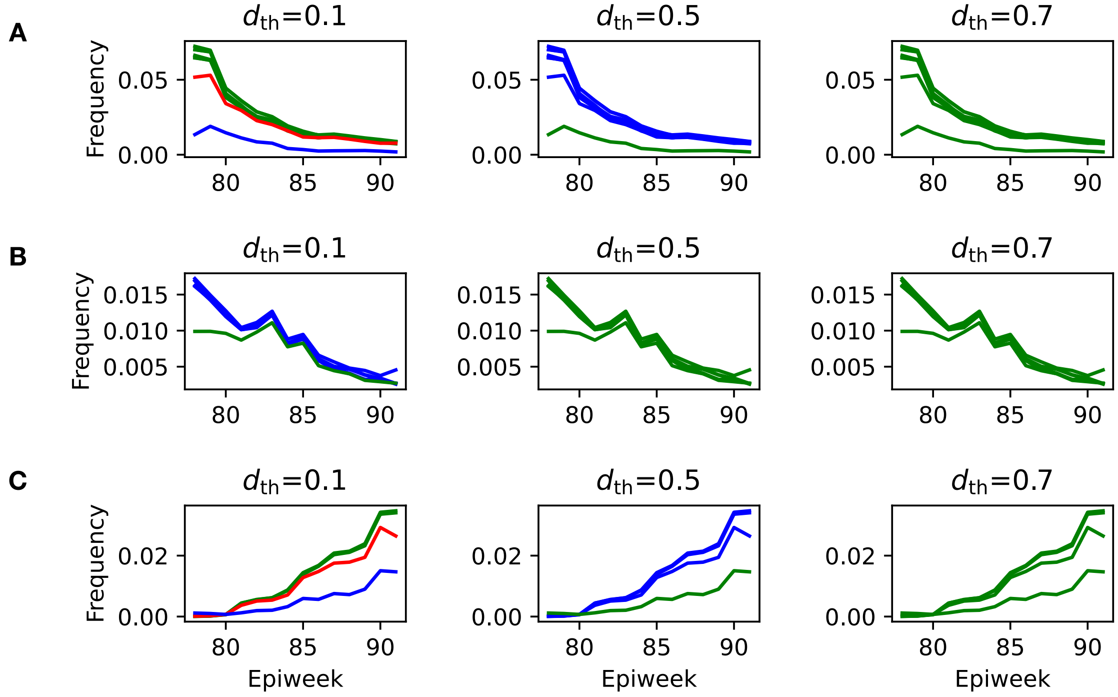

Fig. S2. (**A**) The country-wide frequency trajectories of a subset of mutations of the Delta variant in England. For each threshold value $d_{\rm th}$, the frequency trajectories of mutations that belong to the same cluster are plotted in the same color. Nine mutations are classified into three, two, and one clusters for $d_{\rm th} = 0.1, 0.5$, and 0.7, respectively. Similarly, Figures (**B**) and (**C**) show the frequency trajectories and the equivalence classes for other subsets of mutations of the Delta variant in England.

The higher the threshold value $d_{\rm th}$, the more mutation trajectories are grouped into the same cluster, as illustrated for the the Delta variant in England in Figure S2. We confirmed that as $d_{\rm th}$ increases, the importation-rate matrix for the Delta variant inferred from the mutation data becomes more similar to the matrix inferred from the lineage data (Figure S3). Based on these results, we decided to use a high value of $d_{\rm th} = 0.9$ for all the inferences presented in this paper (except for Figure S3).

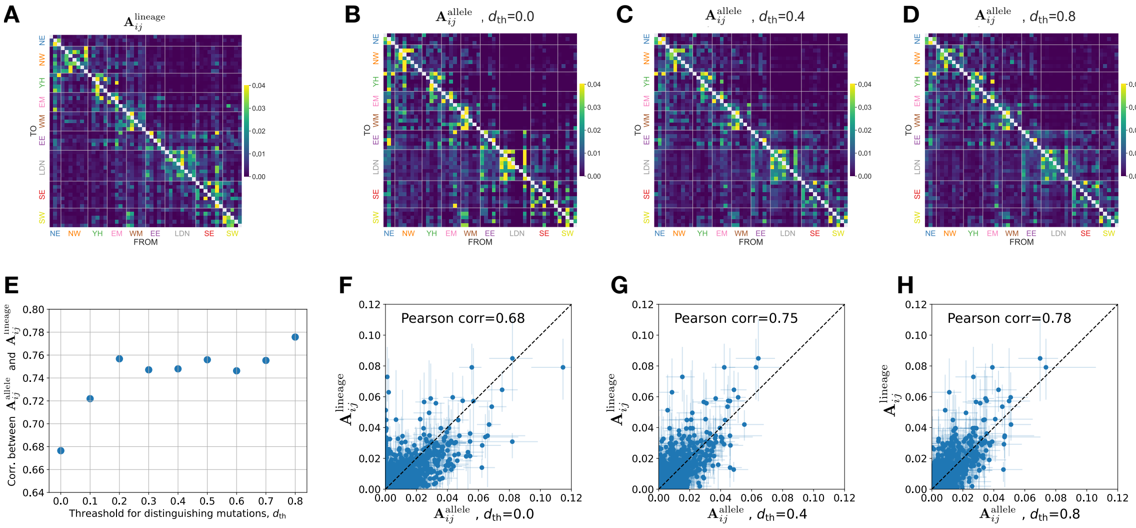

Fig. S3. Comparison between the result for the Delta-sublineage data in England and the result for the mutation data, and the dependence on the threshold value d_{th} used for clustering mutations. $N_D = 50$. (**A**) Importation-rate matrix, $\mathbf{A}_{ij}^{\text{lineage}}$, inferred from the lineage data. (**B**-**D**) Importation-rate matrix, $\mathbf{A}_{ij}^{\text{mutaton}}$, inferred from the mutation data, where $d_{\text{th}} = 0.0, 0.4, 0.8$, respectively. (**E**) d_{th} vs the Pearson correlation coefficient between $\mathbf{A}_{ij}^{\text{lineage}}$ and $\mathbf{A}_{ij}^{\text{mutation}}$. F-H) Element-wise comparison of $\mathbf{A}_{ij}^{\text{lineage}}$ and $\mathbf{A}_{ij}^{\text{mutation}}$ for $d_{\text{th}} = 0.0, 0.4, 0.8$, respectively.

S.1.2.2 Outlier detection.

In our inference, we focused on alleles within a particular variant, as our method relies on the neutrality assumption, and mixing alleles from different variants could introduce fitness differences that violate this assumption. However, there is still a possibility that significant differences in fitness exist between alleles even within a variant, which could potentially bias the inference of the importation-rate matrix. To prevent this, we applied the statistical test for neutrality (6, 8), which computes the maximum likelihood estimate of the relative fitness s and the p value for each allele, from the time-series data of allele counts.

For the mutations selected by the clustering described in Sec. S.1.2.1, we applied this statistical test using their country-wide count data. We then excluded significantly non-neutral mutations ($p < 0.05$) and used only putatively neutral mutations ($p \geq 0.05$) for our inference. Figure S4A displays the results of the statistical test on the Delta variant in England. From left to right, it shows the allele-frequency trajectories for putatively neutral alleles (left), significantly non-neutral alleles (middle), and the p-s distribution (right). Among the 85 representative mutations identified by the clustering method described in Sec. S.1.2.1, 68 mutations are identified as putatively neutral. As a control, we also applied the statistical test to the simulated data of neutral allele frequencies generated by the Wright-Fisher model (Fig. S4B). For both the actual and simulated data, the central region of the p-s distribution exhibits a triangular shape, indicating the validity of the neutrality assumption for the majority of mutations of the Delta variant sitting in this central region of the p-s distribution. The complete list of alleles used as input to our HMM method is provided in the table in Sec. S.15.

Similarly, we applied the same filtering to the lineage data and used only putatively neutral lineages for the inference.

S.1.3 Mobility data in the United States.

In the analysis of the jump-size distribution presented in Fig. 6C, we used SafeGraph data (9), which were derived from cell phone tracking. Specifically, we used the county-level dataset processed by the authors of ref. (10) and then aggregated spatial locations to obtain the mobility flux between the 30 demes. Note that while we compared the jump-size distribution inferred from the sequencing data during the Delta wave with that calculated from the SafeGraph data, the SafeGraph dataset spans March 2020 to February 2021, which predates the surge of the Delta variant.

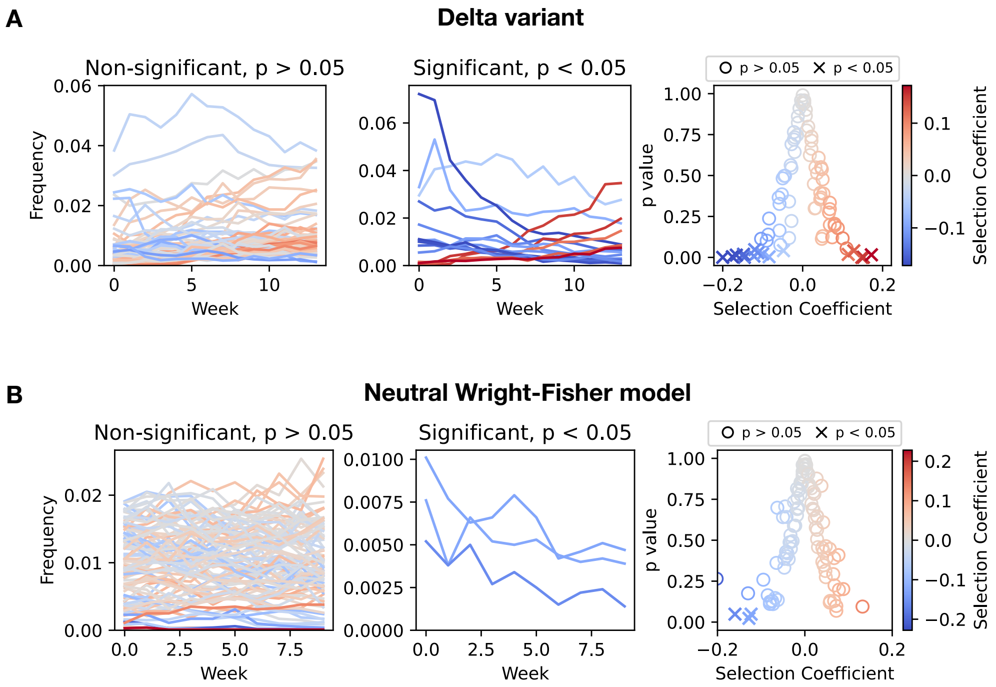

Fig. S4. (**A**) Trajectories of mutation frequencies observed in the Delta wave in England (Left: trajectories with $p > 0.05$, Middle: trajectories with $p < 0.05$). Right: Inferred selective coefficient and p-value, where each dot represents these values for a particular mutation. (**B**) Trajectories generated by the neutral Wright-Fisher simulation (Left: trajectories with $p > 0.05$, Middle: trajectories with $p < 0.05$). The effective population size is set to 10,000, the sampling rate per week is set to 10,000, and the number of trajectories is 100, comparable to actual country-wide data in England. Right: Inferred selective coefficient and p-value.

S.1.4 Autocorrelation functions, R_{ij}, in Fig. 1D.

In Fig. 1D, we demonstrate the convergence of allele frequencies across regions in England by computing the autocorrelation functions $R_{ij}(\tau)$, defined as

$$R_{ij}(\tau) = \frac{1}{N_\nu N_t} \sum_{\nu} \sum_{t} \left(X_i^\nu(t+\tau) - X_j^\nu(t+\tau) \right) \left(X_i^\nu(t) - X_j^\nu(t) \right), \qquad \text{[S.2]}$$

where ν and t label alleles and timepoints, respectively; N_ν and N_t denote the total numbers of alleles and timepoints, respectively; and $X_i^\nu(t)$ denotes the frequency of allele μ in region i at week t. We also calculated the autocorrelation functions using the lineage frequencies of the Delta variant and confirmed that decay rates similar to those presented in Fig. 1D are obtained from the lineage data (Fig. S5).

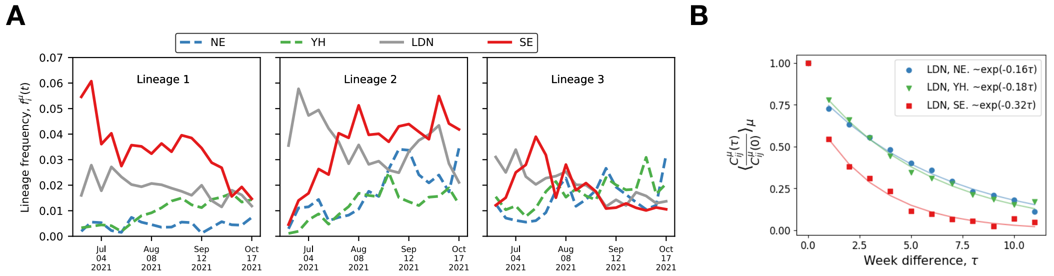

Fig. S5. (**A**) The time-series trajectories of lineage frequencies are illustrated for three lineages of the Delta variant. (**B**) The dots show the normalized autocorrelation functions R_{ij} between London and North East (blue), London and Yorkshire and the Humber (green), London and South East (red), computed from the lineage frequency data. The solid lines show exponential curves fitted to the data. The auto-orrelation functions are computed by averaging over the 40 lineages with the largest sample sizes.

S.2 Hidden Markov Model for the neutral frequency dynamics

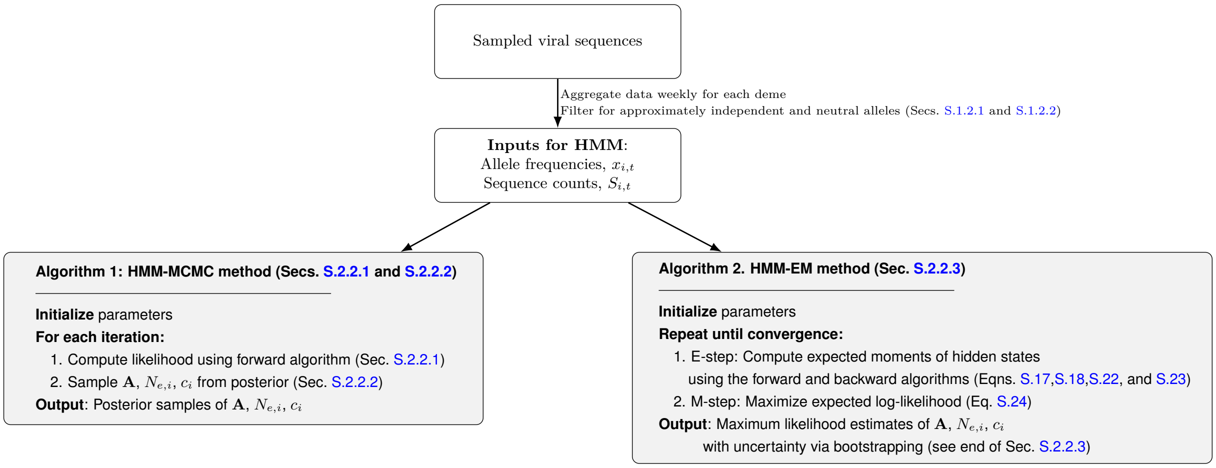

Fig. S6. Overview of the inference pipeline. Starting from sampled viral genomes, we filter mutations to retain approximately independent and neutral alleles. The dynamics of the allele frequencies are modeled using a HMM, and inference is performed via EM or MCMC algorithms, resulting in estimates of importation rates, effective population sizes, and measurement noise.

Our inference method is based on a dynamic linear model (a Kalman filter). The overview of the inference pipeline is shown in Fig. S6. The Python scripts and the C++ code for our inference method are available at https://github.com/Hallatscheklab/NetworkInfer.

We follow the notation and formulation in ref. (11). In the following, we denote the multivariate normal distribution with mean $\boldsymbol{\mu}$ and covariance $\boldsymbol{\Sigma}$ as

$$N(\boldsymbol{x}|\boldsymbol{\mu},\boldsymbol{\Sigma}) \equiv \frac{1}{\sqrt{(2\pi)^k|\boldsymbol{\Sigma}|}} \exp\left(-\frac{1}{2}(\boldsymbol{x}-\boldsymbol{\mu})^\top \boldsymbol{\Sigma}^{-1}(\boldsymbol{x}-\boldsymbol{\mu})\right), \qquad \text{[S.3]}$$

where k is the dimensionality of $\boldsymbol{x}$. We denote the function measuring "heterozygosity", $x(1-x)$, as

$$H(x) \equiv x(1-x). \qquad \text{[S.4]}$$

S.2.1 Hidden Markov Model.

We consider the neural dynamics of a lineage (or allele) frequency described by the spatial Wright-Fisher model with N_D demes. The dynamics of the true and observed frequencies can be approximately described by the following Markov process (Fig. S7).

- *Transition probability distribution*: The true frequency vector $\boldsymbol{z}_t \in [0,1]^{N_D}$ (hidden state) obeys

$$p(\boldsymbol{z}_t|\boldsymbol{z}_{t-1}) = \mathcal{N}(\boldsymbol{z}_t|\mathbf{A}\boldsymbol{z}_{t-1},\boldsymbol{\Gamma}_{t-1}) \qquad \text{[S.5]}$$

  with the importation-rate matrix $\mathbf{A} \in \mathbb{R}^{N_D\times N_D}$ satisfying $\mathbf{A}_{ij} > 0$ $(i \neq j)$ and $\sum_j \mathbf{A}_{ij} = 1$, and

$$\begin{aligned}\boldsymbol{\Gamma}_{t-1} &= \text{diag.}\left(\frac{(\mathbf{A}\boldsymbol{z}_{t-1})_i(1-(\mathbf{A}\boldsymbol{z}_{t-1})_i)}{N_{\text{e},i}}\right)\\ &:= \text{diag.}\left(\frac{H((\mathbf{A}\boldsymbol{z}_{t-1})_i)}{N_{\text{e},i}}\right)\end{aligned} \qquad \text{[S.6]}$$

  Here $(\mathbf{A}\boldsymbol{Z})_i = \sum_j \mathbf{A}_{ij}z_j$, and $N_{\text{e},i}$ is the effective population size of deme i, which controls the strength of the genetic drift.

- *Emission probability distribution*: The observed frequency vector $\boldsymbol{x}_t \in [0,1]^{N_D}$ approximately obeys:

$$p(\boldsymbol{x}_t|\boldsymbol{z}_t) = \mathcal{N}(\boldsymbol{x}_t|\boldsymbol{z}_t,\boldsymbol{\Sigma}_t), \qquad \text{[S.7]}$$

  where $\boldsymbol{\Sigma}_t$ characterizes the strength of the measurement error and is assumed to be given by

$$\boldsymbol{\Sigma}_t = \text{diag.}\left(c_i\frac{H(z_{i,t})}{S_{i,t}}\right). \qquad \text{[S.8]}$$

  Here $S_{i,t}$ is the number of sequences at week t in deme i, and $c_i \geq 1$ is a parameter quantifying the deviation from ideal random sampling. The quantity $S_{i,t}/c_i$ can be interpreted as the *effective sampling size*.

- *Initial condition on* z_0:

$$p(\boldsymbol{z}_{t=0}) = \mathcal{N}(\boldsymbol{z}_{t=0}|\boldsymbol{\mu}_*,\boldsymbol{V}_*). \qquad \text{[S.9]}$$

  We assume that $\boldsymbol{\mu}_*$ and $\boldsymbol{V}_*$ are given by

$$\begin{aligned}\boldsymbol{\mu}_* &= \boldsymbol{x}_{t=0}\\ \boldsymbol{V}_* &= \text{diag.}\left(\frac{H(x_{0,t=0})}{S_{0,t=0}},\ldots,\frac{H(x_{N_D,t=0})}{S_{N_D,t=0}}\right).\end{aligned} \qquad \text{[S.10]}$$

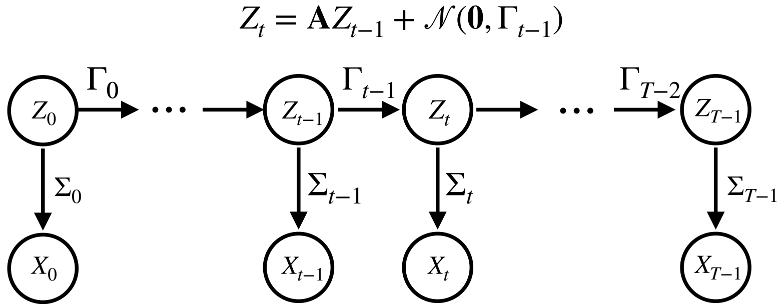

Fig. S7. The HMM for the frequency dynamics. Z_t and X_t are true frequencies and observed frequencies, respectively. The transition probability densities (horizontal arrows) and emission probability densities (vertical arrows) are modeled by Gaussian distributions, Eqns. (S.5) and (S.7).

S.2.2 Inference.

Given the observed frequencies, $x_{i,t}$, and the number of sampled sequence, $S_{i,t}$, our goal is to estimate the importation-rate matrix $\mathbf{A}$, the effective population sizes $N_{\mathrm{e},i}$, and the overdispersion of measurement noise c_i. To achieve this, we developed two algorithms:

- Inference of the posterior distributions using a Markov Chain Monte Carlo (MCMC) method.
- Maximum likelihood estimation using an EM algorithm.

We employed the MCMC method for the region-level inference in England. Conversely, for deme-resolved matrices (Figs. 3A and 6A), we chose the EM method because of intensive computational requirements of the MCMC approach.

S.2.2.1 Computing the likelihood function and the filtered trajectories using the forward algorithm.

Here, we describe the forward algorithm, which recursively computes both the likelihood and the posterior distribution of the hidden state, conditioned on observations up to time point t. We have the identity:

$$\begin{aligned} p(\boldsymbol{z}_{t+1}|\boldsymbol{x}_{0:t+1}) &= p(\boldsymbol{z}_{t+1}|\boldsymbol{x}_{t+1}, \boldsymbol{x}_{0:t}) \\ &= \frac{p(\boldsymbol{z}_{t+1}, \boldsymbol{x}_{t+1}|\boldsymbol{x}_{0:t})}{p(\boldsymbol{x}_{t+1}|\boldsymbol{x}_{0:t})} \quad (\because \text{Product rule}) \\ &= \frac{p(\boldsymbol{x}_{t+1}|\boldsymbol{z}_{t+1}, \boldsymbol{x}_{0:t})p(\boldsymbol{z}_{t+1}|\boldsymbol{x}_{0:t})}{p(\boldsymbol{x}_{t+1}|\boldsymbol{x}_{0:t})} \quad (\because \text{Product rule}) \\ &= \frac{p(\boldsymbol{x}_{t+1}|\boldsymbol{z}_{t+1})p(\boldsymbol{z}_{t+1}|\boldsymbol{x}_{0:t})}{p(\boldsymbol{x}_{t+1}|\boldsymbol{x}_{0:t})} \quad (\because \text{Conditional independence}) \\ \log p(\boldsymbol{z}_{t+1}|\boldsymbol{x}_{0:t+1}) &= \log p(\boldsymbol{x}_{t+1}|\boldsymbol{z}_{t+1}) + \log p(\boldsymbol{z}_{t+1}|\boldsymbol{x}_{0:t}) + \underbrace{\text{const.}}_{z-\text{indep.}} \end{aligned} \qquad [\text{S.11}]$$

Because the second term $p(\boldsymbol{z}_{t+1}|\boldsymbol{x}_{0:t})$ can be rewritten as

$$\begin{aligned} p(\boldsymbol{z}_{t+1}|\boldsymbol{x}_{0:t}) &= \int p(\boldsymbol{z}_{t+1}, \boldsymbol{z}_t|\boldsymbol{x}_{0:t})d\boldsymbol{z}_t \quad (\because \text{Marginalization}) \\ &= \int p(\boldsymbol{z}_{t+1}|\boldsymbol{z}_t, \boldsymbol{x}_{0:t})p(\boldsymbol{z}_t|\boldsymbol{x}_{0:t})d\boldsymbol{z}_t \quad (\because \text{Product rule}) \\ &= \int p(\boldsymbol{z}_{t+1}|\boldsymbol{z}_t)p(\boldsymbol{z}_t|\boldsymbol{x}_{0:t})d\boldsymbol{z}_t \quad (\because \text{Conditional independence}) \end{aligned} \qquad [\text{S.12}]$$

we have

$$\log p(\boldsymbol{z}_{t+1}|\boldsymbol{x}_{0:t+1}) = \log p(\boldsymbol{x}_{t+1}|\boldsymbol{z}_{t+1}) + \log \int p(\boldsymbol{z}_{t+1}|\boldsymbol{z}_t)p(\boldsymbol{z}_t|\boldsymbol{x}_{0:t})d\boldsymbol{z}_t + \text{const.} \qquad [\text{S.13}]$$

In the standard Kalman filter, a crucial step in deriving the recursion equations is performing Gaussian integration over the hidden state variables $\boldsymbol{z}$. In our model, the covariance matrices $\boldsymbol{\Gamma}_t$ and $\boldsymbol{\Sigma}_t$ are dependent on $\boldsymbol{z}$, which makes the process non-Gaussian. To utilize the technique of the Kalman filter, we approximate the covariances using matrices that are independent of $\boldsymbol{z}$;

$$\begin{aligned} \boldsymbol{\Gamma}_{t-1} &\approx \text{diag.}\left(\frac{H(\bar{x}_{t,i})}{N_{\mathrm{e},i}}\right), \\ \boldsymbol{\Sigma}_t &\approx \text{diag.}\left(c_i \frac{H(\bar{x}_{i,t})}{S_{i,t}}\right), \end{aligned} \qquad [\text{S.14}]$$

where the hidden frequency $z_{i,t}$ is replaced by the time-averaged observed frequency $\bar{x}_{i,t} \equiv \frac{1}{T}\sum_{t=0}^{T-1} x_{i,t}$. Under this approximation, all quantities appearing in Eq. (S.13) become Gaussian. We note that, unlike the standard Kalman filter, the covariance matrix Σ_t in our model is time-dependent due to the time-varying sampling rates $S_{i,t}$. However, we can still perform Gaussian integrations even with the time-dependent covariance, as long as it remains independent of the hidden variables $\boldsymbol{z}_t$.

After some calculations (as detailed in ref. (11)), it can be shown that the triplet $\{\boldsymbol{\mu}_t, \boldsymbol{V}_t, l_t\}$, defined by

$$p(\boldsymbol{z}_t|\boldsymbol{x}_{0:t}) = \mathcal{N}(\boldsymbol{z}_t|\boldsymbol{\mu}_t, \boldsymbol{V}_t) \qquad [\text{S.15}]$$

$$l_t = p(\boldsymbol{x}_t|\boldsymbol{x}_{0:t-1}), \qquad [\text{S.16}]$$

satisfies the following recursive equations:

Forward algorithm

– For $t = 0$,

$$\begin{aligned} \boldsymbol{\mu}_0 &= \boldsymbol{\mu}_* + \boldsymbol{K}_0(\boldsymbol{x}_0 - \boldsymbol{\mu}_*) \\ \boldsymbol{V}_0 &= (\boldsymbol{I} - \boldsymbol{K}_0)\boldsymbol{V}_* \\ l_0 &= \mathcal{N}(\boldsymbol{x}_0 | \boldsymbol{\mu}_*, \boldsymbol{V}_* + \boldsymbol{\Sigma}_0) \end{aligned} \qquad \text{[S.17]}$$

where $\boldsymbol{K}_0 = \boldsymbol{V}_*(\boldsymbol{V}_* + \boldsymbol{\Sigma}_0)^{-1}$.
For $t = 1, 2, \ldots, T-1$,

$$\begin{aligned} \boldsymbol{\mu}_t &= \mathbf{A}\boldsymbol{\mu}_{t-1} + \boldsymbol{K}_t(\boldsymbol{x}_t - \mathbf{A}\boldsymbol{\mu}_{\boldsymbol{t-1}}) \\ \boldsymbol{V}_t &= (\boldsymbol{I} - \boldsymbol{K}_t)\boldsymbol{P}_{t-1} \\ l_t &= \mathcal{N}(\boldsymbol{x}_t | \mathbf{A}\boldsymbol{\mu}_{t-1}, \boldsymbol{P}_{t-1} + \boldsymbol{\Sigma}_t) \end{aligned} \qquad \text{[S.18]}$$

where $\boldsymbol{P}_{t-1} = \mathbf{A}\boldsymbol{V}_{t-1}\mathbf{A}^\top + \boldsymbol{\Gamma}_{t-1}$ and $\boldsymbol{K}_t = \boldsymbol{P}_{t-1}(\boldsymbol{P}_{t-1} + \boldsymbol{\Sigma}_t)^{-1}$.

– The likelihood function is given by

$$p(x_0, \ldots, x_{T-1}) = \prod_{t=0}^{T-1} l_t. \qquad \text{[S.19]}$$

For each lineage (or allele) labeled by ν, we compute the triplet $\{\boldsymbol{\mu}_t^\nu, \boldsymbol{V}_t^\nu, l_t^\nu\}_{0 \le t \le T}$ by solving Eqns. (S.17, S.18) recursively, with μ_* and V_* in Eq. (S.10) and Σ_t and Γ_t in Eq. (S.14). The total log likelihood of the time-series data for all lineages (or alleles) is given by

$$\log p(\text{Data}|\Theta) = \sum_{t=1}^{T-1} \sum_{\nu} \log l_t^\nu \equiv -E(\Theta), \qquad \text{[S.20]}$$

where Θ is the set of the parameters to be inferred, $\Theta = \{\mathbf{A}_{ij},\ N_{\text{e},i},\ c_i\}$.

We note that in Eq. (S.20), the log likelihood at the initial timepoint, $\log l_{t=0}^\nu$, is excluded from the summation (even though l_0 appears in the likelihood in Eq. (S.19)). This exclusion is appropriate due to our assumption about the true initial frequency, $\boldsymbol{\mu}_* = \boldsymbol{x}_0$. Under this assumption, Eq. (S.17) leads to $\log l_{t=0}^\nu = -\frac{1}{2}\log|\boldsymbol{V}_* + \boldsymbol{\Sigma}_0| + \text{constant}$, which increases as the overdispersion parameter of measurement noise, c_i, decreases (see $\boldsymbol{\Sigma}_0$ in Eq. (S.14)), regardless of the values of measured frequencies. Therefore, including the term $\log l_{t=0}^\nu$ in Eq. (S.20) would introduce a bias that underestimates c_i when maximizing the likelihood.

S.2.2.2 Computing the posterior distributions using an MCMC.

We consider the flat prior distributions $p_{\text{prior}}(\Theta)$ on Θ. In this case, the posterior distribution is proportional to the likelihood,

$$p(\Theta|\text{Data}) = \frac{p(\text{Data}|\Theta)p_{\text{prior}}(\Theta)}{\int p(\text{Data}|\Theta)p_{\text{prior}}(\Theta)d\Theta} = \mathcal{C} \times p(\text{Data}|\Theta) \equiv \mathcal{C} \times e^{-E(\Theta)}, \qquad \text{[S.21]}$$

where $\mathcal{C}$ is a factor independent of Θ.

To obtain the posterior distribution $p(\Theta|\text{Data}) \propto e^{-E(\Theta)}$, we perform an MCMC method, namely, simulate a random walk in the Θ space whose stationary distribution is given by $p(\Theta|\text{Data}) \propto e^{-E(\Theta)}$. Specifically, we implement the Metropolis algorithm:

MCMC (Metropolis algorithm)

- Initialize Θ_0.
- Compute $E(\Theta_0)$ using the forward algorithm (Eq. S.20).
- For $l = 0$ to $L-1$,
  - Propose $\Theta' \sim p_{\text{proposal}}(\Theta'|\Theta_l)$ (explained below).
  - Compute $E(\Theta')$ using the forward algorithm (Eq. S.20).
  - Sample u from the uniform distribution on $[0, 1]$.
  - If $u < e^{-(E(\Theta') - E(\Theta))}$, set $\Theta_{l+1} = \Theta'$. Else set $\Theta_{l+1} = \Theta_l$.

For sufficiently large Monte Carlo steps L (e,g., $L \sim 10^6$), the sequence $\Theta_0, \Theta_1, \ldots, \Theta_L$ approximates the posterior distribution $p(\Theta|\text{Data})$.

For the proposal distribution p_{proposal}, we employ the followings:

- For $N_{\text{eff,i}}$, sample $h \sim \mathcal{N}(0, \epsilon_N)$, where ϵ_N is a positive constant. If $N_{\text{eff,i}} + h \ge 0$, set $N'_{\text{eff,i}} = N_{\text{eff,i}} + h$. Else set $N'_{\text{eff,i}} = -(N_{\text{eff,i}} + h)$.
- For $c_i \in \Theta$, sample $h \sim \mathcal{N}(0, \epsilon_c)$, where ϵ_c is a positive constant. If $c_i + h \ge 1$, set $c'_i = c_i + h$. Else set $c'_i = 2 - (c_i + h)$.
- For the importation-rate matrix $\mathbf{A}_{ij}$, randomly choose a pair (i, j) and sample $\epsilon \in [-\mathbf{A}_{ii}, \mathbf{A}_{ij}]$ from the uniform distribution. Set $\mathbf{A}'_{ij} = \mathbf{A}_{ij} - \epsilon$ and $\mathbf{A}'_{ii} = \mathbf{A}_{ii} + \epsilon$.

Remark: While one may use other forms of $p_{\text{proposal}}(\Theta'|\Theta)$, it must satisfy two critical conditions:

1. The proposed Θ' must be within the meaningful parameter region. For example, $N'_{\text{eff},i}$ must be positive, and $\mathbf{A}'_{i,j}$ must lie on the simplex.
2. $p_{\text{proposal}}(\Theta'|\Theta)$ must be symmetric, i.e., $p_{\text{proposal}}(\Theta'|\Theta) = p_{\text{proposal}}(\Theta|\Theta')$. Otherwise, the Metropolis-Hastings algorithm should be used instead of the Metropolis algorithm.

Note that in our proposal distribution for $N_{\text{e},i}$ and c_i, any proposed value that exceeds the boundary of the parameter space is reflected along the boundary. This prescription ensures the symmetric condition near the boundary. For $\mathbf{A}_{ij}$, our proposal distribution also satisfies the above two conditions (see ref. (12)).

S.2.2.3 Computing the maximum likelihood estimation through an EM algorithm.

We use an EM algorithm to search for the maximum likelihood estimate of the parameters Θ. Suppose that the model parameter is $\Theta^{\rm old} = (\mathbf{A}, \boldsymbol{N}_{\rm e})^{\rm old}$ at a stage of the algorithm. By using $\Theta^{\rm old}$, for each lineage, we first compute the filtered trajectory $\boldsymbol{\mu}_t$ and the covariance $\boldsymbol{V}_t$, which characterize the posterior distribution of the hidden state conditioned on the observations up to time t, by applying the forward algorithm. Then, we compute the smoothed trajectory $\hat{\boldsymbol{\mu}}_t$ and the variance $\hat{\boldsymbol{V}}_t$ by solving the backward equations, which refine the state estimates using future observations:

$$\hat{\boldsymbol{\mu}}_t = \boldsymbol{\mu}_t + \boldsymbol{J}_t(\hat{\boldsymbol{\mu}}_{t+1} - \mathbf{A}\boldsymbol{\mu}_t)$$
$$\hat{\boldsymbol{V}}_t = \boldsymbol{V}_t + \boldsymbol{J}_t(\hat{\boldsymbol{V}}_{t+1} - \boldsymbol{P}_t)\boldsymbol{J}_t^\top \qquad \text{[S.22]}$$

where $\boldsymbol{P}_t = \mathbf{A}\boldsymbol{V}_t\mathbf{A}^\top + \boldsymbol{\Gamma}_t$ and $\boldsymbol{J}_t = \boldsymbol{V}_t\mathbf{A}^\top(\boldsymbol{P}_t)^{-1}$. Solving the backward algorithm provides the following quantities, which will be required below*;

$$\mathbb{E}_{p(\boldsymbol{z}|\boldsymbol{x},\Theta)}[\boldsymbol{z}_t] = \hat{\boldsymbol{\mu}}_t$$
$$\mathbb{E}_{p(\boldsymbol{z}|\boldsymbol{x},\Theta)}[\boldsymbol{z}_t\boldsymbol{z}_{t-1}^\top] = \hat{\boldsymbol{V}}_t\boldsymbol{J}_{t-1}^\top + \hat{\boldsymbol{\mu}}_t\hat{\boldsymbol{\mu}}_{t-1}^\top$$
$$\mathbb{E}_{p(\boldsymbol{z}|\boldsymbol{x},\Theta)}[\boldsymbol{z}_t\boldsymbol{z}_t^\top] = \hat{\boldsymbol{V}}_t + \hat{\boldsymbol{\mu}}_t\hat{\boldsymbol{\mu}}_t^\top. \qquad \text{[S.23]}$$

In the EM algorithm, we need to minimize the expectation Q of the complete-data likelihood $p(\boldsymbol{z}, \boldsymbol{x}, \Theta)$ (Here, $\boldsymbol{z}$ and $\boldsymbol{x}$ collectively denote the frequencies $\boldsymbol{z}^\nu$ and $\boldsymbol{x}^\nu$ of all $N_{\rm lin}$ lineages). It can be shown that the terms of Q that are dependent on $\mathbf{A}_{ij}$ and $N_{{\rm e},i}$ are given by (see ref. (11))

$$\begin{aligned}
Q(\Theta, \Theta^{\rm old}) &= \mathbb{E}_{p(\boldsymbol{z}|\boldsymbol{x},\Theta^{\rm old})}[\ln p(\boldsymbol{z}, \boldsymbol{x}, \Theta)] \\
&= -\frac{1}{2}\sum_{\nu=1}^{N_{\rm lin}}\sum_{t=1}^{T-1} \ln|\Gamma_{t-1}^\nu| - \mathbb{E}_{p(\boldsymbol{z}|\boldsymbol{x},\Theta^{\rm old})}\left[\frac{1}{2}\sum_{t=1}^{T-1}\sum_{\nu=1}^{N_{\rm lin}}(\boldsymbol{z}_t^\nu - \mathbf{A}\boldsymbol{z}_{t-1}^\nu)^\top(\Gamma_{t-1}^\nu)^{-1}(\boldsymbol{z}_t^\nu - \mathbf{A}\boldsymbol{z}_{t-1}^\nu)\right] \\
&\quad + (\text{terms independent of } \mathbf{A}, N_{\rm e}) \\
&= \frac{N_{\rm lin}(T-1)}{2}\sum_i \ln N_{{\rm e},i} - \mathbb{E}_{p(\boldsymbol{z}|\boldsymbol{x},\Theta^{\rm old})}\left[\frac{1}{2}\sum_{t=1}^{T-1}\sum_{\nu=1}^{N_{\rm lin}}(\boldsymbol{z}_t^\nu - \mathbf{A}\boldsymbol{z}_{t-1}^\nu)^\top(\Gamma_{t-1}^\nu)^{-1}(\boldsymbol{z}_t^\nu - \mathbf{A}\boldsymbol{z}_{t-1}^\nu)\right] + \cdots \\
&= \sum_i \Bigg[\frac{N_{\rm lin}(T-1)}{2}\ln N_{{\rm e},i} \\
&\quad - \frac{1}{2}\sum_{t=1}^{T-1}\sum_{\nu=1}^{N_{\rm lin}} \frac{N_{{\rm e},i}}{H(\bar{x}_{i,t-1}^\nu)}\left(\mathbf{A}\mathbb{E}[\boldsymbol{z}_{t-1}^\nu\boldsymbol{z}_{t-1}^{\nu,{\rm T}}]\mathbf{A}^\top - \mathbb{E}[\boldsymbol{z}_t^\nu\boldsymbol{z}_{t-1}^{\nu,{\rm T}}]\mathbf{A}^\top - \mathbf{A}\mathbb{E}[\boldsymbol{z}_{t-1}^{\nu,{\rm T}}\boldsymbol{z}_t^\nu] + \mathbb{E}[\boldsymbol{z}_t^\nu\boldsymbol{z}_t^{\nu,{\rm T}}]\right)_{ii}\Bigg] + \cdots, \qquad \text{[S.24]}
\end{aligned}$$

where $(\cdots)_{ii}$ in the last line represents the ii component of the matrix inside the parentheses. The notation $|M|$ denotes the determinant of a matrix M.

The new matrix $\mathbf{A}^{\rm new}$ is obtained by minimizing the quantities in the second line of Eq. (S.24). Since $\mathbf{A}_{ij}$ and $\mathbf{A}_{i'j'}$ are decoupled if $i \neq i'$, each row of $\mathbf{A}^{\rm new}$ can be determined separately. Specifically, the i-th row $(A_{i1}^{\rm new}, \cdots, A_{iN_D}^{\rm new}) := \boldsymbol{\zeta}^\top$ is obtained by solving

$$\min_\zeta \frac{1}{2}\boldsymbol{\zeta}^\top\boldsymbol{g}\boldsymbol{\zeta} + \boldsymbol{q}_i^\top\boldsymbol{\zeta} \qquad \text{[S.25]}$$

under the constraints, $\sum_j \zeta_j = 1$ and $\zeta_j > 0$. The matrix $\boldsymbol{g}$ and the vector $\boldsymbol{q}_i$ are given by

$$\boldsymbol{g} = \sum_{t=1}^{T-1}\sum_\nu \frac{\mathbb{E}[\boldsymbol{z}_{t-1}^\nu\boldsymbol{z}_{t-1}^{\nu,\top}]}{H(\bar{x}_{i,t-1}^\nu)},$$
$$\boldsymbol{q}_i = \sum_{t=1}^{T-1}\sum_\nu \frac{\mathbb{E}[\boldsymbol{z}_{t,i}^\nu\boldsymbol{z}_{t-1}^{\nu,\top}]}{H(\bar{x}_{i,t-1}^\nu)}, \qquad \text{[S.26]}$$

which can be evaluated by using Eq. (S.23). We determine each row of $\mathbf{A}^{\rm new}$ by solving the constrained quadratic programming Eq. S.25 using the Python package `CVXOPT`.

After determining $\mathbf{A}^{\rm new}$, the new effective population size $N_{{\rm e},i}^{\rm new}$ is determined by differentiating Eq. (S.24) with respect to $N_{{\rm e},i}$. The result is

$$N_{{\rm eff},i}^{\rm new} = \frac{(T-1)N_{\rm lin}}{\sum_{t=1}^{T-1}\sum_{\nu=1}^{N_{\rm lin}}\frac{1}{H(\bar{x}_{i,t-1}^\nu)}\left(\mathbf{A}^{\rm new}\mathbb{E}[\boldsymbol{z}_{t-1}^\nu\boldsymbol{z}_{t-1}^{\nu,\top}](\mathbf{A}^{\rm new})^\top - \mathbb{E}[\boldsymbol{z}_t^\nu\boldsymbol{z}_{t-1}^{\nu,\top}](\mathbf{A}^{\rm new})^\top - \mathbf{A}^{\rm new}\mathbb{E}[\boldsymbol{z}_{t-1}^{\nu,\top}\boldsymbol{z}_t^\nu] + \mathbb{E}[\boldsymbol{z}_t^\nu\boldsymbol{z}_t^{\nu,\top}]\right)_{ii}}. \qquad \text{[S.27]}$$

Q also has the terms that are dependent on the parameter describing the deviation from uniform sampling, c_i;

$$\begin{aligned}
Q(\Theta, \Theta^{\rm old})|_{c_i-{\rm dep.}} &= -\frac{1}{2}\sum_{t=0}^{T-1}\sum_\nu \ln|\Sigma_t^\nu| - \mathbb{E}_{p(\boldsymbol{z}|\boldsymbol{x},\Theta^{\rm old})}\left[\frac{1}{2}\sum_{t=0}^{T-1}\sum_\nu(\boldsymbol{x}_t^\nu - \boldsymbol{z}_t^\nu)^\top(\Sigma_t^\nu)^{-1}(\boldsymbol{x}_t^\nu - \boldsymbol{z}_t^\nu)\right] \\
&= \sum_i\sum_\nu\left[-\frac{T}{2}\ln c_i - \frac{1}{2}\sum_{t=0}^{T-1}\frac{S_{i,t}}{c_iH(\bar{x}_{t,i}^\nu)}(x_{t,i}^\nu x_{t,i}^\nu - 2x_{t,i}^\nu\mathbb{E}[z_{t,i}^\nu] + \mathbb{E}[z_{t,i}^\nu z_{t,i}^\nu])\right]. \qquad \text{[S.28]}
\end{aligned}$$

*There is an erratum in the corresponding equation in ref. (11).

The new value of the measurement noise overdispersion c_i^{new} is determined by minimizing the above expression with respect to c_i (subjected to ≥ 1):

$$c_i^{\text{new}} = \max[1, \frac{1}{TN_{\text{lin}}} \sum_{\nu} \sum_{t=0}^{T-1} \frac{S_{i,t}}{H(\bar{x}_{t,i}^{\nu})} (x_{t,i}^{\nu} x_{t,i}^{\nu} - 2x_{t,i}^{\nu} \mathbb{E}[z_{t,i}^{\nu}] + \mathbb{E}[z_{t,i}^{\nu} z_{t,i}^{\nu}])]. \quad [\text{S.29}]$$

In the EM algorithm, we initialized the parameters, $\Theta = \Theta_0$, and iteratively updated them to Θ^{new}, by solving Eq. S.25 and evaluating Eqns. S.27 and S.29, until the likelihood stabilizes/converges.

Regularization: To stabilize the inference for deme-resolved analysis (higher than at region level), we added a Ridge-like regularization term $\Lambda \sum_i \sum_{j \neq i} A_{ij}^2$, to Q. Here, Λ is a regularization parameter. The value of Λ is determined via cross-validation by dividing the set of allele-frequency (or lineage-frequency) trajectories into training and test data of equal size.

Bootstrapping: To evaluate the uncertainty in the MLE, we performed bootstrapping by randomly sampling lineages (or independent alleles) with replacement to generate multiple new sets, each containing the same number of lineages as the original dataset. We then applied the EM algorithm to each of these generated sets.

S.2.3 Computational tests.

- 3-deme system We simulated frequency time-series data using the 3×3 importation-rate matrix shown in Fig. 2. The effective population size and sampling rate were set to values similar to those inferred for London and its neighboring regions during the Delta wave. The number of frequency trajectories generated was 73, matching the number of alleles used in the 3-deme analysis of London and its two neighboring regions during the Delta wave. The boxplots of the true and inferred parameter values (estimated using the MCMC method) are shown in Fig. 2D. The numerical values of the mean and interquartile range of the inferred matrix are shown in Fig. S8.

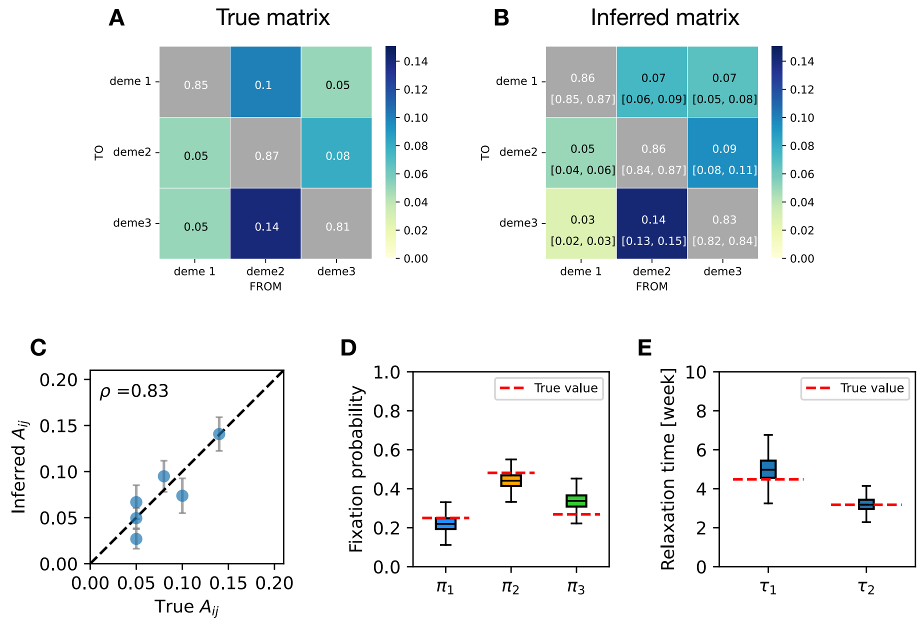

Fig. S8. Supplementary figures to Fig. 2. (**A**) The true matrix used for simulation. (**B**) The mean of the inferred matrix estimated using the MCMC method, with the interquartile range displayed for each matrix element. (**C**) Element-wise comparison between the true and posterior mean values of $\mathbf{A}_{ij}$ for off-diagonal elements. The Pearson correlation coefficient is 0.86. Error bars represent the standard deviations of the posterior values. (**D**) The inferred fixation probabilities. (**E**) The inferred relaxation times corresponding to the eigenvalues $|\lambda_1| > |\lambda_2|$ of the importation-rate matrix. In the boxplots, the boxes represent the IQR, with the central line indicating the median. The whiskers extend to the most extreme data points within 1.5 times the IQR from the first and third quartiles. The true values are indicated by red lines.

For the same parameter setting, we generated the time-series data of 20 and 60 alleles and applied the HMM-MCMC, HMM-EM, and least squares (LS; see Eq. 3) methods. The inferred results are compared in Figs. S9A-D. The LS method, which ignores fluctuations due to genetic drift and sampling noise, tends to overestimate the interaction strengths (Fig. S9C). Note that the results from the LS method are not displayed in Figs. S9C and D because the LS method cannot infer $N_{\text{e},i}$ and c_i.

- 50-deme system We simulated frequency time-series data using the 50×50 matrix shown in Fig. S10A, which was constructed by assuming five strongly interacting blocks, each consisting of 10 demes. The effective population size $N_{\text{e},i}$ and c_i were set to $N_{\text{e},i} = 1200$ and $c_i = 1.0$, respectively. The sampling rate was assumed to be $S_i = 500$ for all demes. These values were chosen to mimic the actual situation during the Delta wave in England. From the simulated data, we inferred the parameters using the HMM-EM and LS methods (Figs. S10B and S10C). Fig. S10D compares the matrix elements of the true $\mathbf{A}_{ij}$ with those of the inferred $\mathbf{A}_{ij}$. The LS method tends to overestimate interactions, especially for small couplings. This bias is further illustrated in Fig. S10E, which shows the histogram of the inferred matrix elements corresponding to the zero elements in the true matrix.

The bias in the LS method can be intuitively understood by considering that in a large noise limit, the LS solution converges to a homogeneous matrix $\mathbf{A}_{ij} = \frac{1}{N_D}$. Thus, using the LS method, small couplings (roughly, $\mathbf{A}_{ij} < \frac{1}{N_D}$) tend to be overestimated, while large couplings ($\mathbf{A}_{ij} > \frac{1}{N_D}$) tend to be underestimated.

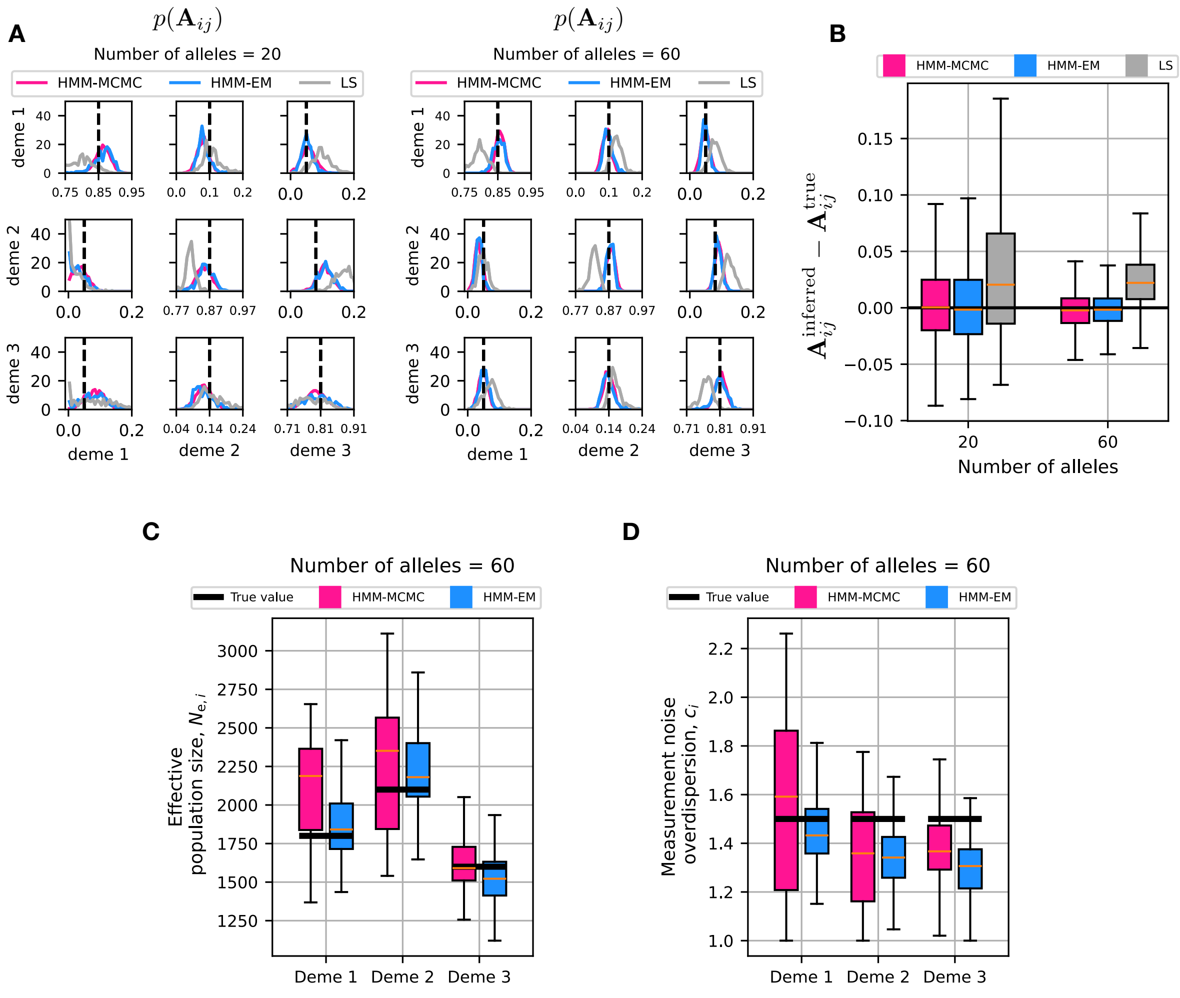

Fig. S9. The Wright-Fisher model on three demes is simulated, where the importation-rate matrix $\mathbf{A}$ and the effective population sizes are those shown in Fig. 2A. The measurement noise overdispersion parameter is set to $c_i = 1.5$. (**A**) The distribution of $\mathbf{A}$ inferred by the HMM-MCMC, HMM-EM, and LS methods, from 20 alleles (left) and 60 alleles (right). For the HMM-MCMC method, it represents the posterior distribution, while for the HMM-EM and LS methods, it represents the bootstrap distribution. (**B**) The error, $\mathbf{A}_{ij}^{\text{infer}} - \mathbf{A}_{ij}^{\text{true}}$, in the inference of interaction strengths for the HMM-MCMC, HMM-EM, and LS methods, where all pairs i, j are aggregated. (**C**) Boxplots showing the effective population sizes, inferred using the HMM-MCMC method from 60 alleles. The true values are indicated by thick horizontal lines. (**D**) Boxplots showing the measurement noise overdispersion, c_i, inferred using the HMM-MCMC method from 60 alleles. The true values ($c_i = 1.5$) are indicated by thick horizontal lines.

See also Fig. S28 for the effect of downsampling on inference from the actual data for the Delta variant in England.

S.2.4 Simulation of Figure 1A. In Fig. 1A, where the effect of couplings between demes on allele frequency dynamics is illustrated in a two-deme system, we used the following parameters: The importation-rate matrix during the 'lockdown' period ($0 \leq t < 20$) is the identity matrix, and the one after lifting lockdown ($20 \leq t \geq 45$) is given by

$$\mathbf{A} = \begin{pmatrix} 0.9 & 0.1 \\ 0.25 & 0.7 \end{pmatrix}. \qquad \text{[S.30]}$$

The effective population size is $N_{\mathrm{e},i} = 300$ for $i = 1, 2$, while the sampling size is set to $S_{i,t} = 10^4$. For illustrative purposes, we set the measurement error to be negligibly small compared to the genetic drift. The initial frequencies are set to $f_1(t = 0) = 0.9$ and $f_2(t = 0) = 0.1$. After a long-time, the frequencies in the two demes converge to the steady state given by $f_i = \frac{A_{12}\times f_1 + A_{21}\times f_2}{A_{12}+A_{21}} = \frac{0.1\times 0.9 + 0.25\times 0.1}{0.1+0.25} \approx 0.33$.

S.2.5 Effects of estimation errors in matrix elements $\mathbf{A}_{ij}$ on eigenvalues and eigenvectors. Here, we analyze how entrywise perturbations $\delta\mathbf{A}_{ij}$ influence the eigenvalues and eigenvectors of $\mathbf{A}$, and show that, to leading order, the errors in the eigenvalues/vectors depend on the absolute errors $\delta\mathbf{A}_{ij}$, not on the relative errors $\delta\mathbf{A}_{ij}/\mathbf{A}_{ij}$.

We consider a perturbed matrix $\mathbf{A}' = \mathbf{A} + \delta\mathbf{A}$, where $\delta\mathbf{A}_{ij} \ll 1$ and $\sum_j \delta\mathbf{A}_{ij} = 0$. Denote the eigenvalues and the right/left eigenvectors of the unperturbed matrix $\mathbf{A}$ by

$$\mathbf{A}\,|i^{(0)}\rangle = \lambda_i^{(0)}\,|i^{(0)}\rangle, \qquad \text{[S.31]}$$

$$\langle i^{(0)}|\,\mathbf{A} = \lambda_i^{(0)}\,\langle i^{(0)}|, \qquad \text{[S.32]}$$

for $i = 0, \ldots, n-1$ (n is the number of demes), with $\langle i^{(0)}|j^{(0)}\rangle = \delta_{ij}$. We assume all the eigenvalues are nondegenerate.

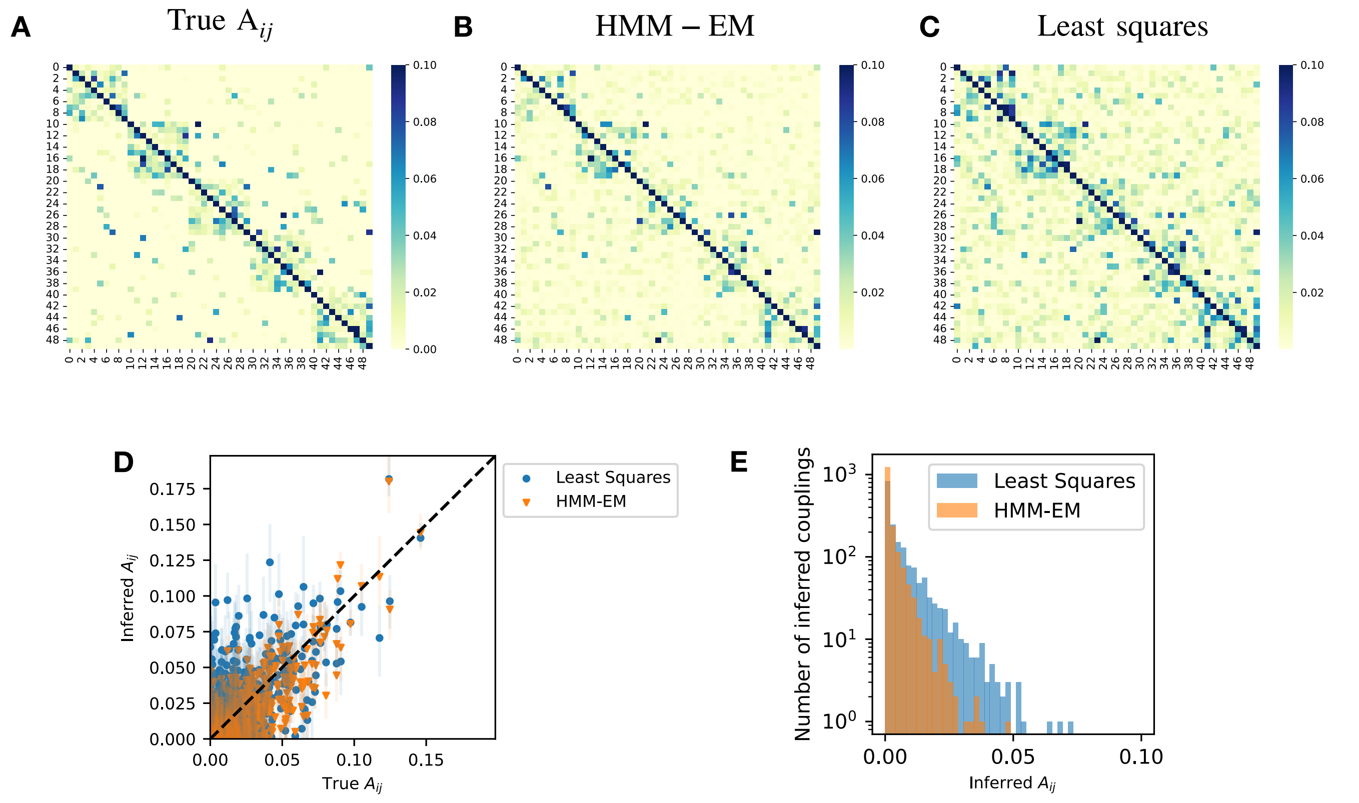

Fig. S10. (**A**) Heatmap showing the true importation-rate matrix. All matrix elements within the five diagonal blocks (each consisting of 10 demes) are assumed to be nonzero, while the elements of the off-diagonal blocks are nonzero with probability of 0.1. The nonzero elements are randomly sampled from the exponential distribution with a mean of 0.02. (**B**) The matrix inferred from the HMM-EM method. (**C**) The matrix inferred from the LS method. (**D**) Element-wise comparison between the true matrix and the inferred matrix obtained using the least squares and HMM-EM methods. (**E**) Histogram of the inferred matrix elements corresponding to zeros in the true matrix.

For the perturbed matrix, we write the eigenvalues and eigenvectors as

$$\Big(\mathbf{A}+\delta\mathbf{A}\Big)\Big(|i^{(0)}\rangle+|i^{(1)}\rangle\Big)=\Big(\lambda_i^{(0)}+\delta\lambda_i\Big)\Big(|i^{(0)}\rangle+|i^{(1)}\rangle\Big), \tag{S.33}$$

$$\Big(\langle i^{(0)}|+\langle i^{(1)}|\Big)\Big(\mathbf{A}+\delta\mathbf{A}\Big)=\Big(\lambda_i^{(0)}+\delta\lambda_i\Big)\Big(\langle i^{(0)}|+\langle i^{(1)}|\Big). \tag{S.34}$$

Collecting the first-order terms gives

$$\Big(\mathbf{A}-\lambda_i^{(0)}I\Big)\,|i^{(1)}\rangle=\Big(\delta\lambda_i\,I-\delta\mathbf{A}\Big)\,|i^{(0)}\rangle, \tag{S.35}$$

$$\langle i^{(1)}|\Big(\mathbf{A}-\lambda_i^{(0)}I\Big)=\langle i^{(0)}|\Big(\delta\lambda_i\,I-\delta\mathbf{A}\Big). \tag{S.36}$$

Left-multiplying the first equation by $\langle i^{(0)}|$ (or right-multiplying the second by $|i^{(0)}\rangle$) yields the first-order eigenvalue shift,

$$\delta\lambda_i=\langle i^{(0)}|\,\delta\mathbf{A}\,|i^{(0)}\rangle. \tag{S.37}$$

Since $\{|j^{(0)}\rangle\}_{j=0}^{n-1}$ is a basis, we can expand

$$|i^{(1)}\rangle=\sum_{j=1}^{n}c_{ij}\,|j^{(0)}\rangle. \tag{S.38}$$

If $|i^{(1)}\rangle$ solves Eq. S.35, then so does $|i^{(1)}\rangle+\alpha\,|i^{(0)}\rangle$ for any scalar α. Using this gauge freedom, we may assume $c_{ii}=0$. Hence,

$$|i^{(1)}\rangle=\sum_{j\neq i}c_{ij}\,|j^{(0)}\rangle. \tag{S.39}$$

Substituting S.39 into S.35 gives

$$\sum_{j\neq i}c_{ij}\,(\lambda_j^{(0)}-\lambda_i^{(0)})\,|j^{(0)}\rangle=\Big(\delta\lambda_i\,I-\delta\mathbf{A}\Big)\,|i^{(0)}\rangle. \tag{S.40}$$

Projecting with $\langle k^{(0)}|$ for $k\neq i$ yields

$$c_{ik}\,(\lambda_k^{(0)}-\lambda_i^{(0)})\;=\;-\,\langle k^{(0)}|\,\delta\mathbf{A}\,|i^{(0)}\rangle, \tag{S.41}$$

hence

$$c_{ik} = \frac{\langle k^{(0)} | \, \delta\mathbf{A} \, | i^{(0)} \rangle}{\lambda_i^{(0)} - \lambda_k^{(0)}} \qquad (k \neq i). \tag{S.42}$$

Therefore, the first-order change in the right eigenvector is

$$| i^{(1)} \rangle = \sum_{j \neq i} \frac{\langle j^{(0)} | \, \delta\mathbf{A} \, | i^{(0)} \rangle}{\lambda_i^{(0)} - \lambda_j^{(0)}} \, | j^{(0)} \rangle. \tag{S.43}$$

Similarly, the first-order change in the left eigenvector is

$$\langle i^{(1)} | = \sum_{j \neq i} \frac{\langle i^{(0)} | \, \delta\mathbf{A} \, | j^{(0)} \rangle}{\lambda_i^{(0)} - \lambda_j^{(0)}} \, \langle j^{(0)} |. \tag{S.44}$$

From an inference perspective, Eqs. S.37, S.43, and S.44 indicate that uncertainty in the estimated eigenvalues and eigenvectors is primarily driven by the *absolute estimation error* $\delta\mathbf{A}$ in the matrix (with weights set by the spectral information), rather than by the componentwise relative errors $\delta\mathbf{A}_{ij}/\mathbf{A}_{ij}$. For example, Eq. S.37 implies that the relative error of the i-th eigenvalue is $\frac{\delta\lambda_i}{\lambda_i^{(0)}} = \frac{\langle i^{(0)} | \, \delta\mathbf{A} \, | i^{(0)} \rangle}{\lambda_i^{(0)}}$. Consequently, even if some relative errors in the matrix elements $\mathbf{A}_{ij}$ are large, accurate inference of eigenvalues and eigenvectors can still be possible, provided the error in relevant matrix elements $\langle i^{(0)} | \, \delta\mathbf{A} \, | j^{(0)} \rangle$ is small. See Fig. S11 for a demonstration using simulated data.

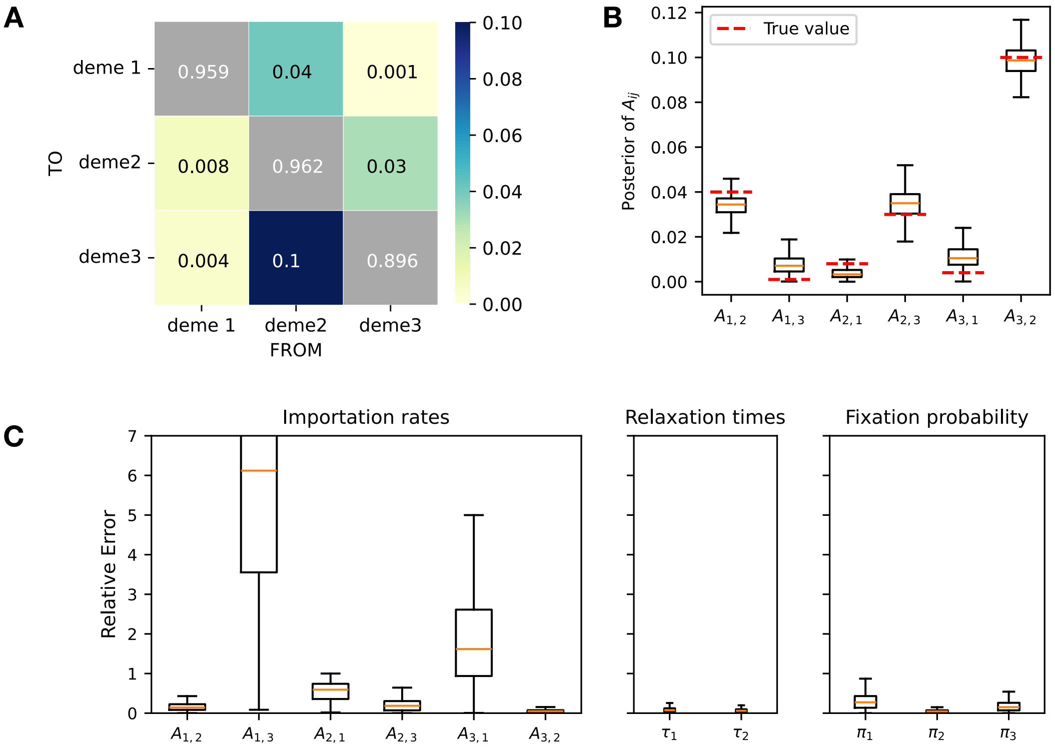

Fig. S11. Effects of estimation error in $\mathbf{A}_{ij}$ on predictions of relaxation dynamics using simulated data. Simulations are performed using the matrix shown in Panel (**A**). To assess the impact of large *relative* errors arising from small importation rates, we set a subset of matrix entries to small values. The effective population sizes, weekly sequence counts, and measurement-noise parameters are fixed at $N_{\mathrm{e},i} = 5000$, $S_i = 5000$, and $c_i = 1$. (**B**) Posterior distributions of the matrix elements $\mathbf{A}_{ij}$ inferred by the HMM method. (**C**) Posterior distributions of the *relative* error $\frac{|O^{\mathrm{inferred}} - O^{\mathrm{true}}|}{|O^{\mathrm{true}}|}$, where O denotes either a matrix element $\mathbf{A}_{ij}$, a relaxation time $\tau_i \equiv -1/\log|\lambda_i|$, or a fixation probability π_i (components of the leading left eigenvector $\langle 0|$). Although $\mathbf{A}_{ij}$ entries with small true values exhibit large *relative* estimation errors, the relaxation timescales and fixation probabilities are estimated with substantially higher accuracy, consistent with the prediction that eigenpair errors are governed by the absolute error $\delta\mathbf{A}$ rather than by componentwise relative errors.

S.3 Reproductive values in England

In the main text, we showed that the per-capita reproductive values π_i/I_i vary across regions (Fig. 7C). Similarly, the unnormalized "class" reproductive values π_i are spatially heterogeneous, as shwon in Fig. S12. In this section, we provide supplementary analyses of the observed heterogeneity in π_i.

S.3.1 Ranking regions according to ratios of bidirectional importation rates.

As discussed in the main text, reproductive values predict ratios of bidirectional importation rates when the principle of detailed balance, Eq. 2, holds. To verify this, we simultaneously reordered both rows and columns of the matrix $\mathbf{A}_{ij}$ to maximize the asymmetry, as measured by $\sum i > j\mathbf{A}_{ij}/(\mathbf{A}_{ij} + \mathbf{A}_{ji})$. This approach indeed arranges regions roughly in line with the values of π_i for both the Alpha and Delta waves, see Fig. S13. In particular, the two regions, EE and SW, which have lower π_i values, are ordered last in the reordered matrices.

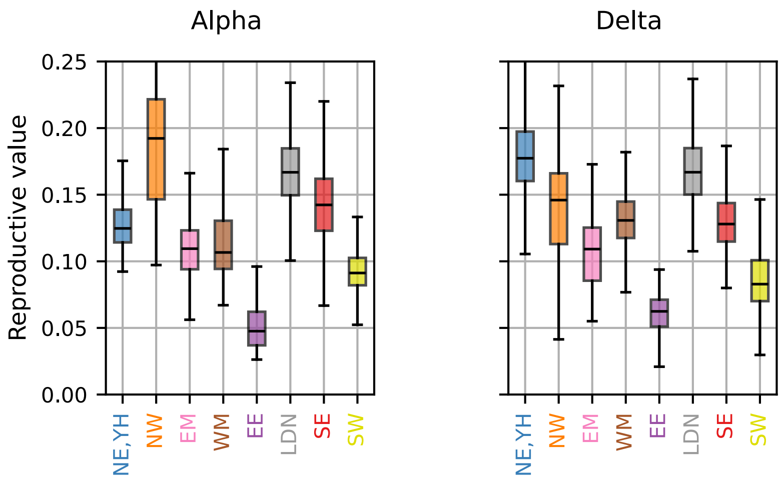

Fig. S12. The inferred class reproductive values for each region during the Alpha wave (epiweeks 57-68) and Delta wave (epiweeks 82-95) in England. In the boxplots, the boxes represent the interquartile range (Q1 to Q3), with the central line indicating the median. The whiskers extend to $Q_1 - 1.5 \times \mathrm{IQR}$ and $Q_3 + 1.5 \times \mathrm{IQR}$.

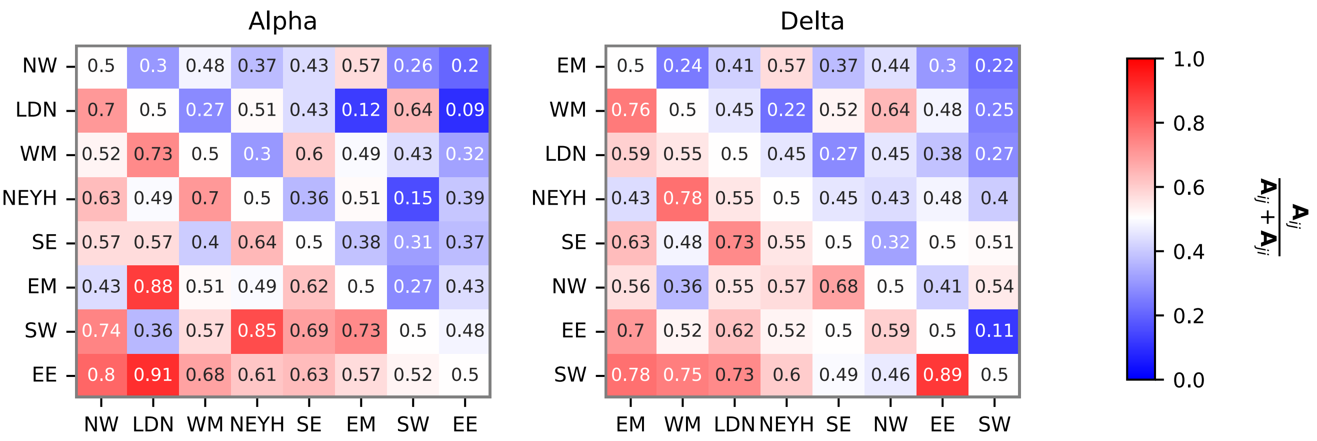

Fig. S13. Using the inferred importation-rate matrix $\mathbf{A}$ for the Alpha and Delta variants in England and the number of infected NIi, as measured by the COVID-19 Infection Survey (13), the values of $\mathbf{A}_{ij}/(\mathbf{A}_{ij} + \mathbf{A}_{ji})$ are represented by heat maps. The order of regions is arranged to maximize the asymmetry, $\sum_{i>j} \mathbf{A}_{ij}/(\mathbf{A}_{ij} + \mathbf{A}_{ji})$, is maximized.

S.3.2 Testing the effect of the delay in sequence reporting.

One of the possible reasons for the observed heterogeneity in the reproductive value π_i is the difference in the population size. In fact, π_i correlates with the average number of infected individuals in each region during the wave; the Spearman correlation coefficients are $\rho = 0.81$ (p-value 0.015) for the Alpha wave and $\rho = 0.88$ (p-value 0.004) for the Delta wave, respectively. However, as presented in Fig. 7, the reproductive values are heterogeneous even after normalizing by the number of infected individuals.

Another possible explanation could be that the inferred heterogeneity in π_i results from artifacts caused by variations in the timing of disease reporting. For instance, assume two regions i and j have perfectly symmetrical cross-importation rates. If region i reports cases substantially later than region j, its frequency trajectories will tend to follow region j, mimicking a causal influence of j on i. Consequently,

the importation rate from j to i, $\mathbf{A}_{ij}$, is likely to be overestimated, which would then expected to decrease π_i. A more quantitative argument based on perturbative analysis is provided in Sec. S.3.3.

While no systematic reporting time heterogeneity has been documented to our knowledge, to test the impact of a hypothetical reporting delay, we artificially advanced the allele counts data from EE by $\tau_{\mathrm{shift}} (= 1, 2)$ weeks compared to the other regions, mimicking the scenario where the other regions report sequences later by τ_{shift} weeks. We then re-inferred the importation-rate matrix (Fig. S14). As expected, the reproductive value of EE increases as τ_{shift} is increased, highlighting the importance of accurate reporting dates for precise inference.

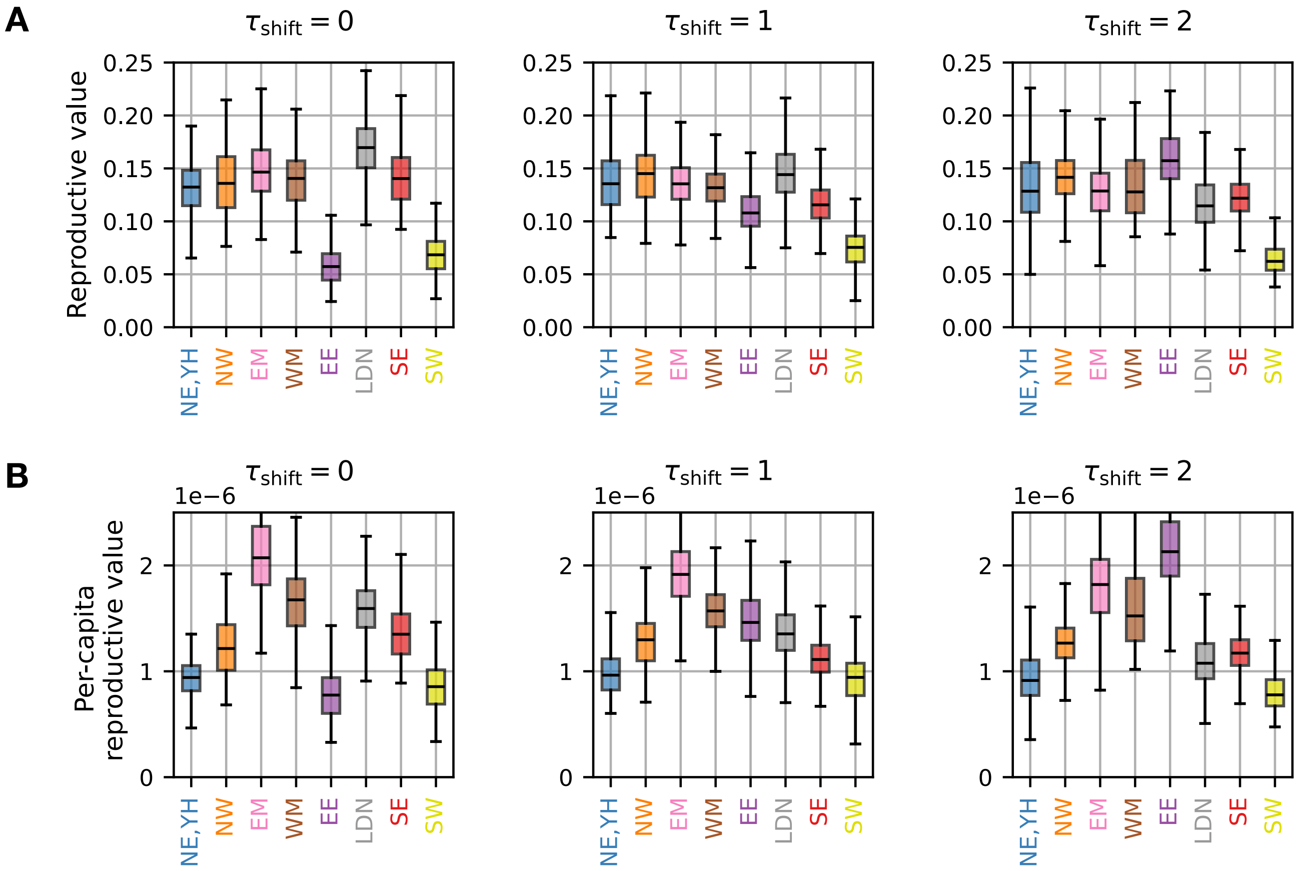

Fig. S14. (**A**) The reproductive values inferred for the data during the Delta wave in England (epiweek 82+τ_{shift} to 93+τ_{shift} for EE and epiweek 84 to 94 for the other regions), where sequence reporting dates from EE are artificially advanced by τ_{shift} weeks. (**B**) The inferred per-capita reproductive values. In the boxplots, the boxes represent the interquartile range (Q1 to Q3), with the central line indicating the median. The whiskers extend to $Q_1 - 1.5 \times \mathrm{IQR}$ and $Q_3 + 1.5 \times \mathrm{IQR}$.

S.3.3 Perturbative analysis of the effect of delay in reporting.

Consider a population consisting of two groups, each containing N_{D_1} and N_{D_2} demes. Assume a consistent delay of one week in sequence reporting from the demes in the second group. Ignoring the genetic drift and measurement noise, the dynamics of the allele frequencies, $\boldsymbol{z}_1(t) \in \mathbb{R}^{N_{D_1}}$ in the N_{D_1} demes and $\boldsymbol{z}_2(t) \in \mathbb{R}^{N_{D_2}}$ in the N_{D_2} demes, are described by

$$\begin{pmatrix} \boldsymbol{z}_1(t+1) \\ \boldsymbol{z}_2(t+1) \end{pmatrix} = \mathbf{A} \begin{pmatrix} \boldsymbol{z}_1(t) \\ \boldsymbol{z}_2(t) \end{pmatrix} = \begin{pmatrix} \mathbf{A}^{11} & \mathbf{A}^{12} \\ \mathbf{A}^{21} & \mathbf{A}^{22} \end{pmatrix} \begin{pmatrix} \boldsymbol{z}_1(t) \\ \boldsymbol{z}_2(t) \end{pmatrix}, \tag{S.45}$$

where $\mathbf{A}^{IJ} (I, J = 1, 2)$ represents the matrix of importation rates from demes in J to demes in I. The observed frequencies, $\boldsymbol{x}_1(t) \in \mathbb{R}^{N_{D_1}}$ and $\boldsymbol{x}_2(t) \in \mathbb{R}^{N_{D_2}}$, are given by

$$\begin{pmatrix} \boldsymbol{x}_1(t) \\ \boldsymbol{x}_2(t) \end{pmatrix} = \begin{pmatrix} \boldsymbol{z}_1(t) \\ \boldsymbol{z}_2(t-1) \end{pmatrix}. \tag{S.46}$$

The dynamics of the observed frequencies are written as

$$\begin{aligned} \begin{pmatrix} \boldsymbol{x}_1(t+1) \\ \boldsymbol{x}_2(t+1) \end{pmatrix} &= \begin{pmatrix} \boldsymbol{z}_1(t+1) \\ \boldsymbol{z}_2(t) \end{pmatrix} \\ &= \begin{pmatrix} \mathbf{A}^{11}\boldsymbol{z}_1(t) + \mathbf{A}^{12}\boldsymbol{z}_2(t) \\ \mathbf{A}^{21}\boldsymbol{z}_1(t-1) + \mathbf{A}^{22}\boldsymbol{z}_2(t-1) \end{pmatrix} \\ &= \begin{pmatrix} \mathbf{A}^{11}\boldsymbol{z}_1(t) + \mathbf{A}^{12}\left(\mathbf{A}^{21}\boldsymbol{z}_1(t-1) + \mathbf{A}^{22}\boldsymbol{z}_2(t-1)\right) \\ \mathbf{A}^{21}\left((\mathbf{A}^{-1})^{11}\boldsymbol{z}_1(t) + (\mathbf{A}^{-1})^{12}\boldsymbol{z}_2(t)\right) + \mathbf{A}^{22}\boldsymbol{z}_2(t-1) \end{pmatrix}, \end{aligned} \tag{S.47}$$

where $(\mathbf{A}^{-1})^{11} \in \mathbb{R}^{N_{D_1} \times N_{D_1}}$ and $(\mathbf{A}^{-1})^{12} \in \mathbb{R}^{N_{D_2} \times N_{D_2}}$ are the block matrices of $\mathbf{A}^{-1}$, defined by $\mathbf{A}^{-1} = \begin{pmatrix} (\mathbf{A}^{-1})^{11} & (\mathbf{A}^{-1})^{12} \\ (\mathbf{A}^{-1})^{21} & (\mathbf{A}^{-1})^{22} \end{pmatrix}$. We assume that the interactions are weak, i.e. $\mathbf{A}_{ij} = \mathcal{O}(\epsilon)$ $(\epsilon \ll 1)$ for $i \neq j$, which implies $\boldsymbol{z}_1(t-1) = \boldsymbol{z}_1(t) + \mathcal{O}(\epsilon)$ and $\boldsymbol{z}_2(t) = \boldsymbol{z}_2(t-1) + \mathcal{O}(\epsilon)$. Under this approximation, Eq. (S.47) can be written as

$$\begin{aligned} \begin{pmatrix} \boldsymbol{x}_1(t+1) \\ \boldsymbol{x}_2(t+1) \end{pmatrix} &= \begin{pmatrix} \mathbf{A}^{11} + \mathbf{A}^{12}\mathbf{A}^{21} & \mathbf{A}^{12}\mathbf{A}^{22} \\ \mathbf{A}^{21}(\mathbf{A}^{-1})^{11} & \mathbf{A}^{21}(\mathbf{A}^{-1})^{12} + \mathbf{A}^{22} \end{pmatrix} \begin{pmatrix} \boldsymbol{z}_1(t) \\ \boldsymbol{z}_2(t-1) \end{pmatrix} + \mathcal{O}(\epsilon^3) \\ &= \underbrace{\begin{pmatrix} \mathbf{A}^{11} + \mathbf{A}^{12}\mathbf{A}^{21} & \mathbf{A}^{12}\mathbf{A}^{22} \\ \mathbf{A}^{21}(\mathbf{A}^{-1})^{11} & \mathbf{A}^{21}(\mathbf{A}^{-1})^{12} + \mathbf{A}^{22} \end{pmatrix}}_{\equiv \mathbf{A}_{\text{delay}}} \begin{pmatrix} \boldsymbol{x}_1(t) \\ \boldsymbol{x}_2(t) \end{pmatrix} + \mathcal{O}(\epsilon^3). \end{aligned} \qquad \text{[S.48]}$$

Hence, under the delay from the second group, the apparent dynamics are still linear but described by the modified matrix $\mathbf{A}_{\text{delay}}$. Compared to the true matrix $\mathbf{A} = \begin{pmatrix} \mathbf{A}^{11} & \mathbf{A}^{12} \\ \mathbf{A}^{21} & \mathbf{A}^{22} \end{pmatrix}$, the delay fictitiously modifies the upper-right block (representing the influence of the second group on the first group) by introducing the factor $\mathbf{A}^{22}$ and the lower-left block (representing the influence of the first group on the second group) by introducing the factor $(\mathbf{A}^{-1})^{11}$.

To illustrate the delay effect, let us consider the simplest case where $N_{D_1} = N_{D_2} = 1$, where $\mathbf{A}^{11}, \mathbf{A}^{12}, \mathbf{A}^{21}, \mathbf{A}^{22}$ are not matrices but positive scalars. The constraints $\mathbf{A}^{11} + \mathbf{A}^{12} = 1$ and $\mathbf{A}^{21} + \mathbf{A}^{22} = 1$, along with the positivity condition, imply that the delay-induced factors satisfy $\mathbf{A}^{22} < 1$ and $(\mathbf{A}^{-1})^{11} > 1$. Therefore, when the second group's report is delayed, the influence of the second group on the first group becomes underestimated, whereas the influence of the first group on the second group becomes overestimated. This effect is demonstrated for cases where $N_{D_1} = 21$ and $N_{D_2} = 2$ in Fig. S15.

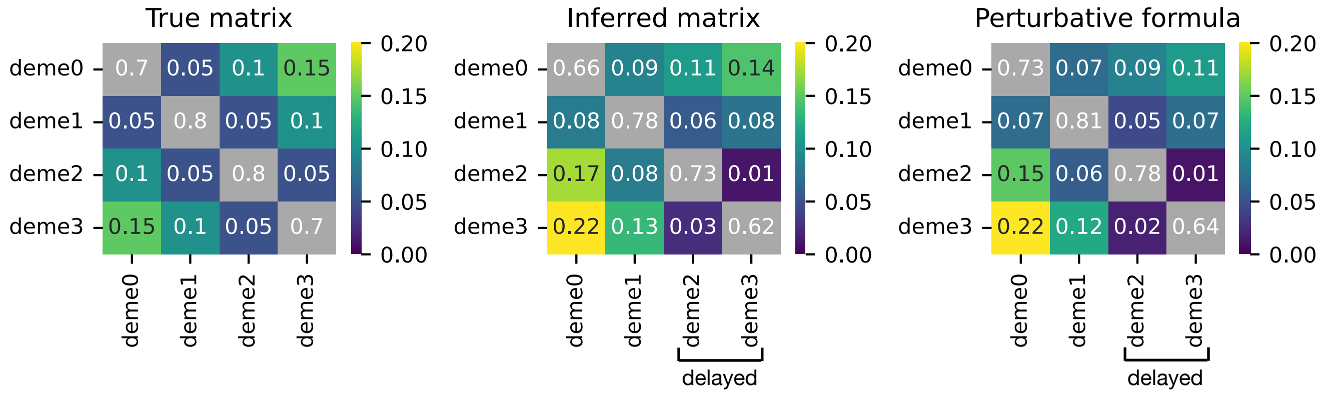

Fig. S15. The Wright-Fisher simulation was performed with the 4×4 importation-rate matrix shown in the leftmost heatmap. The effective population sizes and sampling rates were set to $N_{\mathrm{e},i} = 10^4$ and $S_i = 10^4$, respectively, for all demes. We examined a scenario in which there was a 1-week delay in sequence counts from deme 2 and deme 3. The matrices inferred from the HMM method and the perturbative formula in Eq. S.48 are presented in the middle and rightmost heatmaps, respectively. Consistent with the formula, the influences from demes 2 and 3 (corresponding to the third and fourth columns) are underestimated due to the delay, while those from demes 1 and 2 (corresponding to the first and second columns) are overestimated.

S.4 Spatial Resolution and Inference Reliability

In the deme-resolved results presented in Fig. 3, we divided England into 50 demes. In this section, we present two arguments supporting the idea that increasing the spatial resolution much beyond 50 demes is not feasible.

The first argument considers how $\mathbf{A}_{ij}$ should behave under changes in spatial resolution. Let the labels i and j represent demes, and let I and J represent coarse-grained areas, referred to as regions. Suppose we have a deme-level matrix $\mathbf{A}_{ij}$. If all demes within the same region interact strongly, the region-level interaction $\mathbf{A}_{IJ}$ can be approximated by:

$$\mathbf{A}_{IJ} \approx \sum_{i \in I} \sum_{j \in J} \frac{\pi_i}{\sum_{i' \in I} \pi_{i'}} \mathbf{A}_{ij}, \qquad \text{[S.49]}$$

where π_i is the reproductive value(the normalized left eigenvector with eigenvalue 1 of the matrix $\mathbf{A}_{ij}$). This formula can be understood from a time-backward perspective: assuming demes within the same region equilibrate instantaneously, a lineage in region I is distributed across demes $i \in$ region I with probability $\frac{\pi_i}{\sum_{j \in I} \pi_j}$. The lineage in deme $i \in$ region I then moves backward in time to deme j with probability $\mathbf{A}_{ij}$. Summing over demes $i \in$ region I and $j \in$ region J gives the probability that a lineage in region I moves backward in time to region J, which corresponds to $\mathbf{A}_{IJ}$ in Eq. (S.49).

In Fig. S16A and B, we show the deme-resolved matrix with $N_D = 50$ for the Delta wave in England and its coarse-grained counterpart. As shown in Fig. S16C, the coarse-grained matrix closely matches the one directly inferred from the region-level allele frequency data. Fig. S16D shows the correlation between the two matrices as a function of the number of demes N_D. The decline in correlation above $N_D \approx 70$ indicates a loss of inference reliability at higher spatial resolutions.

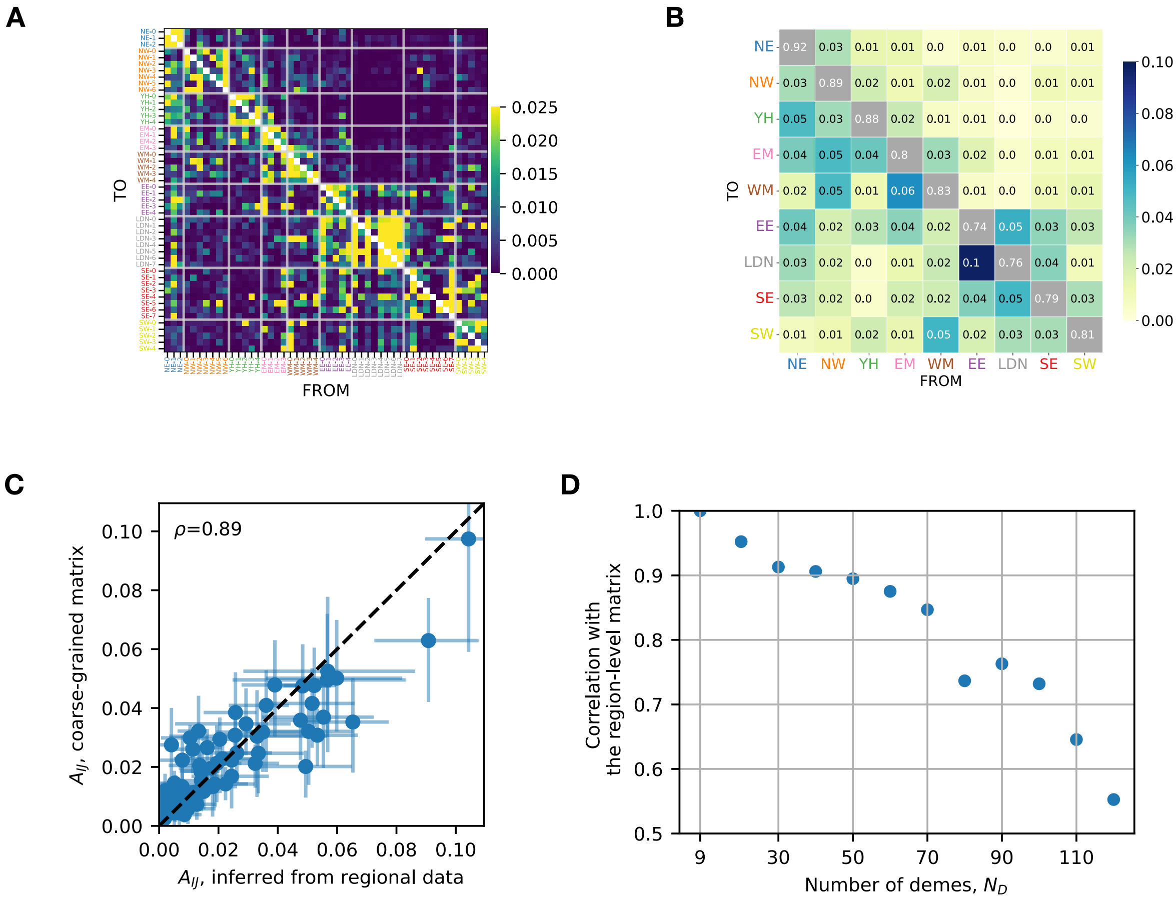

Fig. S16. (**A**) Heatmap showing the importation-rate matrix $\mathbf{A}$, with $N_D = 50$, during the Delta wave in England.(**B**) The coarse-grained matrix obtained by applying Eq. S.49 to the 50×50 matrix in Fig. **A**. (**C**) Element-wise comparison between the coarse-grained matrix and the matrix directly inferred from the region-level mutation data. The Pearson correlation coefficient is 0.89. (**D**) Plot showing the Pearson correlation between the 9×9 coarse-grained matrix, obtained from $N_D \times N_D$ deme-resolved matrix, and the 9×9 matrix directly inferred from the region-level mutation data, as a function of the number of demes N_D. For each spatial resolution N_D, the matrix elements between the two matrices are compared as in Fig. **C**.

Another indirect argument comes from the relaxation times of the eigenmodes. Fig. S17A displays the inferred importation-rate matrix for the Delta wave at the level of upper tier local authorities (UTLA), which is the finest spatial resolution available from our data. Fig. S17B shows the relaxation time, $-\ln|\lambda_i|$, for each eigenmode ($|\lambda_1| \leq |\lambda_2| \leq \ldots$). While the relaxation time around $i = 50$ is approximately 2 weeks, it decreases to less than 1 week for $i > 60$. Given that the unit time of our analysis is 1 week, modes with relaxation times shorter than 1 week cannot be reliably inferred. This observation suggests that significantly increasing the spatial resolution beyond 50 demes is not practical.

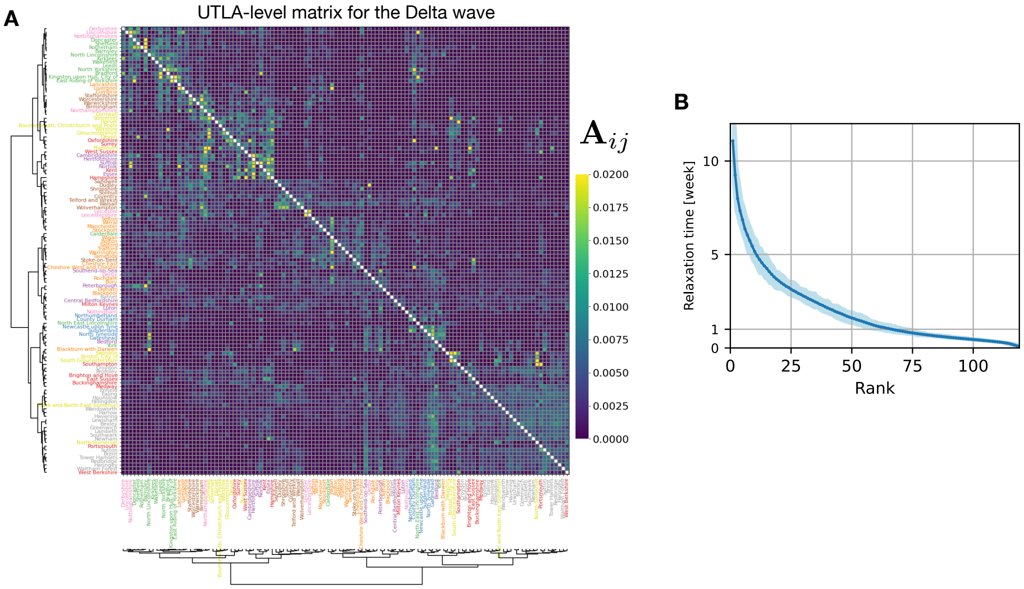

Fig. S17. (**A**) The importation-rate matrix among 120 UTLAs in England, inferred for the Delta wave (Jun 20, 2021 - Sep 25, 2021) with the HMM-EM method (without Ridge regularization). The top 120 UTLAs with the most sequences are used in this inference. The 120 UTLAs are ordered by performing hierarchical clustering with the Jensen-Shannon distance between rows. (**B**) The relaxation time for each mode of the importation-rate matrix. The shaded region shows the 95% confidence interval, computed using the bootstrapping method.

S.5 Detailed balance condition

The inferred infection matrices are typically asymmetrical, which can be checked, for example, for the Delta wave matrix shown in Fig. 3**A**. However, one might wonder if a more general symmetry, known as detailed balance, is maintained, which is often assumed in modeling and inference studies. Detailed balance is satisfied when the flux of lineages between two regions is balanced when traced backward in time. This implies that the backward-time lineage jump process is time-reversible. Mathematically, this detailed balance condition can be expressed as

$$\pi_i \mathbf{A}_{ij} = \pi_j \mathbf{A}_{ji} \ , \qquad \text{[S.50]}$$

meaning that, in equilibrium, the lineage flux from region j to i is equal to the reverse flux. In Bayesian phylodynamic inference, detailed balance is often assumed to simplify the learning algorithms (14). Moreover, as shown in Sec. S.6, standard epidemiological models imply detailed balance—without it, the lineage jump process would exhibit unwarranted cyclic dynamics in equilibrium.

It is therefore important to check whether the data justifies the detailed balance premise. We focus on the nine regions in England and plot the ratio of left and right-hand sides in Eq. S.50 for all possible pairwise interactions (Fig. S18). While most long-distance interactions are too weak to test for detailed balance, the strong neighbor-neighbor interactions are largely consistent with detailed balance.

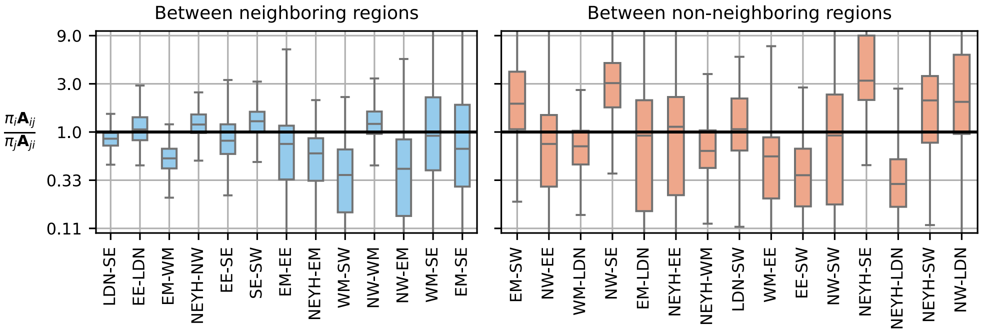

Fig. S18. Testing the detailed balance assumption. Under detailed balance, the ratio $\frac{\pi_i \mathbf{A}_{ij}}{\pi_j \mathbf{A}_{ji}}$ should equal one for all deme pairs (i, j), where π_i denotes the class reproductive value (satisfying $\sum_i \pi_i \mathbf{A}_{ij} = \pi_j$ and normalized to 1). The box plots show these ratios for all pairs of regions in England, where YH and NE are combined to a single region, using the 8×8 importation-rate matrix $\mathbf{A}_{ij}$ inferred from the region-level data of mutations during the Delta wave period (June 20, 2021 - October 31, 2021). Results for neighboring region pairs, which generally exhibit stronger couplings, are shown separately from those for non-neighboring regions, where couplings are typically smaller and exhibit greater inference error in $\frac{\pi_i \mathbf{A}_{ij}}{\pi_j \mathbf{A}_{ji}}$. In the boxplots, the boxes represent the interquartile range (Q1 to Q3), with the central line indicating the median. The whiskers extend to $Q_1 - 1.5 \times \mathrm{IQR}$ and $Q_3 + 1.5 \times \mathrm{IQR}$.

S.6 Epidemiological interpretation of $\mathbf{A}$

To aid in interpreting our importation-rate matrix $\mathbf{A}$, we derive its expressions using specific epidemiological models.

We begin by extending the multi-patch SIR model (10, 15–17) to include distinct multiple viral lineages. The extended model is represented by the following set of equations:

$$\dot{S}_i(t) = -S_i \sum_\mu \sum_{j=1}^{N_D} \beta_{ij}^\mu \frac{I_j^\mu(t)}{N_j} \quad \text{[S.51]}$$

$$\dot{I}_i^\mu(t) = S_i \sum_{j=1}^{N_D} \beta_{ij}^\mu \frac{I_j^\mu(t)}{N_j} - \gamma^\mu I_i^\mu(t) \quad \text{[S.52]}$$

$$\dot{R}_i(t) = \sum_\mu \gamma^\mu I_i^\mu(t). \quad \text{[S.53]}$$

Here, I_i^μ denotes the number of individuals infected by lineage μ in deme i, N_i is the population size, $N_i = S_i(t) + I_i(t) + R_i(t)$, and β_{ij}^μ is the transmission rate of lineage μ from an infected individual in deme j to susceptible individuals in deme i. γ^μ represents a lineage-specific recovery rate.

In terms of the total number of infected individuals $I_i \equiv \sum_\mu I_i^\mu$ in deme i and the frequency $f_i^\mu \equiv I_i^\mu / I_i$ of lineage μ, Eq. (S.52) is expressed as

$$\dot{f}_i^\mu = S_i \sum_j \frac{\beta_{ij}^\mu}{N_j} \frac{I_j}{I_i} f_j^\mu - S_i \sum_j \frac{\bar{\beta}_{ij}}{N_j} \frac{I_j}{I_i} f_i^\mu - (\gamma^\mu - \bar{\gamma}_i) f_i^\mu,$$

where $\bar{\beta}_{ij}$ and $\bar{\gamma}_i$ are lineage-averaged rates defined as

$$\bar{\beta}_{ij} \equiv \sum_\mu \beta_{ij}^\mu f_j^\mu \quad \text{[S.54]}$$

$$\bar{\gamma}_i \equiv \sum_\mu \gamma^\mu f_i^\mu. \quad \text{[S.55]}$$

In terms of the fractions $s_i \equiv \frac{S_i}{N_i}$ and $i_i \equiv \frac{I_i}{N_i}$, we have

$$\dot{f}_i^\mu = s_i \sum_j \frac{i_j}{i_i} (\beta_{ij}^\mu f_j^\mu - \bar{\beta}_{ij} f_i^\mu) - (\gamma^\mu - \bar{\gamma}_i) f_i^\mu. \quad \text{[S.56]}$$

S.6.1 Neutral case.

Suppose that all lineages have the same transmission rate and recovery rate, $\beta_{ij}^\mu = \beta_{ij}$ and $\gamma^\mu = \gamma$. Then, Eq. (S.56) reduces to

$$\dot{f}_i^\mu = \sum_{j \neq i} \mathbf{A}_{ij} (f_j^\mu - f_i^\mu). \quad \text{[S.57]}$$

where

$$\mathbf{A}_{ij} \equiv s_i \beta_{ij} \frac{i_j}{i_i}. \quad \text{[S.58]}$$

If we assume that $s_i \approx 1$, which is a reasonable approximation in realistic situations, and that the infected fraction is spatially homogeneous ($i_i \approx i_j$), as was the case during the plateau period of the Delta wave in England (Fig. S33), then $\mathbf{A}_{ij}$ can be approximated as $\mathbf{A}_{ij} \approx \beta_{ij}$.

By discretizing time using one week as the unit of time,

$$\begin{aligned} f_i^\mu(t+1) &= f_i^\mu(t) + \sum_{j \neq i} \mathbf{A}_{ij} (f_j^\mu(t) - f_i^\mu(t)) \\ &= (1 - \sum_{j \neq i} \mathbf{A}_{ij}) f_i^\mu(t) + \sum_{j \neq i} \mathbf{A}_{ij} f_j^\mu(t) \\ &\equiv \sum_j \mathbf{A}_{ij} f_j^\mu(t), \end{aligned} \quad \text{[S.59]}$$

where $\mathbf{A}_{ii} \equiv 1 - \sum_{j \neq i} \mathbf{A}_{ij}$ (or equivalently, $\sum_j \mathbf{A}_{ij} = 1$). We note that the diagonal elements $\mathbf{A}_{ii}$ in the time-discrete dynamics are determined from the normalization, and the transmission rate within a deme, β_{ii}, does not enter the neutral dynamics.

In ref. (10), β_{ij} is modeled in terms of human mobility as

$$\beta_{ij} = \beta \frac{f_{i \to j} p_j + f_{j \to i} p_i}{N_i}, \quad \text{[S.60]}$$

where β is the transmission rate when a susceptible individual has contact with an infected, p_i is the contact probability in deme i, and $f_{i \to j}$ is the mobility flux from i to j. The first term in the numerator corresponds to the case that a susceptible person in deme i visits deme j and gets infected by a residence of deme j, while the second term corresponds to the case that an infected person in deme j visits

deme i and infects a resident of deme i. Note that, in ref. (10), p_i is assumed to be the same for all demes. Using Eq. (S.60), $\mathbf{A}_{ij}$ in Eq. (S.58) can be expressed in terms of the mobility flux as

$$\begin{aligned}\mathbf{A}_{ij} &= \beta s_i \frac{f_{i\to j}p_j + f_{j\to i}p_i}{N_i}\frac{i_j}{i_i} \\ &= \beta \frac{S_i I_j}{I_i}\frac{f_{i\to j}p_j + f_{j\to i}p_i}{N_i N_j}.\end{aligned} \quad \text{[S.61]}$$

The expression in Eq. (S.61) implies:

1. The combination $\mathbf{A}_{ij}\frac{I_i}{S_i I_j}$ is symmetric in i and j.
2. The asymmetry in the couplings between i and j is given by $\frac{\mathbf{A}_{ij}}{\mathbf{A}_{ji}} = \frac{S_i I_j^2}{S_j I_i^2} \approx \frac{N_j}{N_i}$, where the densities s_i and i_i are assumed to be approximately the same for all demes. This expression of the asymmetry means that a deme with a larger population size is expected to have a greater impact on the other.
3. $\mathbf{A}_{ij}$ in Eq. (S.61) satisfies the detailed balance condition. Specifically, the reproductive value(the left null vector of $\mathbf{A}_{ij}$) is given by $\Pi_i \propto \frac{I_i^2}{S_i} \approx N_i$.

S.6.2 Non-neutral variant.
Consider the case of two non-neutral lineages, specifically the Alpha and Delta variants ($\mu = \alpha, \delta$). We express β_{ij}^{μ} and γ^{μ} for these variants as follows: $\beta_{ij}^{\alpha} = \beta_{ij}(1-\epsilon_\beta), \gamma^{\alpha} = \gamma(1+\epsilon_\gamma), \beta_{ij}^{\delta} = \beta_{ij}, \gamma^{\delta} = \gamma$ and denote the Delta's frequency as $f_i^{\Delta}(t) = f_i(t)$.

Using these expressions, Eq. (S.56) for $\mu = \delta$ becomes:

$$\dot{f}_i = \sum_{j\neq i}\mathbf{A}_{ij}(f_j - f_i) + (s_i\epsilon_\beta\beta_{ii} + \epsilon_\gamma\gamma)f_i(1-f_i) + \epsilon_\beta\sum_{j\neq i}\mathbf{A}_{ij}f_i(1-f_j), \quad \text{[S.62]}$$

where $\mathbf{A}_{ij} \equiv s_i\frac{i_j}{i_i}\beta_{ij}$ denotes the importation-rate matrix for the Delta variant. When the Delta variant is rare, $f_i \ll 1$, we can drop the terms quadratic in frequencies:

$$\begin{aligned}\dot{f}_i &\approx \sum_{j\neq i}\mathbf{A}_{ij}(f_j - f_i) + (s_i\epsilon_\beta\beta_{ii} + \epsilon_\gamma\gamma + \epsilon_\beta\sum_{j\neq i}\mathbf{A}_{ij})f_i \\ &= \sum_{j\neq i}\mathbf{A}_{ij}(f_j - f_i) + \sigma_i f_i,\end{aligned} \quad \text{[S.63]}$$

where $\sigma_i \equiv s_i\epsilon_\beta\beta_{ii} + \epsilon_\gamma\gamma + \epsilon_\beta\sum_{j\neq i}\mathbf{A}_{ij}$. In the main text, the subscript of σ_i is dropped, assuming that $s_i\epsilon_\beta\beta_{ii}$ and $\sum_{j\neq i}\mathbf{A}_{ij} (= 1 - \mathbf{A}_{ii})$ are not significantly different across populations.

S.6.3 Other epidemiological scenarios. While we verified the linear dynamics $f_i(t+1) = \sum_j \mathbf{A}_{ij}f_j(t)$ in Eq. S.59 assuming the standard transmission function $\beta SI/N$, it is worth noting that this result holds independently of specific transmission functions employed (see ref. (18) for a variety of transmission functions). For instance, if the transmission function takes the form $\beta_{ij}S_i^p I_j^q$ with some exponents $p, q > 0$, Eq. S.52 would be replaced by:

$$\dot{I}_i^{\mu} = (S_i)^p \sum_j \beta_{ij}(I_j)^q f_j^{\mu} - \gamma^{\mu} I_i^{\mu} f_i^{\mu}. \quad \text{[S.64]}$$

Note that the transmission term is linear in f_j^{μ}, which guarantees that the sum over lineages yields the total transmission rate. More generally, Eq. S.52 can be generalized to:

$$\dot{I}_i^{\mu} = G_{ij}f_i^{\mu}, \quad \text{[S.65]}$$

where G_{ij} is any lineage-independent function that may depend on the total numbers of susceptible and infected individuals, as well as any other lineage-independent quantities such as population densities and geographic distances. It can then be shown that the neutral dynamics of a lineage frequency is given by Eq. S.57 with:

$$\mathbf{A}_{ij} = \frac{G_{ij}}{I_i}. \quad \text{[S.66]}$$

Furthermore, the linear dynamics $f_i(t+1) = \sum_j \mathbf{A}_{ij}f_j(t)$ can also be justified in another important class of epidemiological models, the SEIR model, which has been widely applied to SARS-CoV-2 (refs. (15, 19)). A multi-patch extension of the SEIR model is described by the following equations:

$$\dot{S}_i = -\sum_{\mu}\sum_j S_i\beta_{ij}\frac{I_j^{\mu}}{N_j}, \quad \text{[S.67]}$$

$$\dot{E}_i^{\mu} = \sum_j S_i\beta_{ij}\frac{I_j^{\mu}}{N_j} - \gamma_E E_i^{\mu}, \quad \text{[S.68]}$$

$$\dot{I}_i^{\mu} = \gamma_E E_i^{\mu} - \gamma_I I_i^{\mu}, \quad \text{[S.69]}$$

$$\dot{R}_i = \sum_{\mu}\gamma_I I_i^{\mu}. \quad \text{[S.70]}$$

Here, E_i^μ represents the number of asymptomatic individuals in deme i who have been infected by lineage μ. γ_E is the rate at which an exposed individual becomes infectious (the inverse of the average latent time), and γ_I is the rate at which an infectious individual recovers. The total population size, $N_i = S_i + I_i + E_i + R_i$, is constant in each deme, where $I_i = \sum_\mu I_i^\mu$ and $E_i = \sum_\mu E_i^\mu$, respectively, represent the total infected and exposed populations in deme i across all lineages.

We can show that the lineage frequencies, $f_i^\mu \equiv \frac{I_i^\mu}{I_i}$ and $g_i^\mu \equiv \frac{E_i^\mu}{E_i}$, among exposed and infected individuals, obey the following equations:

$$\dot{f}_i^\mu = \gamma_E \frac{E_i}{I_i}(g_i^\mu - f_i^\mu), \qquad \text{[S.71]}$$

$$\dot{g}_i^\mu = \sum_{j \neq i} A_{ij}^{\text{SEIR}}(f_j^\mu - g_i^\mu) + \beta_{ii}\frac{s_i}{e_i} i_i (f_i^\mu - g_i^\mu), \qquad \text{[S.72]}$$

with

$$\mathbf{A}_{ij}^{\text{SEIR}} = \beta_{ij}\frac{s_i}{e_i} i_j = \frac{i_i}{e_i}\mathbf{A}_{ij}^{\text{SIR}}, \qquad \text{[S.73]}$$

where $s_i = \frac{S_i}{N_i}$, $e_i = \frac{E_i}{N_i}$, $i_j = \frac{I_j}{N_j}$ are the fractions of these epidemiological classes, and $\mathbf{A}_{ij}^{\text{SIR}}$ is the matrix in Eq. S.58. The matrix $\mathbf{A}_{ij}^{\text{SEIR}}$ differs from $\mathbf{A}_{ij}^{\text{SIR}}$ by the factor $\frac{i_i}{e_i}$. This factor is largely determined by the ratio $\frac{\gamma_E}{\gamma_I}$ (see Eq. S.69) and takes similar values across demes.

By subtracting Eqs. S.71 and S.72, we obtain

$$\begin{aligned}\frac{d}{dt}(f_i^\mu - g_i^\mu) &= -\left(\gamma_E \frac{E_i}{I_i} + \beta_{ii}\frac{s_i}{e_i} i_i\right)(f_i^\mu - g_i^\mu) - \sum_{j \neq i} \mathbf{A}_{ij}^{\text{SEIR}}(f_j^\mu - g_i^\mu) \\ &\approx -\left(\gamma_E \frac{E_i}{I_i} + \beta_{ii}\frac{s_i}{e_i} i_i\right)(f_i^\mu - g_i^\mu),\end{aligned} \qquad \text{[S.74]}$$

where we dropped the off-diagonal couplings, which are usually smaller than the within-deme transmission, $\beta_{ii}\frac{s_i}{e_i}i_i$. The coefficients $\gamma_E \frac{E_i}{I_i}$ and $\beta_{ii}\frac{s_i}{e_i}i_i$ in Eq. S.74 can be roughly approximated as $\gamma_E \frac{E_i}{I_i} \approx \gamma_E \frac{\gamma_I}{\gamma_E} = \gamma_I$ and $\beta_{ii}\frac{s_i}{e_i}i_i \approx \beta_{ii}\frac{i_i}{e_i} \approx \beta_{ii}\frac{\gamma_E}{\gamma_I}$. Evaluating these with realistic parameter values (for instance, $\beta_{ii} = 0.4\,\text{day}^{-1}$, $\gamma_E = (3.0\,\text{day})^{-1}$, $\gamma_I = (5.5\,\text{day})^{-1}$ (ref. (19)), we observe that the difference $f_i^\mu - g_i^\mu$ relaxes to 0 within 1 week. Thus, we may practically identify f_i^μ and g_i^μ. Consequently, from Eq. S.72, with this identification, we find that in the SEIR model, the dynamics of the lineage frequency f_i^μ is described by

$$\dot{f}_i^\mu \approx \sum_{j \neq i} \mathbf{A}_{ij}^{\text{SEIR}}(f_j^\mu - f_i^\mu), \qquad \text{[S.75]}$$

where, as argued above, $\mathbf{A}_{ij}^{\text{SEIR}} \equiv \beta_{ij}\frac{s_i}{e_i}i_j \approx \frac{\gamma_E}{\gamma_I}\mathbf{A}_{ij}^{\text{SIR}}$.

S.7 The jump-size distribution calculated from the importation-rate matrix $\mathbf{A}$

In Fig. 3C, we computed the probability distribution of per-individual jump distances, assuming that a jump from deme j to deme i occurs with probability proportional to

$$N_i A_{ij}/Z,$$

where the normalization factor Z is given by $Z = \sum_{i \neq j} N_i A_{ij}$. The rationale for this population-size-dependent rescaling is as follows: the probability that a randomly selected infection is in deme i is proportional to the number of infected individuals I_i, which is approximately proportional to the population size N_i. Since A_{ij} represents the probability that an infection in deme i was imported from deme j, the overall probability that a randomly selected infection event occurred from j to i is proportional to $N_i A_{ij}$.

This probability can also be expressed in terms of mobility under the SIR model. As described in SI Sec. S.6, the derivation of Eq. S.60 considers two possibilities for an infection event: (i) a susceptible individual in deme i visits deme j and is infected by a resident of deme j; and (ii) an infected individual from deme j visits deme i and infects a resident there. Assuming that the second contribution dominates, we obtain

$$A_{ij} \approx \beta p \frac{f_{j \to i}}{N_i}$$

instead of Eq. S.61. In this case, the probability that a randomly selected infection event occurred from j to i is proportional to $N_i A_{ij} \propto f_{j \to i}$.

We note that there are different sampling schemes for target-source pairs (i, j). For example, one may sample a random deme i uniformly and consider an infected individual within that deme, and then ask from which deme j the infection originated. In this case, the pair (i, j) is sampled with probability $\mathbf{A}_{ij}$. While different sampling schemes introduce different population-size-dependent factors, the exponent of the jump-size distribution is relatively robust to this choice (Fig. S19 for the results for the Delta wave in England) because population sizes do not vary significantly across demes, owing to the deme construction described in Sec. S.13.1.

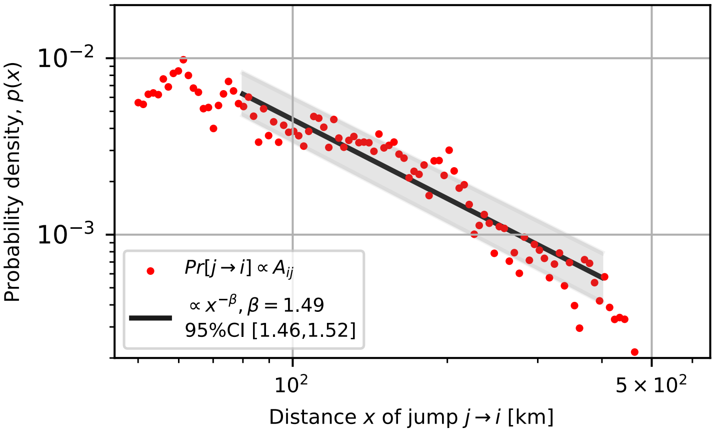

Fig. S19. The jump-size distributions for a different sampling scheme for the Delta wave in England. Here, target-source pairs (i, j) are sampled with probability $\mathbf{A}_{ij}$.

S.8 Distance between demes used for the hierarchical clustering of $\mathbf{A}_{ij}$ and multidimensional scaling analysis

For the deme-resolved coupling matrices $\mathbf{A}$ shown in Figs. 3 and 6, we ordered demes according to how similar their rows are. To this end, we performed hierarchical clustering using Ward's method with the following metric d_{ij} between populations;

$$d_{ij} = D^{JS}(\mathbf{A}_i^{\widehat{ij}}, \mathbf{A}_j^{\widehat{ij}}), \qquad \text{[S.76]}$$

where $D^{JS}(\boldsymbol{p}, \boldsymbol{q})$ is the square root of the Jensen-Shannon divergence between two probability distributions $\boldsymbol{p}$ and $\boldsymbol{q}$. $\mathbf{A}_i^{\widehat{ij}} \in \mathbb{R}^{N_D - 2}$ (resp. $\mathbf{A}_j^{\widehat{ij}} \in \mathbb{R}^{N_D - 2}$) is the vector of the probability distribution obtained by removing the i-th and j-th elements from the i-th (resp. j-th) row of the importation-rate matrix. Explicitly, their k-th elements are given by

$$(\mathbf{A}_i^{\widehat{ij}})_k \equiv \frac{\mathbf{A}_{ik}}{\sum_{j' \neq i,j} \mathbf{A}_{ij'}}, \quad (\mathbf{A}_j^{\widehat{ij}})_k \equiv \frac{\mathbf{A}_{jk}}{\sum_{j' \neq i,j} \mathbf{A}_{jj'}}. \qquad \text{[S.77]}$$

In the backward-in-time interpretation, the metric d_{ij} compares the probability distribution of the spatial locations of a lineage starting at population i and the one starting at population j, conditional on that they go to populations outside of i and j.

Figures S20A and B show the results of hierarchical clustering using Ward's method, and the MDS analysis with the above metric for the matrix powers $\mathbf{A}^n$ $(n = 1, 5, 10, 20)$, where $\mathbf{A}$ is the importation-rate matrix for the Delta wave in England (Fig. 3). Fig. S20C compares the Jensen-Shannon divergence to the physical distances between demes. We can see that the clustering of the 50 demes is relatively robust to the timescale n of interest. However, as n becomes large (compared to the timescale of relaxation, approximately $n = 10$ weeks), all rows of $(\mathbf{A}^n)_{ij}$ approach the reproductive value$\boldsymbol{\pi}$ (i.e., the left eigenvector of $\mathbf{A}$ corresponding to the eigenvalue 1), and the Jensen-Shannon divergence becomes less informative of physical distances. For example, for $(\mathbf{A}^{20})_{ij}$, demes in LDN, SE, and SW become highly clustered in the MDS plot (the bottom panel of Fig. S20B), indicating that the Jensen-Shannon divergence cannot effectively distinguish between physically close demes (the bottom panel of Fig. S20C).

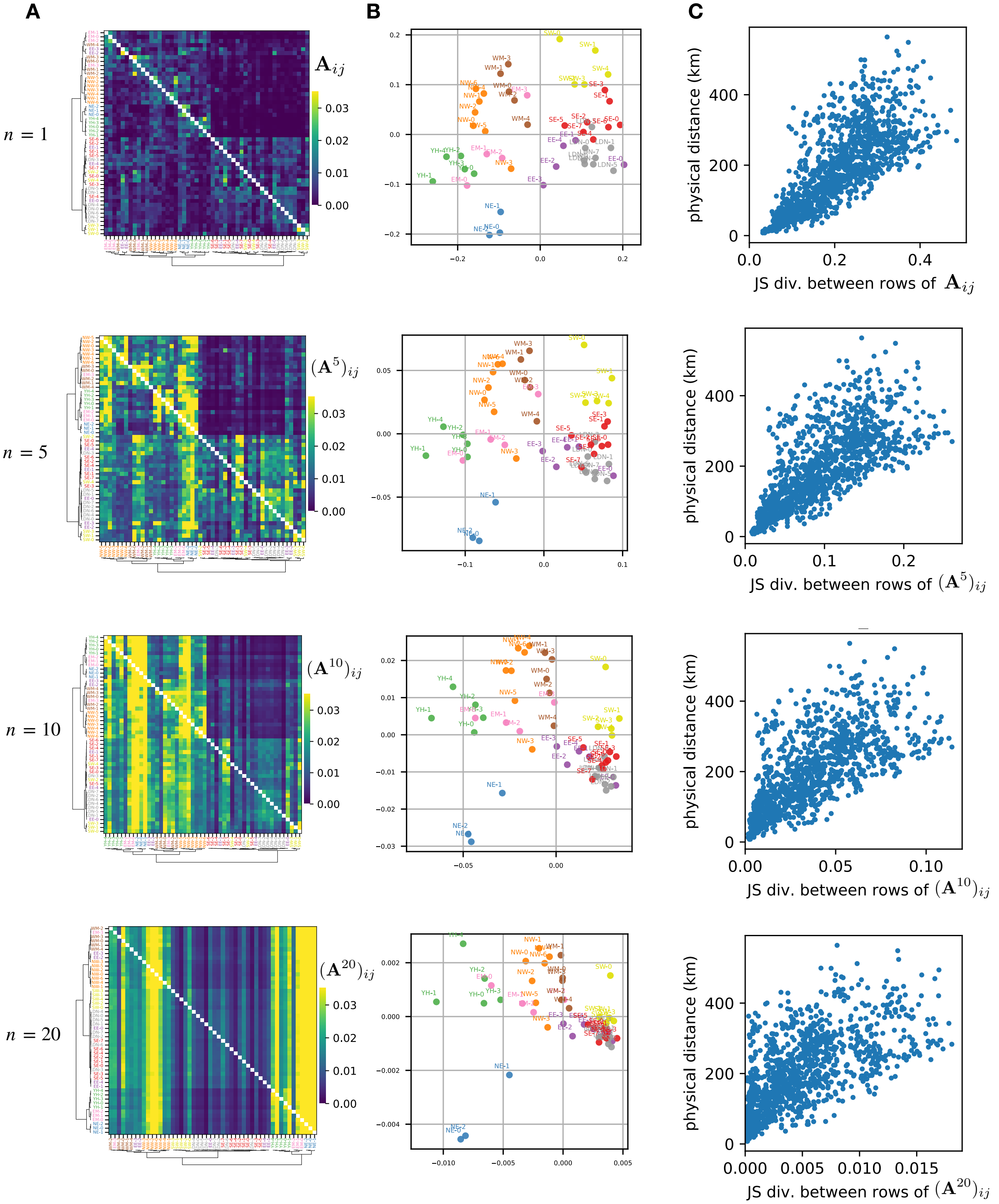

Fig. S20. Clustering and distances based on the Jensen-Shannon divergence for the $(\mathbf{A}^n)$ for $n = 1,\ 5,\ 10,\ 20$, where $\mathbf{A}$ is the 50×50 matrix for the Delta wave (weeks 78-91) in England. (**A**) Heatmaps of $\mathbf{A}^n (n = 1, 5, 10, 20)$, where hierarchical clustering is performed using the Ward's method with the Jensen-Shannon divergence between the rows of $\mathbf{A}^n$. (**B**) Multidimensional scaling plots. (**C**) Comparison between the Jensen-Shannon divergence and physical distance, with each dot representing these two distances for a specific pair of demes i and j.

S.9 Constrained inference without long-range transmission

In the main text (Fig. 7), we showed that zeroing long-range importation rates of the inferred $\mathbf{A}_{ij}$ significantly increases the relaxation time of the second slowest mode, suggesting that long-range connections—despite their small individual values—play an important role in shaping the system's dynamics.

However, one might question whether these small-valued long-range importation rates are truly necessary to explain the observed allele frequency data. To address this, we re-inferred the importation rates $\mathbf{A}_{ij}$ under a constraint that removes non-neighboring connections—that is, by disallowing transmissions between non-neighboring regions. We found that the HMM likelihood of the constrained model is significantly lower than that of the unconstrained model; the likelihood ratio statistic $2\Delta \log \mathcal{L}$ is 54.6 for the Alpha variant and 78.1 for Delta. From the chi-squared distribution with 42 degrees of freedom (corresponding to the number of non-neighboring connections among the nine regions), the p-values are $p = 0.092$ for the Alpha variant and $p = 6.0 \times 10^{-4}$ for the Delta variant. While the Delta results indicate strong evidence for non-neighboring connections, the evidence for Alpha is marginal. One possible reason is the lower sequence reporting rate during the Alpha wave than during the Delta wave, which could make the infernece less accurate. Another possibility is that the relaxation time for Alpha ($\sim$26 weeks; Fig. 7B) exceeds the 14-week plateau window used for inference; becaue small importation rates manifest only after long periods, their effects do not accumulate sufficiently within this window to be estimated reliably.

To assess how the constraint affects the inference, we also compared the short-range importation rates $\mathbf{A}_{ij}$ inferred under the constraint with those inferred without it (Fig. S21A). We found that the constrained model yields short-range (i.e., between neighboring regions) importation rates that are relatively similar to those of the unconstrained model, indicating that the inference of local transmission patterns is robust to the presence or absence of long-range links. However, as expected, the short-range importation rates inferred under the constraint are slightly larger than those in the unconstrained model, as the constrained model compensates for the absence of long-range connections by adjusting short-range rates.

As in the zeroing-after-inference analysis presented in the main text (Fig. 7), the constrained inference (i.e., zeroing during inference) yields substantially longer relaxation times $\tau_1 = -1/\ln|\lambda_1|$ relative to the unconstrained case (Fig. S21B).

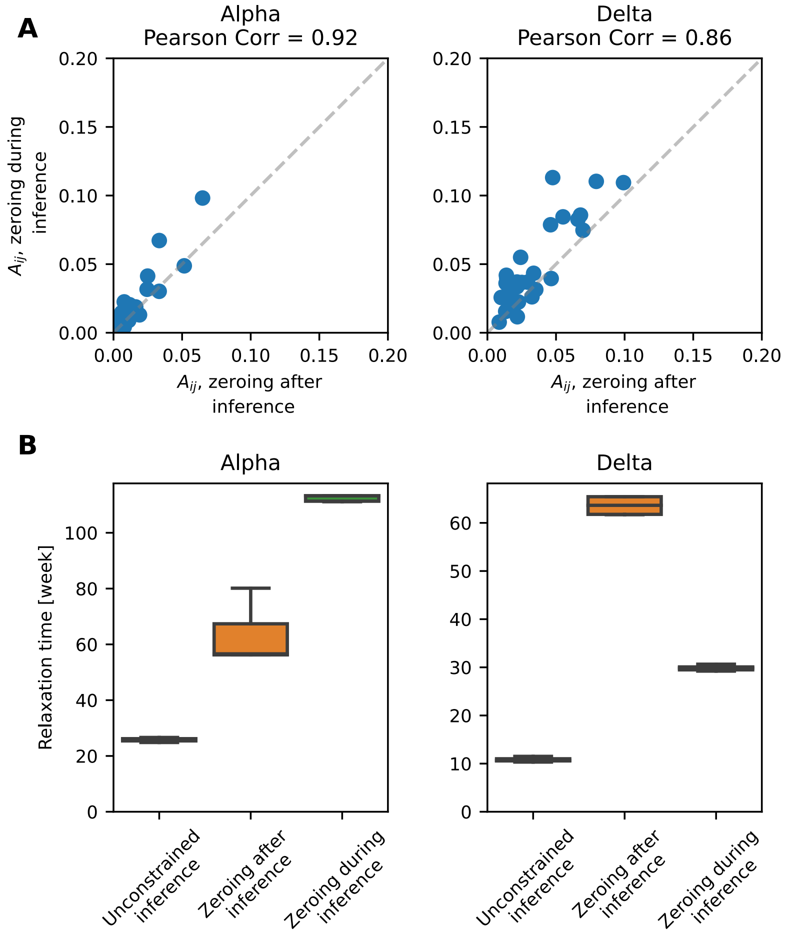

Fig. S21. Comparison between two approaches for zeroing long-range connections: zeroing after inference and zeroing during inference (constrained inference). (**A**) Element-wise comparison between the inferred importation rates $\mathbf{A}_{ij}$ across neighboring regions, with and without constraints on non-neighboring connections. Each panel corresponds to the Alpha and Delta variants; Pearson correlation coefficients are indicated. (**B**) Relaxation times of the second slowest mode for the unconstrained importation-rate matrix, the zeroing-after-inference matrix, and the constrained-inference matrix.

S.10 Prediction of the spreading dynamics of the Delta variant shown in Fig. 8

Non-neutral transmission dynamics: We modeled the frequency dynamics of the Delta variant in England using the ordinary differential equation in the form of Eq. S.62:

$$\dot{f}_i = \sum_{j\neq i} \mathbf{A}_{ij}(f_j - f_i) + \tilde{\sigma} f_i(1-f_i) + \epsilon_\beta \sum_{j\neq i} \mathbf{A}_{ij} f_i(1-f_j), \qquad \text{[S.78]}$$

where $\mathbf{A}_{ij}$ represents the 50×50 importation-rate matrix presented in Fig. 3, and $\tilde{\sigma}$ and ϵ_β are fit parameters.

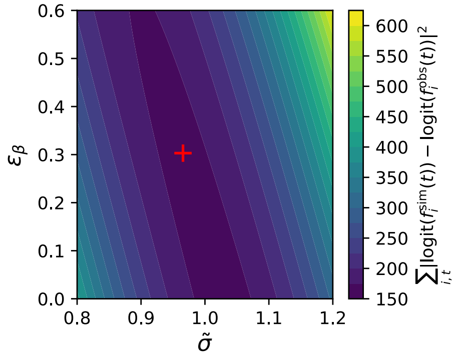

Fig. S22. Heatmap showing the discrepancy between theoretical predictions and observed data, defined in Eq. S.79, as a function of $(\tilde{\sigma}, \epsilon_\beta)$. The parameter values, indicated by the red cross, are used for the results presented in the main text.

Determination of the parameter values: To fix $\tilde{\sigma}$ and ϵ, we simulated the above ODE and identified the optimal parameter values that minimize the discrepancies between our model's predictions and the observed frequencies in a logit scale:

$$\min_{\tilde{\sigma},\epsilon_\beta} \sum_{i,t\in D_{i,t}} \left(\log_{10} \frac{f_i^{\text{sim}}(t)}{1-f_i^{\text{sim}}(t)} - \log_{10} \frac{f_i^{\text{obs}}(t)}{1-f_i^{\text{obs}}(t)} \right)^2, \qquad \text{[S.79]}$$

where $f_i^{\text{sim}}(t)$ and $f_i^{\text{obs}}(t)$ denote the simulated and observed frequencies, respectively, of the Delta variant. The data points $D_{(i,t)} \equiv \{(i,t)|\ 0.05 < f_i^{\text{obs}}(t) < 0.95$ and (the number of sequences reported from location i in week t) $> 10\}$ are used for the fitting.

We examined the discrepancy between theoretical predictions and observed data, Eq. S.79, across a range of parameter values (Fig. S22). The discrepancy has a weak dependence on ϵ_β, indicating that it cannot be reliably estimated solely from our fitting process. Therefore, we referred to the relative infectivity reported in ref. (20), which found that the Delta variant is 43-68% more transmissible than the Alpha variant, corresponding to ϵ_β values between 0.30 and 0.40 (see Eq. S.62 and the definition of ϵ_β). Based on these, we constrained our optimization search to this realistic range, resulting in the optimal values, $(\tilde{\sigma}, \epsilon_\beta) = (0.97, 0.30)$. These parameter values are used in the simulations presented in the main text.

Determination of the mid timepoint $t_{1/2}$: To compare the observed and simulated frequency trajectories, we calculated the mid timepoint $t_{1/2}$ for each trajectory $f_i(t)$ by fitting it to a logistic curve, $(1+e^{-l(t-t_{1/2})})^{-1}$, where l and $t_{1/2}$ are fit parameters (see Fig. S23 for the actual trajectories and the fitting results).

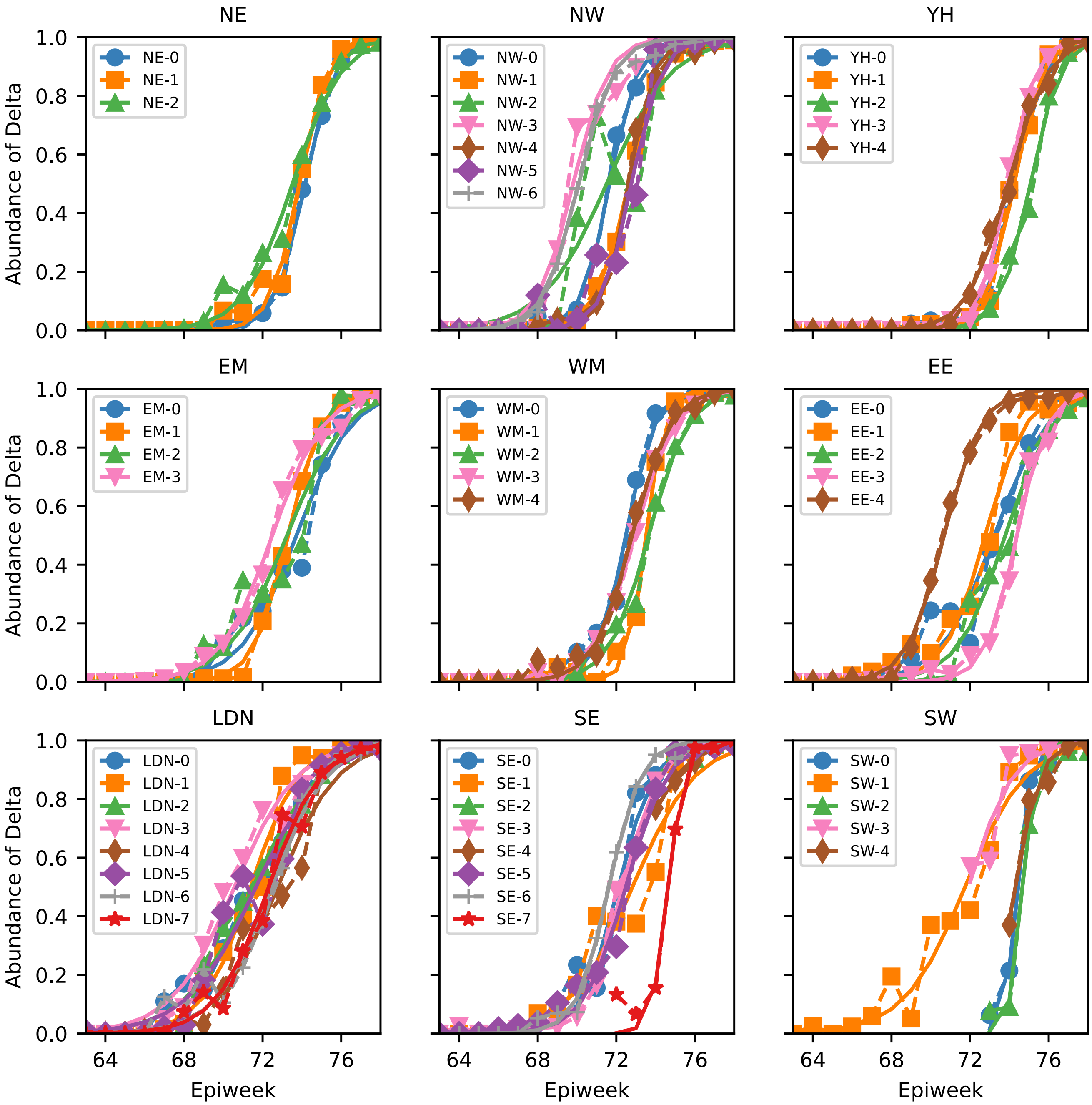

Fig. S23. Procedure of determining $t_{1/2}$ for the actual frequencies of the Delta variant in England. In each panel, the dashed lines represent the observed frequencies $f_i^{\mathrm{obs}}(t)$ in demes within a region, while the solid lines represent fitted logistic curves, $f(t) = \frac{1}{1+\exp[-k(t-t_{1/2})]}$, where k and $t_{1/2}$ are fit parameters. For each deme, the time window with more than 10 sequences is used for the fitting. The inflection points $t_{1/2}$ of these fitted logistic curves are used in Fig. 8 of the main text.

S.11 Multiplicity-Adjusted Bayesian Detection of Directional Asymmetry

S.11.1 Asymmetry in the 3x3 importation-rate matrix $\mathbf{A}_{ij}$ in Fig. 4.

Let i, j index the three regions $\{\mathrm{LDN}, \mathrm{EE}, \mathrm{SE}\}$ in England, and let $\mathbf{A}_t$ denote the importation-rate matrix at time $t \in \{1, \dots, K\}$ during the wave of a variant. Directed asymmetry from j to i is summarized by the time-averaged log ratio

$$\mu_{ij} \;=\; \frac{1}{K} \sum_{t=1}^{K} \ln\left(\frac{A_{t,ij}}{A_{t,ji}}\right). \qquad \text{[S.80]}$$

The posterior distribution $p(\mu_{ij} \mid \mathrm{data})$ is induced by $\{p(\mathbf{A}_t \mid \mathrm{data})\}_{t=1}^{K}$, where each $p(\mathbf{A}_t \mid \mathrm{data})$ is obtained by fitting the HMM method to a 7-week window $[t-3, t+3]$ of the time series.

The objective is to identify the set S of ordered pairs with positive asymmetry, $\mu_{ij} > 0$, with high posterior certainty. Define the latent indicator of the true direction $r_{ij} = \mathbf{1}\{\mu_{ij} > 0\} \in \{0, 1\}$. For any selected set S, the posterior expected number $\overline{FD}$ of false discoveries (pairs in S with $\mu_{ij} \le 0$) equals

$$\overline{\mathrm{FD}} \equiv \mathbb{E}_{p(\mu|\mathrm{data})}\left[\sum_{(i,j)\in S} \Big(1 - r_{ij}\Big)\right] \;=\; \sum_{(i,j)\in S} \Pr\Big(\mu_{ij} \le 0 \mid \mathrm{data}\Big). \qquad \text{[S.81]}$$

We determine S as the largest set of pairs satisfying (21)

$$\overline{\mathrm{FD}} \le \alpha, \qquad \text{[S.82]}$$

for a prespecified level $\alpha \in (0, 1)$.

Note that the condition Eq. (S.82) also bounds the family-wise error rate (FWER). Specifically, by Boole's inequality (the union bound), the posterior probability that S contains at least one false positive satisfies

$$\mathrm{FWER} \equiv \Pr\Big(\exists (i,j) \in S : \mu_{ij} \le 0 \;\Big|\; \mathrm{data}\Big) \le \sum_{(i,j)\in S} \Pr\Big(\mu_{ij} \le 0 \mid \mathrm{data}\Big) = \overline{\mathrm{FD}} \le \alpha. \qquad \text{[S.83]}$$

This decision rule is conceptually analogous to the frequentist Bonferroni procedure: both control the probability of at least one false positive in the selected set.

Table 1 shows the median (with 95% credible interval) of asymmetry μ_{ij} and the posterior false-discovery probability $\Pr(\mu_{ij} \le 0)$. To facilitate determining the largest set S, rows of the table are sorted in ascending order of $\Pr(\mu_{ij} \le 0)$. With $\alpha = 0.05$, the set $S = \{(\mathrm{EE}, \mathrm{LDN}),\ (\mathrm{SE}, \mathrm{LDN})\}$ is selected as the set of positively asymmetric pairs with high probability, for both the Alpha and Delta variants.

Alpha (i,j)	Median of μ_{ij}	95% CrI	$\Pr(\mu_{ij} \le 0)$	$\overline{\mathrm{FD}}$
EE,LDN	3.60	[2.11, 6.9]	0.0000	0.0000
SE,LDN	2.52	[1.65, 3.95]	0.0002	0.0002
SE,EE	1.27	[0.61, 2.72]	0.2627	0.2629

Delta (i,j)	Median of μ_{ij}	95% CrI	$\Pr(\mu_{ij} \le 0)$	$\overline{\mathrm{FD}}$
EE,LDN	1.64	[1.24, 2.19]	0.0009	0.0009
SE,LDN	1.56	[1.21, 2.09]	0.0010	0.0019
EE,SE	1.36	[0.82, 2.29]	0.1125	0.1144

Omicron (i,j)	Median of μ_{ij}	95% CrI	$\Pr(\mu_{ij} \le 0)$	$\overline{\mathrm{FD}}$
SE,LDN	1.70	[0.41, 6.31]	0.1885	0.1885
SE,EE	1.34	[0.25, 6.74]	0.3347	0.5232
EE,LDN	1.16	[0.27, 4.8]	0.3984	0.9216

Table 1. Pairwise directional asymmetry μ_{ij} within each variant wave (Alpha, Delta, Omicron). Within each wave, rows are ordered by increasing $\Pr(\mu_{ij} \le 0)$. Entries show the posterior median and 95% credible interval for μ_{ij}. The last column reports the cumulative expected number of false discoveries up to that row, $\overline{\mathrm{FD}} = \sum_{(i,j)\in S} \Pr(\mu_{ij} \le 0)$, where S is the set of pairs up to that row. Only pairs with $\Pr(\mu_{ij} \le 0) < 0.5$ are shown. With $\alpha = 0.05$, the largest S contains the pairs above the dashed line ($S = \emptyset$ for Omicron).

S.11.2 Temporal shift in $\mathbf{A}_{ij}$ in Fig. 4.

For each wave (Alpha or Delta) of K time points, we split the time points into a first half ($t = 1, \dots, \frac{K}{2}$) and a second half ($t = \frac{K}{2} + 1, \dots, K$) and quantify asymmetry as before:

$$\mu_{ij}^{\mathrm{first}} = \frac{2}{K} \sum_{t=1}^{K/2} \ln\left(\frac{A_{t,ij}}{A_{t,ji}}\right), \qquad \text{[S.84]}$$

$$\mu_{ij}^{\mathrm{second}} = \frac{2}{K} \sum_{t=K/2+1}^{K} \ln\left(\frac{A_{t,ij}}{A_{t,ji}}\right). \qquad \text{[S.85]}$$

We assess a possible shift in asymmetry by their difference,

$$\Delta\mu_{ij} \;\equiv\; \mu_{ij}^{\text{second}} - \mu_{ij}^{\text{first}}. \qquad \text{[S.86]}$$

To identify pairs with a positive temporal shift ($\Delta\mu_{ij} > 0$), we apply the Bayesian selection criterion to $\Delta\mu_{ij}$ and determine the largest set S satisfying

$$\overline{\text{FD}} = \sum_{(i,j)\in S} \Pr\Big(\Delta\mu_{ij} \le 0 \mid \text{data}\Big) \;\le\; \alpha. \qquad \text{[S.87]}$$

Table 2 reports the posterior median (95% CrI) of the temporal shift $\Delta\mu_{ij}$ and the posterior false-discovery probability $\Pr(\Delta\mu_{ij} \le 0)$. Rows are sorted in ascending order of $\Pr(\Delta\mu_{ij} \le 0)$. With $\alpha = 0.05$, $\Delta\mu_{\text{SE,LDN}}$ (Delta wave) is selected as the pair for which the asymmetry shifted over time during the wave with high posterior probability.

Alpha (i,j)	Median of $\Delta\mu_{ij}$	95% CrI	$\Pr(\Delta\mu_{ij} \le 0)$	$\overline{\text{FD}}$
SE,EE	1.08	[-0.4, 2.6]	0.0728	0.0728
LDN,EE	0.26	[-0.86, 1.5]	0.3146	0.3874
SE, LDN	0.17	[-0.65, 1.1]	0.3387	0.7261

Delta (i,j)	Median of $\Delta\mu_{ij}$	95% CrI	$\Pr(\Delta\mu_{ij} \le 0)$	$\overline{\text{FD}}$
SE,LDN	0.55	[0.08, 1.18]	0.0111	0.0111
EE,LDN	0.38	[-0.13, 1.0]	0.0719	0.0830
EE,SE	0.04	[-0.97, 1.07]	0.4664	0.5494

Table 2. Temporal shift in directional asymmetry ($\Delta\mu_{ij}$) for Alpha and Delta. Within each wave, rows are ordered by increasing $\Pr(\Delta\mu_{ij} \le 0)$. Entries show the posterior median and 95% credible interval for $\Delta\mu_{ij}$. The last column gives the cumulative expected number of false discoveries up to that row, $\overline{\text{FD}} = \sum_{(i,j)\in S} \Pr(\Delta\mu_{ij} \le 0)$, where S is the set of pairs up to that row. Only pairs with $\Pr(\Delta\mu_{ij} \le 0) < 0.5$ are shown. With $\alpha = 0.05$, the largest S contains the pair above the dashed line.

S.11.3 Heterogeneity in reproductive values in Fig. 7.

We assess spatial heterogeneity in per-capita reproductive values shown in Fig. 7. Here, $i, j \in \{\text{LDN}, \text{EE}, \text{SE}, \text{SW}, \text{WM}, \text{EM}, \text{NW}, \text{NE\&YH}\}$. For each wave (Alpha or Delta), we compare demes i and j via the log ratio

$$\Phi_{ij} \;\equiv\; \log\left(\frac{\pi_i/I_i}{\pi_j/I_j}\right), \qquad \text{[S.88]}$$

where π_i is the fixation probability (satisfying $\sum_k \pi_k \mathbf{A}_{kj} = \pi_j$ and $\sum_k \pi_k = 1$) and I_i is the number of infected individuals in deme i.

We apply the Bayesian selection criterion to Φ_{ij} and determine the largest set S of pairs satisfying

$$\overline{\text{FD}} = \sum_{(i,j)\in S} \Pr\Big(\Phi_{ij} \le 0 \mid \text{data}\Big) \;\le\; \alpha. \qquad \text{[S.89]}$$

Table 3 reports the posterior median (95% CrI) of Φ_{ij} and the posterior false-discovery probability for the ten pairs with the smallest values of $\Pr(\Phi_{ij} \le 0)$. Rows are sorted in ascending order of $\Pr(\Phi_{ij} \le 0)$. With $\alpha = 0.05$, five pairs for the Alpha variant and three pairs for the Delta variant are selected as exhibiting positive differences with high posterior probability.

Alpha (i, j)	Median of Φ_{ij}	95% CrI	$\Pr(\Phi_{ij} \leq 0)$	$\overline{\text{FD}}$
SW,EE	2.86	[1.44,4.54]	0.0000	0.0000
EM,EE	2.36	[1.19,4.66]	0.0010	0.0010
SW,NEYH	1.88	[1.06,3.27]	0.0124	0.0134
SW,LDN	1.53	[1.01,2.53]	0.0136	0.0270
SE,EE	2.16	[1.03,4.04]	0.0208	0.0478
SW,WM	1.60	[0.81,2.82]	0.0576	0.1054
LDN,EE	1.83	[0.88,3.71]	0.0628	0.1682
EM,NEYH	1.64	[0.82,2.61]	0.1058	0.2740
WM,EE	1.79	[0.83,3.6]	0.1078	0.3818
NW,EE	2.35	[0.68,5.42]	0.1290	0.5108

Delta (i, j)	Median of Φ_{ij}	95% CrI	$\Pr(\Phi_{ij} \leq 0)$	$\overline{\text{FD}}$
LDN,EE	1.85	[1.16,4.32]	0.0000	0.0000
WM,EE	1.89	[1.13,3.71]	0.0104	0.0104
EM,EE	1.84	[1.03,3.78]	0.0176	0.0280
NEYH,EE	1.54	[0.96,2.96]	0.0370	0.0650
SE,EE	1.44	[0.94,4.75]	0.0544	0.1194
LDN,SW	1.53	[0.83,2.65]	0.1204	0.2398
WM,SW	1.50	[0.75,2.73]	0.1460	0.3858
LDN,NEYH	1.23	[0.78,1.96]	0.1692	0.5550
WM,SE	1.24	[0.67,1.96]	0.1876	0.7426
LDN,SE	1.26	[0.85,1.95]	0.1906	0.9332

Table 3. Log ratio Φ_{ij} of per-capita reproductive values for Alpha and Delta. Within each wave, rows are ordered by increasing $\Pr(\Phi_{ij} \leq 0)$. Entries show the posterior median and 95% credible interval for Φ_{ij}. The last column gives the cumulative expected number of false discoveries up to that row, $\overline{\text{FD}} = \sum_{(i,j)\in S} \Pr(\Phi_{ij} \leq 0)$, where S is the set of pairs up to that row. Only the ten pairs with the smallest $\Pr(\Phi_{ij} \leq 0)$ are shown. With $\alpha = 0.05$, the largest S contains the pairs above the dashed line.

S.12 Other supplementary figures

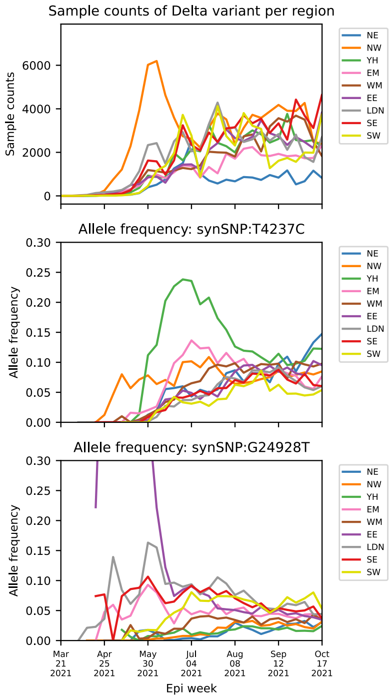

Fig. S24. Number of sequences of the Delta variant (top), and replot of the allele-frequency trajectories shown in Fig. 1B from an earlier time point (middle and bottom).

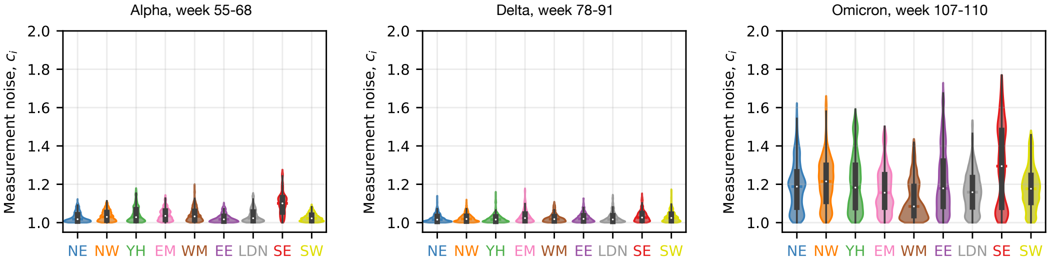

Fig. S25. The posterior distributions of the measurement noise parameters c_i for the Alpha, Delta, and Omicron variants in England, which are inferred by applying the HMM-MCMC method to the region-level mutation data.

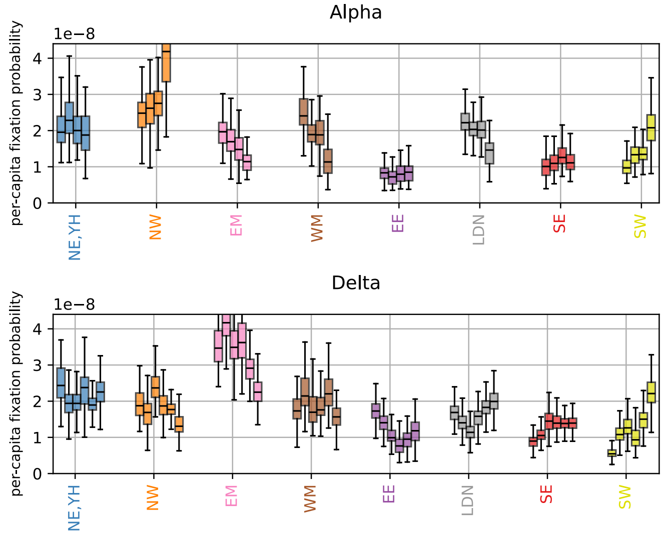

Fig. S26. Supplementary figure to Fig. 7C. The reproductive value in England is inferred at the regional level, with NE and YH being treated as a single region. For the Alpha wave, the time windows used for the four box plots are epiweek 54-67, 55-68, 56-69, and 57-70, respectively, from left to right. For the Delta wave, the time windows used for the six box plots are epiweek 78-91, 79-92, 80-93, 81-94, 82-95, and 83-96, respectively, from left to right. For each variant, we aggregated the results for all the time windows, and the aggregated results are shown in Fig. 7C. In the boxplots, the boxes represent the interquartile range (Q1 to Q3), with the central line indicating the median. The whiskers extend to $Q_1 - 1.5 \times \mathrm{IQR}$ and $Q_3 + 1.5 \times \mathrm{IQR}$.

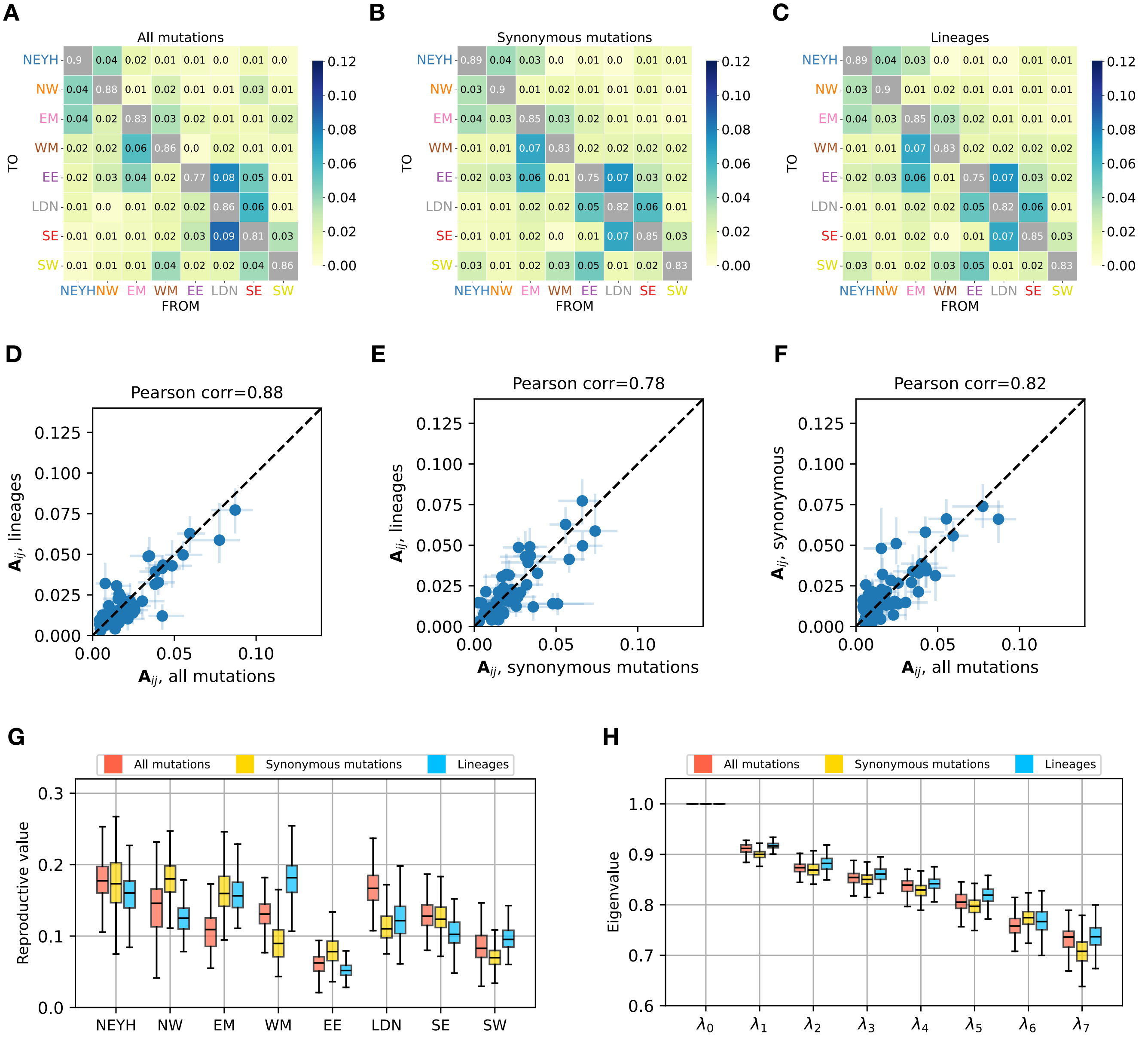

Fig. S27. The heatmaps (**A**-**C**) display region-level inferred matrices for the Delta wave (epiweeks 78-91) in England, where YH and NE regions are combined, for different data types: (**A**) mutation data, incorporating both synonymous and nonsynonymous mutations; (**B**) synonymous mutation data; (**C**) lineage data. The mean values of parameters, computed using MCMC, are presented in these heatmaps. (**D**-**F**) provide element-wise comparisons of these matrices, with error bars indicating standard deviations. (**G**) Comparison of the reproductive values π_i. (**H**) Comparison of the eigenvalues λ_i of the importation-rate matrix. In the boxplots, the boxes represent the interquartile range (Q1 to Q3), with the central line indicating the median. The whiskers extend to $Q_1 - 1.5 \times \mathrm{IQR}$ and $Q_3 + 1.5 \times \mathrm{IQR}$.

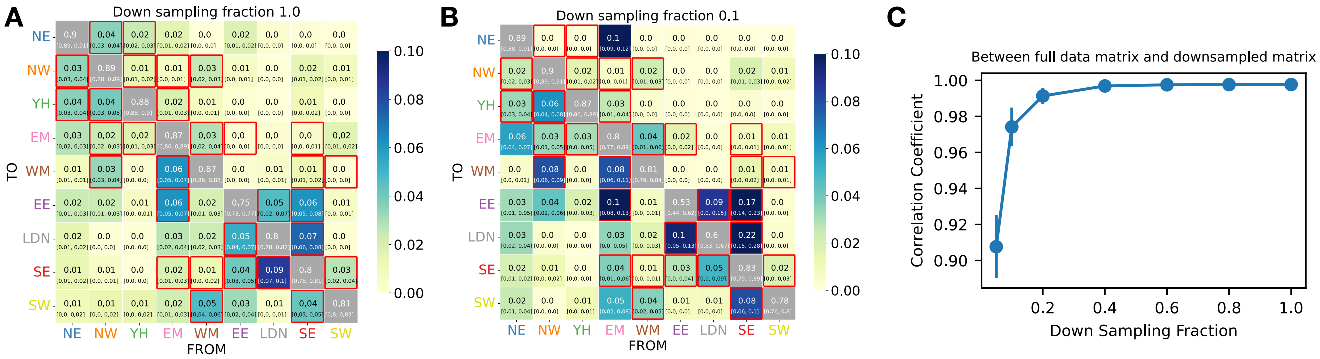

Fig. S28. (**A**) The importation-rate matrix for the Delta wave in England, inferred from the full dataset. The matrix is inferred using the EM algorithm, and the error indicates the lower and upper quartiles obtained from the bootstrapping method. (**B**) The importation-rate matrix inferred from 10% of the data. (**C**) The Pearson correlation coefficient between the matrix elements of the full dataset and those of the downsampled matrix is computed for downsampling fractions of $5\%, 10\%, 20\%, 40\%, 60\%, 80\%$, and 100% (i.e., the full dataset). The error bar indicates the standard deviation in the Pearson correlation coefficient obtained from the bootstrapping method. The matrix elements inferred from the downsampled data begin to deviate from those inferred from the full dataset when the fraction of downsampling is less than 10%.

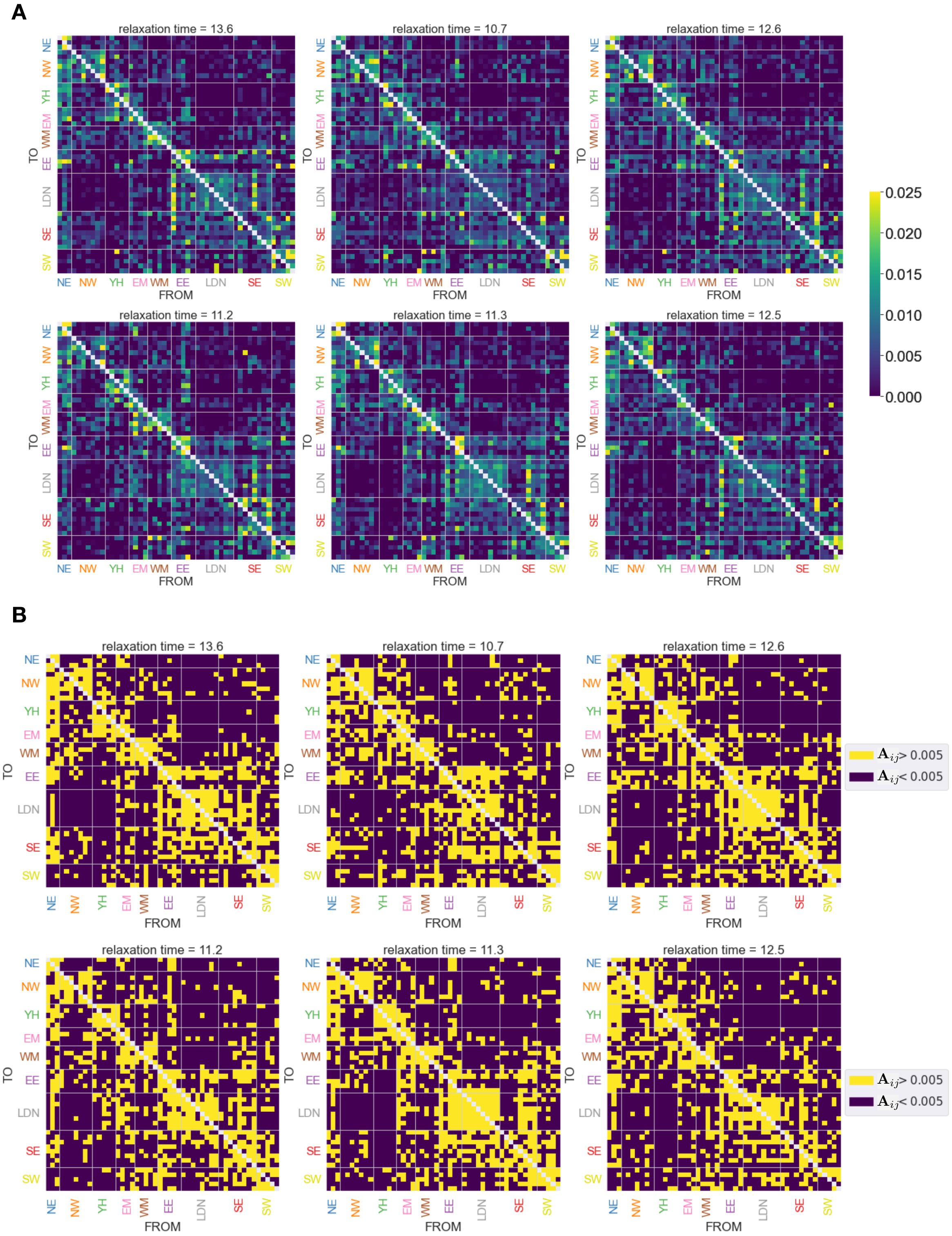

Fig. S29. (**A**) Six examples of transmission-rate matrices inferred for the Delta wave in England, obtained through the bootstrapping method. For each matrix, the relaxation time computed from the matrix is written on the heatmap. (**B**) Each of the six matrices from Fig. **A** is binarized with a threshold of $\mathbf{A}_{ij} = 0.005$.

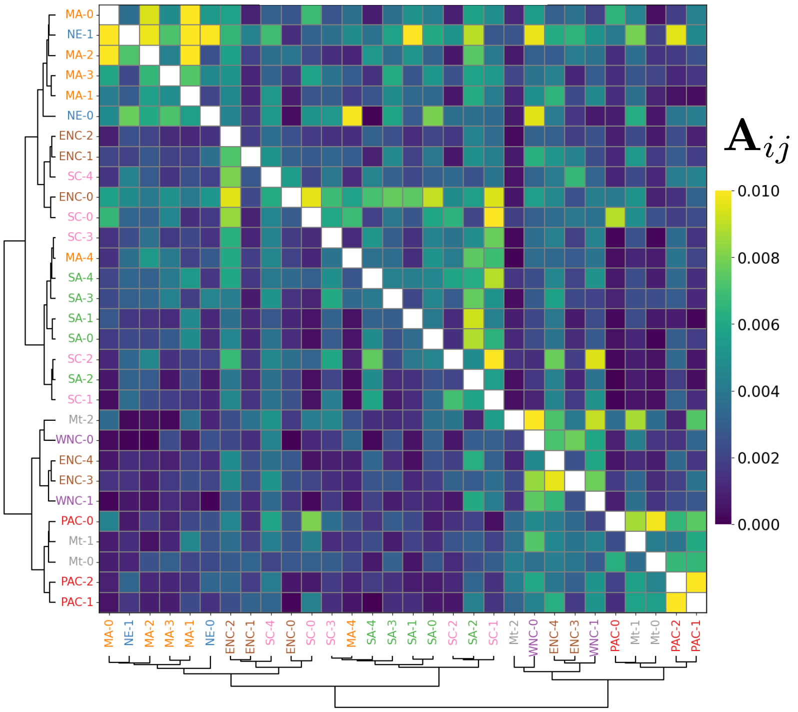

Fig. S30. Heat map of the importation-rate matrix $\mathbf{A}_{ij}$ for 30 demes of the USA during the Delta wave (epiweek 82-96). The main infection pathways are illustrated in Fig. 6 of the main text.

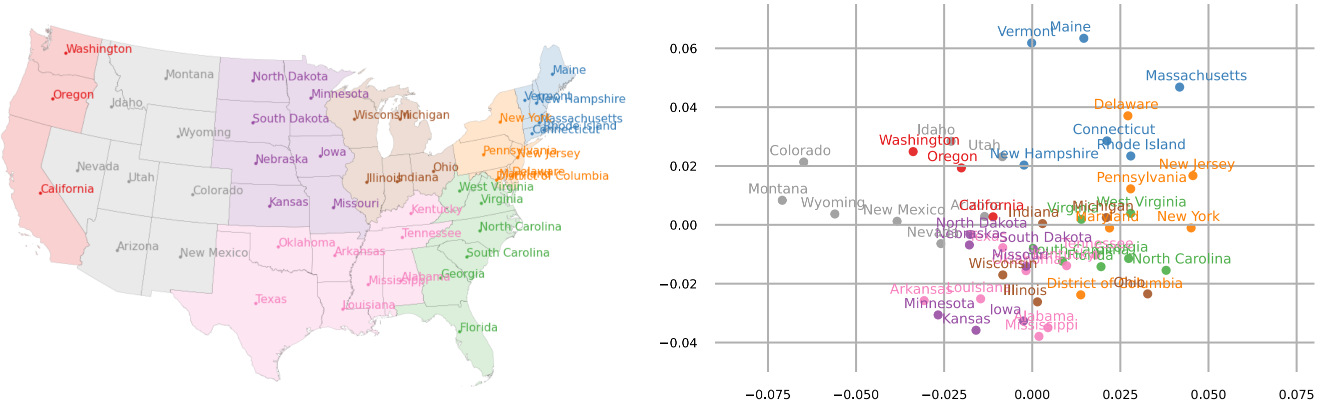

Fig. S31. Comparison between the actual geography and the multidimensional scaling plot for the state-level importation-rate matrix of the Delta wave in the US. The Spearman correlation between the physical distance and the square roots of Jensen-Shannon divergence is 0.52 (p-value $< 10^{-4}$).

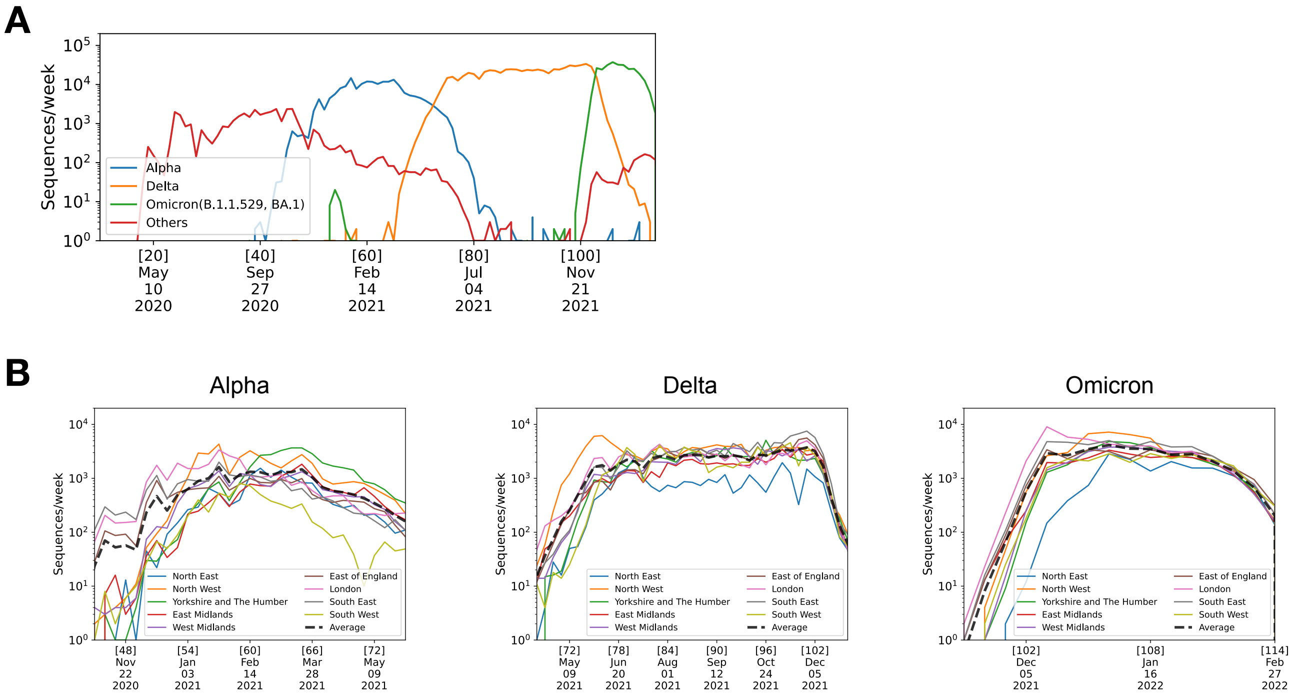

Fig. S32. (**A**) The number of sequences per week for each variant in England. The numbers [·] shown above dates in the horizontal axis indicate epiweeks since Dec/29/2021. (**B**) The weekly number of sequences in each region of England for Alpha, Delta, and Omicron. The numbers [·] shown above dates in the horizontal axis indicate epiweeks since Dec/29/2021.

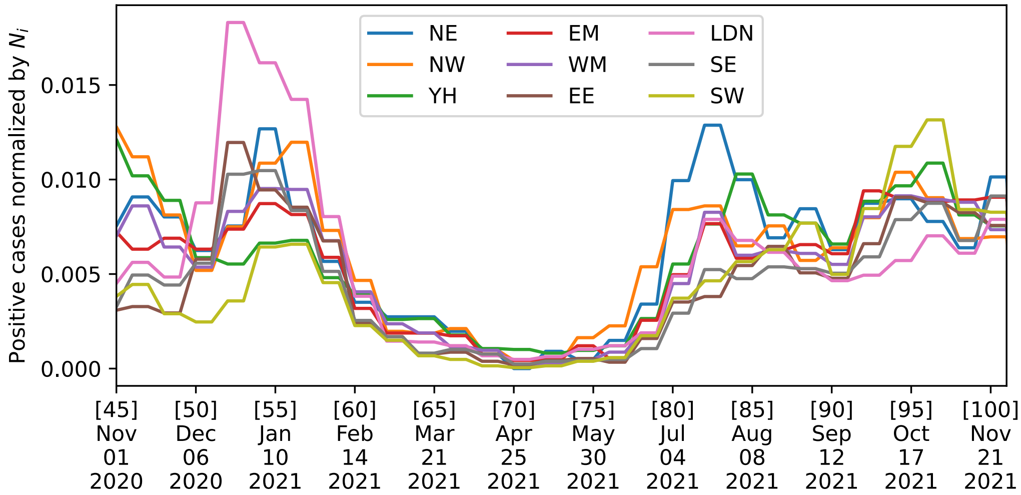

Fig. S33. The number of weekly infected individuals (estimated by surveillance testing (13)) divided by the population size across over time in the regions of England.

S.13 Construction of demes

S.13.1 Construction of demes in England. For the deme-resolved analysis of England, presented in Fig. 3, we constructed 50 demes from the upper tier local authorities (UTLAs). This involved clustering within each of the 9 regions of England in the following manner:

1. First, we select the 150 largest UTLAs based on the number s_u of metadata sequences reported from each UTLA u.
2. In each of the 9 regions, we create a connected graph using Delaunay Triangulation, using the geographic locations of these UTLAs. Edges that cross over the ocean are removed (Fig. S34A).

To create 50 demes with similar sizes, we determine the number n_i of demes to be assigned to each of the 9 regions using a greedy algorithm:

1. As the initial state, we set $n_i = 2$ for all regions i.
2. Assuming that, at an intermediate step of the algorithm, the 9 regions respectively have n_i demes. We define the weight of each region as $w_i = \frac{S_i}{n_i}$, where $S_i \equiv \sum_{u \in \text{region } i} s_u$ is the total number of sequences in region i. We then increase n_i by 1 for the region with the smallest weight.
3. This process is repeated until the sum of n_i across all regions reaches the target number of demes, 50. The resulting allocation of demes was $n_i = 3, 7, 5, 4, 5, 5, 8, 8, 5$ for $i =$ NE, NW, YH, EM, WM, EE, LDN, SE, SW, respectively.

Finally, for each region i, we organize n demes (where the subscript i of n_i is omitted for notational simplicity) by grouping the UTLAs in the region into non-overlapping sets $U_1, \dots, U_n$:

1. Suppose that region i includes M UTLAs in the graph, labeled by u_α ($\alpha = 1, \dots, M$). By arranging the UTLAs, we may assume that $s_1 \geq s_2 \geq \dots \geq s_M$, where s_α is the number of sequences reported from UTLA u_α.
2. We initialize the sets U_k with the first n UTLAs, namely, the largest n UTLAs, as $U_k = \{u_k\}$ ($k = 1, \dots, n$).
3. For the remaining UTLAs $u_{n+1}, \dots, u_M$, we add each UTLA one by one to its nearest set on the graph constructed from the Delaunay Triangulation. Here, the distance between UTLA u and set U_k is defined as $\min_{u' \in U_k} d_{u,u'}$, where $d_{u,u'}$ is the graph distance; for example, the distance is 1 for adjacent UTLAs.
4. If the nearest set for UTLA u is not unique, we add it to the set with the fewest sequences, to mitigate the imbalance in deme sizes.

The resulting demes are shown in Fig. S34B and summarized in Table 4.

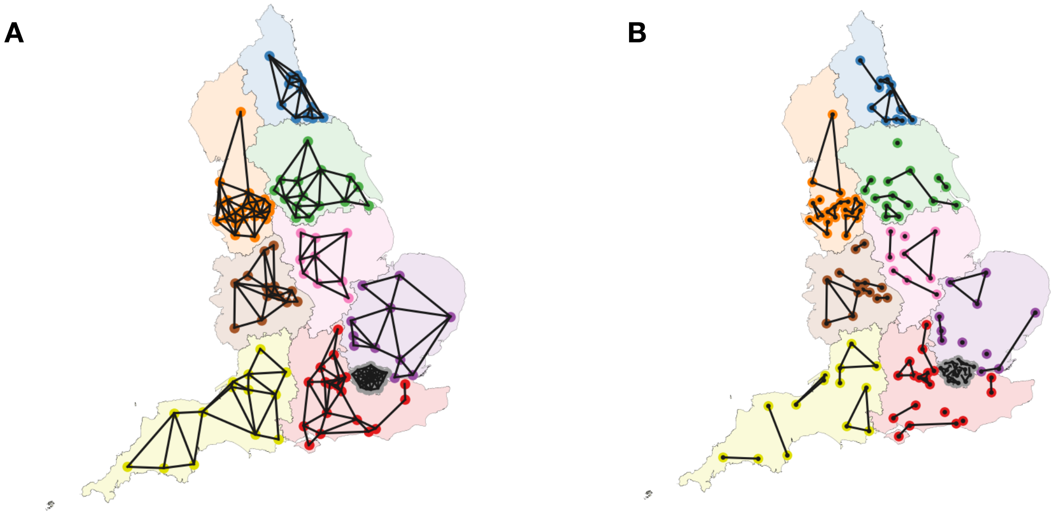

Fig. S34. (**A**) The nodes represent the top 150 UTLAs with the highest number of sequences in the metadata. Within each region, a graph is constructed using Delaunay triangulation. (**B**) The UTLAs in each region are grouped into n_i demes, using the information of the number of sequences. The 50 demes constructed in this manner are shown as connected components.

	deme	UTLAs
0	NE-0	County Durham; Sunderland; Stockton-on-Tees; Middlesbrough; Darlington
1	NE-1	Northumberland; Gateshead
2	NE-2	Newcastle upon Tyne; North Tyneside; South Tyneside; Redcar and Cleveland; Hartlepool
3	NW-0	Lancashire
4	NW-1	Manchester; Salford; Oldham; Rochdale
5	NW-2	Liverpool; Sefton; Knowsley
6	NW-3	Cumbria; Blackburn with Darwen; Blackpool
7	NW-4	Cheshire East; Stockport; Trafford; Tameside
8	NW-5	Cheshire West and Chester; Wirral; Halton
9	NW-6	Wigan; Bolton; Warrington; Bury; St. Helens
10	YH-0	Leeds; York; North Lincolnshire; North East Lincolnshire
11	YH-1	North Yorkshire; East Riding of Yorkshire; Kingston upon Hull, City of
12	YH-2	Sheffield; Rotherham; Barnsley
13	YH-3	Bradford; Calderdale
14	YH-4	Kirklees; Wakefield; Doncaster
15	EM-0	Nottinghamshire
16	EM-1	Derbyshire; Derby
17	EM-2	Lincolnshire; Nottingham; Rutland
18	EM-3	Northamptonshire; Leicestershire; Leicester
19	WM-0	Birmingham; Sandwell
20	WM-1	Staffordshire; Stoke-on-Trent
21	WM-2	Worcestershire; Shropshire; Dudley; Herefordshire, County of
22	WM-3	Warwickshire; Walsall; Wolverhampton; Telford and Wrekin
23	WM-4	Coventry; Solihull
24	EE-0	Essex
25	EE-1	Hertfordshire
26	EE-2	Suffolk; Southend-on-Sea; Thurrock
27	EE-3	Cambridgeshire; Norfolk; Peterborough
28	EE-4	Central Bedfordshire; Luton; Bedford
29	LDN-0	Barnet; Camden; Haringey; Harrow
30	LDN-1	Croydon; Sutton; Merton; Richmond upon Thames; Hammersmith and Fulham; Kingston upon Thames
31	LDN-2	Newham; Greenwich; Hackney
32	LDN-3	Ealing; Brent; Hillingdon; Hounslow
33	LDN-4	Enfield; Redbridge; Waltham Forest; Havering
34	LDN-5	Bromley; Lewisham; Bexley; Barking and Dagenham
35	LDN-6	Tower Hamlets; Southwark; City of London
36	LDN-7	Wandsworth; Lambeth; Westminster; Islington; Kensington and Chelsea
37	SE-0	Kent; Medway
38	SE-1	Hampshire; Southampton
39	SE-2	West Sussex
40	SE-3	Oxfordshire; Reading; West Berkshire
41	SE-4	East Sussex
42	SE-5	Buckinghamshire; Milton Keynes; Slough
43	SE-6	Surrey; Wokingham; Windsor and Maidenhead; Bracknell Forest
44	SE-7	Brighton and Hove; Portsmouth; Isle of Wight
45	SW-0	Devon; Torbay
46	SW-1	Gloucestershire; South Gloucestershire; Swindon; Bath and North East Somerset
47	SW-2	Cornwall; Plymouth
48	SW-3	Somerset; Bristol, City of; North Somerset
49	SW-4	Wiltshire; Bournemouth, Christchurch and Poole; Dorset

Table 4. List of 50 demes in England.

S.13.2 Construction of demes in the US.

In the US analysis, shown in Fig. 6, we constructed 30 demes using an approach similar to that used in the England analysis: Since the USA has no direct counterpart to the regions of England, we defined 8 regions in the USA for the purpose of the analysis: NE, MA, SA, SC, ENC, WNC, Mt, PAC, as colored in Fig. 6B. We then performed the same clustering as in the England analysis, making the following replacements: 150 UTLAs $\rightarrow$ 49 states (excluding Hawaii and Alaska) and 50 demes $\rightarrow$ 30 demes. In addition, instead of using Delaunay Triangulation, we constructed the graph of the US states based on whether they are geographically neighboring. The resulting demes are summarized in Table 5.

	deme	States
0	NE-0	Massachusetts,Maine,New Hampshire,Vermont
1	NE-1	Connecticut,Rhode Island
2	MA-0	New York
3	MA-1	Pennsylvania
4	MA-2	New Jersey
5	MA-3	Maryland,District of Columbia
6	MA-4	Delaware
7	SA-0	Florida
8	SA-1	Georgia
9	SA-2	North Carolina
10	SA-3	Virginia,West Virginia
11	SA-4	South Carolina
12	SC-0	Texas,Oklahoma
13	SC-1	Tennessee,Arkansas
14	SC-2	Alabama
15	SC-3	Louisiana,Mississippi
16	SC-4	Kentucky
17	ENC-0	Illinois
18	ENC-1	Ohio
19	ENC-2	Michigan
20	ENC-3	Indiana
21	ENC-4	Wisconsin
22	WNC-0	Missouri,Kansas
23	WNC-1	Minnesota,Iowa,Nebraska,South Dakota,North Dakota
24	Mt-0	Arizona
25	Mt-1	Colorado,New Mexico,Wyoming
26	Mt-2	Utah,Nevada,Idaho,Montana
27	PAC-0	California
28	PAC-1	Washington
29	PAC-2	Oregon

Table 5. List of 30 demes in the US.

S.14 Calendar Dates and Weeks Since December 29, 2019

Epiweek	Date
1	Dec 29, 2019
2	Jan 05, 2020
3	Jan 12, 2020
4	Jan 19, 2020
5	Jan 26, 2020
6	Feb 02, 2020
7	Feb 09, 2020
8	Feb 16, 2020
9	Feb 23, 2020
10	Mar 01, 2020
11	Mar 08, 2020
12	Mar 15, 2020
13	Mar 22, 2020
14	Mar 29, 2020
15	Apr 05, 2020
16	Apr 12, 2020
17	Apr 19, 2020
18	Apr 26, 2020
19	May 03, 2020
20	May 10, 2020
21	May 17, 2020
22	May 24, 2020
23	May 31, 2020
24	Jun 07, 2020
25	Jun 14, 2020
26	Jun 21, 2020
27	Jun 28, 2020
28	Jul 05, 2020
29	Jul 12, 2020
30	Jul 19, 2020
31	Jul 26, 2020
32	Aug 02, 2020
33	Aug 09, 2020
34	Aug 16, 2020
35	Aug 23, 2020
36	Aug 30, 2020
37	Sep 06, 2020

Epiweek	Date
38	Sep 13, 2020
39	Sep 20, 2020
40	Sep 27, 2020
41	Oct 04, 2020
42	Oct 11, 2020
43	Oct 18, 2020
44	Oct 25, 2020
45	Nov 01, 2020
46	Nov 08, 2020
47	Nov 15, 2020
48	Nov 22, 2020
49	Nov 29, 2020
50	Dec 06, 2020
51	Dec 13, 2020
52	Dec 20, 2020
53	Dec 27, 2020
54	Jan 03, 2021
55	Jan 10, 2021
56	Jan 17, 2021
57	Jan 24, 2021
58	Jan 31, 2021
59	Feb 07, 2021
60	Feb 14, 2021
61	Feb 21, 2021
62	Feb 28, 2021
63	Mar 07, 2021
64	Mar 14, 2021
65	Mar 21, 2021
66	Mar 28, 2021
67	Apr 04, 2021
68	Apr 11, 2021
69	Apr 18, 2021
70	Apr 25, 2021
71	May 02, 2021
72	May 09, 2021
73	May 16, 2021
74	May 23, 2021

Epiweek	Date
75	May 30, 2021
76	Jun 06, 2021
77	Jun 13, 2021
78	Jun 20, 2021
79	Jun 27, 2021
80	Jul 04, 2021
81	Jul 11, 2021
82	Jul 18, 2021
83	Jul 25, 2021
84	Aug 01, 2021
85	Aug 08, 2021
86	Aug 15, 2021
87	Aug 22, 2021
88	Aug 29, 2021
89	Sep 05, 2021
90	Sep 12, 2021
91	Sep 19, 2021
92	Sep 26, 2021
93	Oct 03, 2021
94	Oct 10, 2021
95	Oct 17, 2021
96	Oct 24, 2021
97	Oct 31, 2021
98	Nov 07, 2021
99	Nov 14, 2021
100	Nov 21, 2021
101	Nov 28, 2021
102	Dec 05, 2021
103	Dec 12, 2021
104	Dec 19, 2021
105	Dec 26, 2021
106	Jan 02, 2022
107	Jan 09, 2022
108	Jan 16, 2022
109	Jan 23, 2022
110	Jan 30, 2022
111	Feb 06, 2022

S.15 Mutation sets of SARS-CoV-2 Variants in England and the US

Index	Alpha England	Delta England	Omicron England	Delta US
1	synSNP:G4255A	synSNP:G24928T	orf1ab:L1130F	NSP6_C197F
2	synSNP:C12970T	orf1ab:S2048F	synSNP:T27384C	NSP16_Q238H
3	orf1ab:P4619L	orf1ab:L3606F	synSNP:G5515T	NSP14_A323S
4	S:L5F	synSNP:C19524T	synSNP:C9487T	N_A208S
5	ORF3a:G224C	synSNP:G17427T	synSNP:C16887T	NSP9_R39K
6	orf1ab:T5956I	synSNP:A1741G	orf1ab:T6833I	NSP2_G339S
7	orf1ab:L3829F	synSNP:G22051C	orf1ab:L3606F	NS8_P36S
8	synSNP:C27513T	synSNP:G10870T	synSNP:C10138T	NSP14_H26Y
9	M:A2S	orf1ab:G661S	S:L5F	Spike_L5F
10	synSNP:C14805T	S:D138H	S:E309Q	NSP5_K90R
11	orf1ab:G1125C	ORF7a:H73Y	orf1ab:P1803S	M_T30I
12	orf1ab:A1314V	orf1ab:V2766F	orf1ab:D4813N	NS8_R115C
13	orf1ab:K3353R	synSNP:C4891T	synSNP:C29614T	NSP15_A80V
14	M:V70L	orf1ab:E1909A	orf1ab:V3917I	NSP2_T223I
15	orf1ab:L3606F	synSNP:C8266T	synSNP:C26873T	NS8_D119del
16	synSNP:C9712T	synSNP:C14724T	orf1ab:T3058I	N_D63S
17	orf1ab:P5402S	ORF8:S67F	orf1ab:K6958R	NSP16_K160R
18	orf1ab:T2124I	synSNP:T18876C	orf1ab:Y5593C	NSP8_T141M
19	orf1ab:R3012K	S:L5F	S:A831V	NSP4_S209F
20	synSNP:C16887T	orf1ab:T814I	synSNP:C9857T	NSP3_T1022I
21	synSNP:A8512G	orf1ab:S443F	synSNP:T4579A	NSP6_A2V
22	S:S98F	synSNP:C601T	synSNP:T26978C	NS8_Q72H
23	synSNP:C19983T	synSNP:T13210C	synSNP:C337T	NS3_W131C
24	synSNP:T12388C	synSNP:G29179T	synSNP:C4891T	N_S327L
25	synSNP:C29272T	orf1ab:L3086F	orf1ab:K3353R	NSP5_I259L
26	ORF3a:T175I	orf1ab:A339V	orf1ab:T4174I	NSP3_A1711V
27	ORF3a:S171L	synSNP:C22000T	ORF7a:E95*	NSP2_R27C
28	synSNP:C22432T	synSNP:G29254T	orf1ab:S944L	N_T362I
29	S:G142V	orf1ab:D6249N		NS8_S67F
30	orf1ab:D4545Y	synSNP:T25959C		Spike_Q613H
31	synSNP:C27059T	synSNP:T21835C		NSP3_S1230F
32	orf1ab:P309L	synSNP:C15237T		NSP2_A411V
33	synSNP:C823T	synSNP:C16887T		NSP3_T936I
34	synSNP:C2137T	orf1ab:M3712I		N_K373N
35	orf1ab:D4501Y	orf1ab:D6300N		NSP3_S126L
36	orf1ab:S2103F	ORF3a:W128L		N_T135I
37	orf1ab:S944L	synSNP:C2485T		NSP5_L220F
38	synSNP:C28603T	orf1ab:D5271Y		NSP15_I305V
39	synSNP:C25006T	orf1ab:A1298V		Spike_T572I
40	orf1ab:E2607K	orf1ab:T1429I		NSP14_A100S
41	synSNP:C15960T	synSNP:C4456T		NS3_L101I
42	synSNP:G28878C	synSNP:C25614T		NSP4_S76N
43	synSNP:C25572T	synSNP:T29288C		NS3_L95S
44	synSNP:C3811T	synSNP:C5392T		NS8_A65S
45	ORF3a:H78Y	orf1ab:V1783F		Spike_R214H
46	synSNP:C26936T	synSNP:C7420T		NSP3_K280N
47	synSNP:C18555T	N:P383S		NS7a_Q62stop
48	synSNP:G28396A	N:P365L		E_V62F
49	synSNP:C29095T	orf1ab:K6958R		NS3_A110S
50	synSNP:C4573T	synSNP:C21595T		NSP2_I514T
51	S:D138H	synSNP:G21123T		NS3_P42L
52	synSNP:T27384C	orf1ab:S100N		NSP4_I385V
53	synSNP:C29614T	synSNP:C4331T		N_A152S
54	ORF7a:P84S	ORF7a:G38*		Spike_K1073N
55	synSNP:C17502T	orf1ab:A6044V		NSP4_T295I
56	orf1ab:T6938I	N:G238C		NSP15_A171S
57	synSNP:C26681T	S:L18F		NSP1_R24C
58	synSNP:C10252T	S:G1124V		NS3_W69C
59	synSNP:C292T	orf1ab:P271L		Spike_Q677H
60	orf1ab:A3697V	synSNP:C25452T		
61	orf1ab:K6958R	synSNP:G6352A		
62	synSNP:C25521T	orf1ab:T403I		
63	synSNP:C23683T	orf1ab:L6909F		
64	synSNP:C22480T	synSNP:C19983T		
65	synSNP:T24331A	orf1ab:A2785V		
66	synSNP:C26801T	ORF7a:A50S		
67	synSNP:T10253C	orf1ab:M4855I		
68	synSNP:C21727T	orf1ab:T5923I		
69	E:V58F			
70	synSNP:C10156T			
71	synSNP:C23833T			
72	orf1ab:S5398L			
73	synSNP:C11455T			
74	ORF8:A65V			
75	synSNP:C7528T			
76	S:T95I			
77	synSNP:A28856C			

S.16 Legend of Supplementary Movie of Figure 8 in the Main Text

The observed frequencies of the Delta variant in England (from March 7, 2021, to July 10, 2021), along with simulated frequencies based on the inferred importation-rate matrix and a matrix excluding long-range importations (i.e., a matrix with zero entries for couplings across non-neighboring regions), are displayed on a geographical map of England. For each UTLA, the frequency of the Delta variant in the deme containing the UTLA is shown. The colors represent the logit of the frequency, $\log_{10} \frac{f_i(t)}{1-f_i(t)}$, where $f_i(t)$ denotes the frequency of the Delta variant in deme i at time t.

References

1. The COVID-19 Genomics UK (COG-UK) consortium, An integrated national scale SARS-CoV-2 genomic surveillance network. *The Lancet Microbe* **1**, e99 (2020) https://doi.org/10.1016/S2666-5247(20)30054-9.
2. UK Health Security Agency, Sars-cov-2 variants of concern and variants under investigation in england: Technical briefing 26 (2021).
3. M Alkhatib, et al., Update on sars-cov-2 omicron variant of concern and its peculiar mutational profile. *Microbiol. Spectr.* **10**, e02732–21 (2022).
4. Á O'Toole, et al., Assignment of epidemiological lineages in an emerging pandemic using the pangolin tool. *Virus Evol.* **7**, veab064 (2021).
5. A Rambaut, et al., A dynamic nomenclature proposal for SARS-CoV-2 lineages to assist genomic epidemiology. *Nat. microbiology* **5**, 1403–1407 (2020).
6. Q Yu, et al., Lineage frequency time series reveal elevated levels of genetic drift in sars-cov-2 transmission in england. *Plos Pathog.* **20**, e1012090 (2024).
7. P Barrat-Charlaix, J Huddleston, T Bedford, RA Neher, Limited predictability of amino acid substitutions in seasonal influenza viruses. *Mol. Biol. Evol.* **38**, 2767–2777 (2021).
8. JA Ascensao, KM Wetmore, BH Good, AP Arkin, O Hallatschek, Quantifying the local adaptive landscape of a nascent bacterial community. *Nat. Commun.* **14**, 248 (2023).
9. Safegraph social distancing metrics (https://docs.safegraph.com/docs/social-distancing-metrics) (2021).
10. G Le Treut, et al., A high-resolution flux-matrix model describes the spread of diseases in a spatial network and the effect of mitigation strategies. *Sci. Reports* **12**, 15946 (2022).
11. CM Bishop, *Pattern recognition and machine learning.* (Springer) Vol. 4, (2006).
12. F Noé, Probability distributions of molecular observables computed from markov models. *The J. chemical physics* **128** (2008).
13. UK Office for National Statistics, Coronavirus (COVID-19) Infection Survey: England (https://www.ons.gov.uk/peoplepopulationandcommunity/healthandsocialcare/conditionsanddiseases/datasets/coronaviruscovid19infectionsurveydata) (2021).
14. P Sagulenko, V Puller, RA Neher, Treetime: Maximum-likelihood phylodynamic analysis. *Virus evolution* **4**, vex042 (2018).
15. AL Lloyd, RM May, Spatial heterogeneity in epidemic models. *J. theoretical biology* **179**, 1–11 (1996).
16. HW Hethcote, An immunization model for a heterogeneous population. *Theor. population biology* **14**, 338–349 (1978).
17. W Post, D DeAngelis, C Travis, Endemic disease in environments with spatially heterogeneous host populations. *Math. Biosci.* **63**, 289–302 (1983).
18. H McCallum, N Barlow, J Hone, How should pathogen transmission be modelled? *Trends ecology & evolution* **16**, 295–300 (2001).
19. D Miller, et al., Full genome viral sequences inform patterns of SARS-CoV-2 spread into and within Israel. *Nat. Commun.* **11**, 1–10 (2020).
20. WS Hart, et al., Generation time of the alpha and delta sars-cov-2 variants: an epidemiological analysis. *The Lancet Infect. Dis.* **22**, 603–610 (2022).
21. P Müller, G Parmigiani, K Rice, Fdr and bayesian multiple comparisons rules in *Bayesian Statistics 8: Proceedings of the Eighth Valencia International Meeting, June 2–6, 2006*, eds. JM Bernardo, et al. (Oxford University Press, Oxford), pp. 349–370 (2007) Online ed., Oxford Academic, 2023-10-31.
